# Supplementary material for: Interaction of chikungunya virus glycoproteins with macrophage factors controls virion production
Source: EMBO J. 2024 Sep 11;43(20):4625–55. doi: 10.1038/s44318-024-00193-3 (PMC11480453; doi:10.1038/s44318-024-00193-3)
Supplement: Supplementary file 2 — Appendix [file 44318_2024_193_MOESM2_ESM.pdf]

Interaction of chikungunya virus glycoproteins with macrophage factors controls virion production

Zhenlan Yao[1], Sangeetha Ramachandran[1], Serina Huang[2], Erin Kim[3], Yasaman Jami-Alahmadi[4], Prashant Kaushal[1,5,6], Mehdi Bouhaddou[1,5,6], James A. Wohlschlegel[4], Melody M.H. Li[1]

[1]Department of Microbiology, Immunology and Molecular Genetics, University of California, Los Angeles, Los Angeles, CA, USA
[2]Department of Human Genetics, University of California, Los Angeles, Los Angeles, CA, USA
[3]Department of Chemistry and Biochemistry, University of California, Los Angeles, Los Angeles, CA, USA
[4]Department of Biological Chemistry, University of California, Los Angeles, Los Angeles, CA, USA
[5]Institute for Quantitative and Computational Biosciences, University of California, Los Angeles, Los Angeles, CA, USA
[6]Molecular Biology Institute, University of California Los Angeles, Los Angeles, CA, USA

***Correspondence:** Manhingli@mednet.ucla.edu

Appendix Figure S1 Page 1

Appendix Figure S2 Page 2

Appendix Figure S1

A

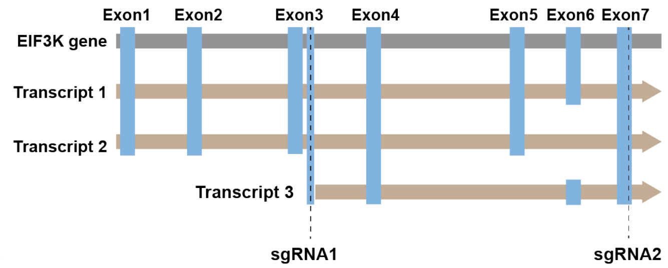

B

Wild-type (sgRNA 1 target)	TGTGCAAGTGCATGATCGA----------CCAGGCACATGTATCCTTCCAGCACTGGGGCCGGGGGGTGTGTGGGAAGGGGTCAGAGTCAAGGTG
Clone 7 allele 1 (sgRNA 1 target)	TGTGCAAGTG--CAAGGTG
Clone 7 allele 2 (sgRNA 1 target)	TGTGCAAGTG--------------------------CATGTATCCTTCCAGCACTGGGGCCGGGGGGTGTGTGGGAAGGGGTCAGAGTCAAGGTG
Clone 9 allele 1 (sgRNA 1 target)	TGTGCAAGTGCATGTGCAAGTGCAGGATACCAGGCACATGTATCCTTCCAGCACTGGGGCCGGGGGGTGTGTGGGAAGGGGTCAGAGTCAAGGTG
Clone 9 allele 2 (sgRNA 1 target)	TGT--------------------------CCAGGCACATGTATCCTTCCAGCACTGGGGCCGGGGGGTGTGTGGGAAGGGGTCAGAGTCAAGGTG
Wild-type (sgRNA 2 target)	AGCTAAAGGTGTGGATGAGCAAATACGGCTGGAGTGCCGACGAGTCGGGGCAGATCTTCATCTGTAGCCAAGAAGAGAGCATTAAACCCAAGAAC
Clone 7 allele 1 (sgRNA 2 target)	AGCTAAAGGTGTGGATGAGCAAATACGGCTGGAGTGCCGACGTAT-GGGGCAGATCTTCATCTGTAGCCAAGAAGAGAGCATTAAACCCAAGAAC

Appendix Figure S1. CRISPR/Cas9-mediated KO of *EIF3K* in 293T cells.

A. The schematic of EIF3K CRISPR/Cas9-mediated editing strategy to generate KO single cell clones in 293T cells. sgRNAs #1 and #2 target exons 3 and7 of EIF3K, respectively, which destroy expression of all 3 transcripts.

B. Validation of EIF3K KO 293T single cell clones. Sequencing of EIF3K KO clone 7 genomic DNA showed mutations in both sgRNAs #1 and #2 targeted regions. Sequencing of clone 9 genomic DNA only detected deletion and insertion in sgRNA #1-targeted region. No sequences of sgRNA #2-targeted region were detected, which is likely due to large deletion at the end of the EIF3K transcript. The complete KO of EIF3K in clones 7 and 9 were also confirmed by immunoblotting (Figure 8A).

Appendix Figure S2

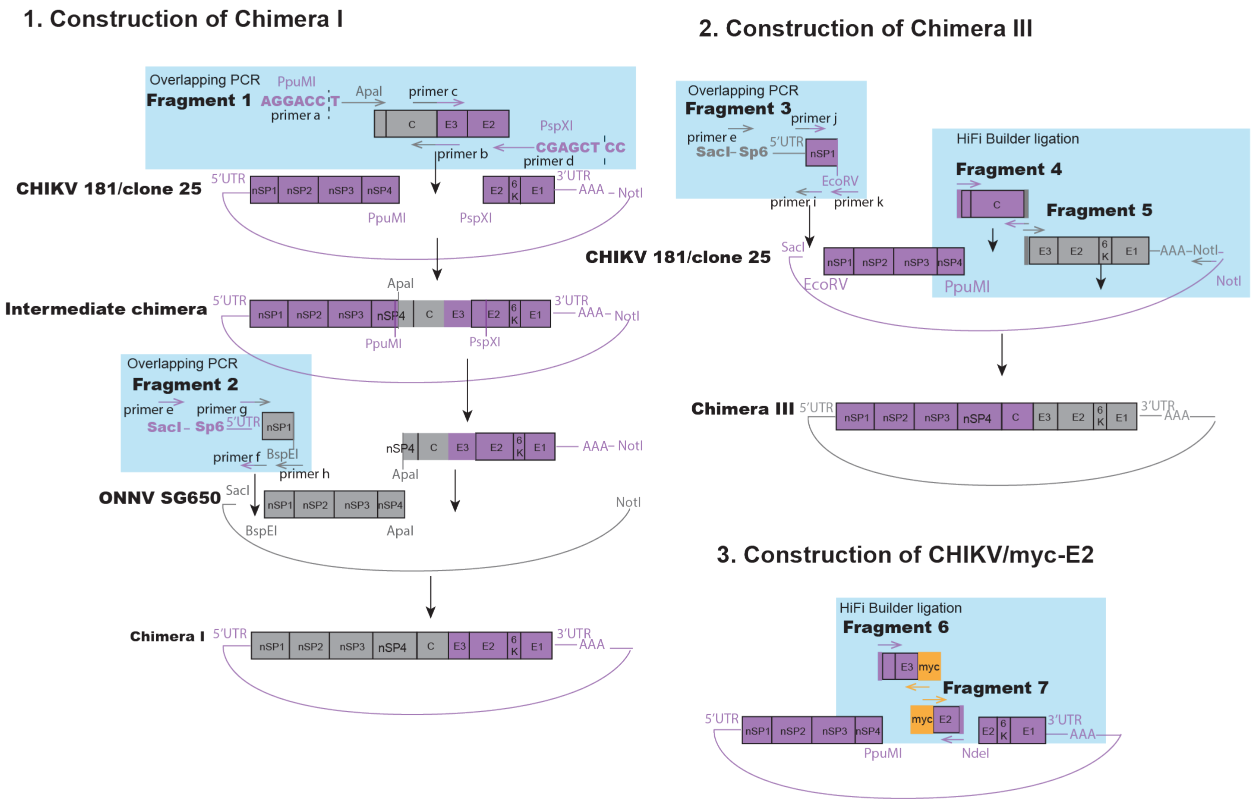

Appendix Figure S2. Construction workflows for Chimera I, Chimera III and CHIKV/myc-E2.
