# Supplementary material for: Interaction of chikungunya virus glycoproteins with macrophage factors controls virion production
Source: EMBO J. 2024 Sep 11;43(20):4625–55. doi: 10.1038/s44318-024-00193-3 (PMC11480453; doi:10.1038/s44318-024-00193-3)
Supplement: Supplementary file 5 — Source data Fig. 1 [file 44318_2024_193_MOESM5_ESM.zip › Figure 1/1A/Data description.docx]

The experiment was done in biological duplicates, and representative replicates are shown in formal figure 1A.

The mqd files are raw flow cytometry data of representative replicates in figure 1A.

The wsp file summarized the gating and analysis information of all biological duplicates in this experiment.
