# Supplementary material for: Interaction of chikungunya virus glycoproteins with macrophage factors controls virion production
Source: EMBO J. 2024 Sep 11;43(20):4625–55. doi: 10.1038/s44318-024-00193-3 (PMC11480453; doi:10.1038/s44318-024-00193-3)

## CHIKV 8h transfection

Dilution: 100 1000 10000

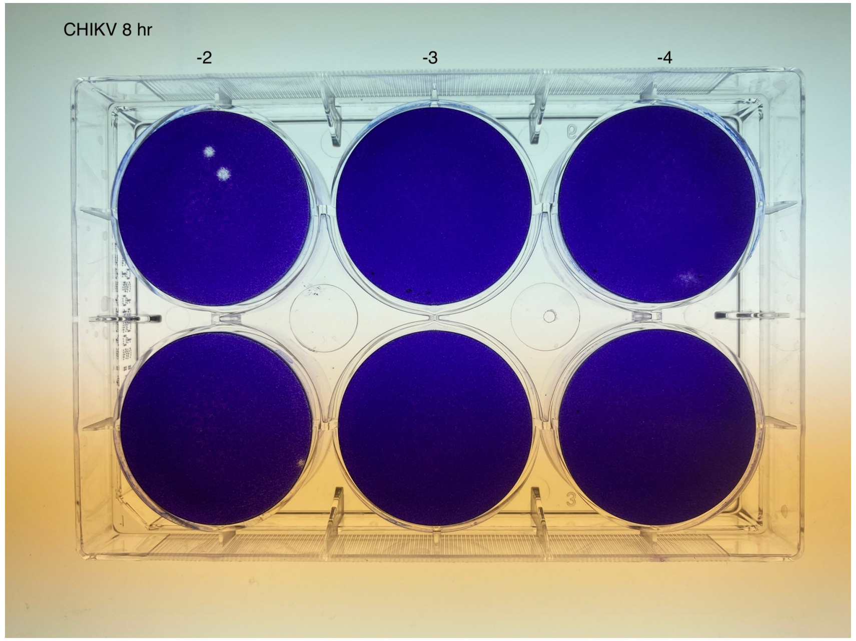


## CHIKV 14h transfection

Dilution: 100 1000 10000

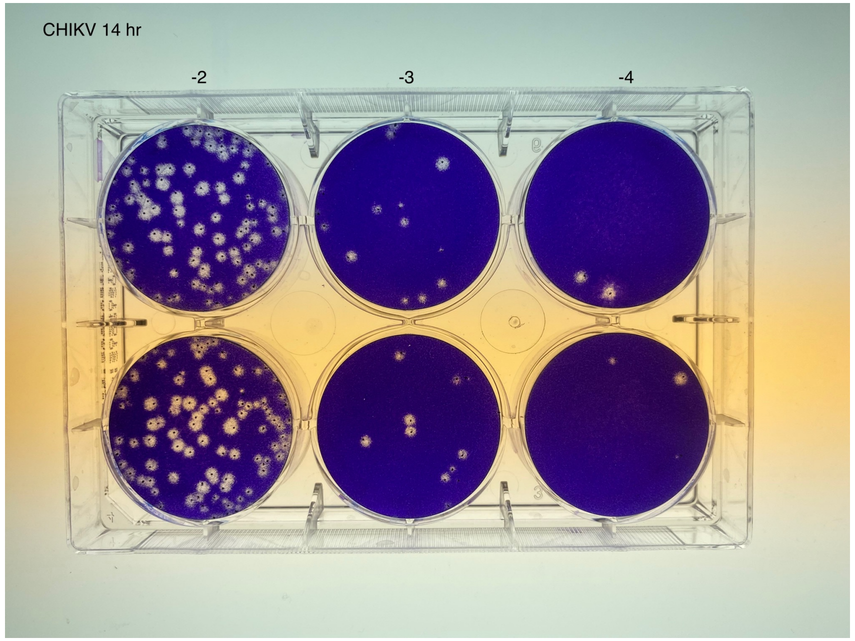


## CHIKV 24h transfection

Dilution: 100 1000 10000

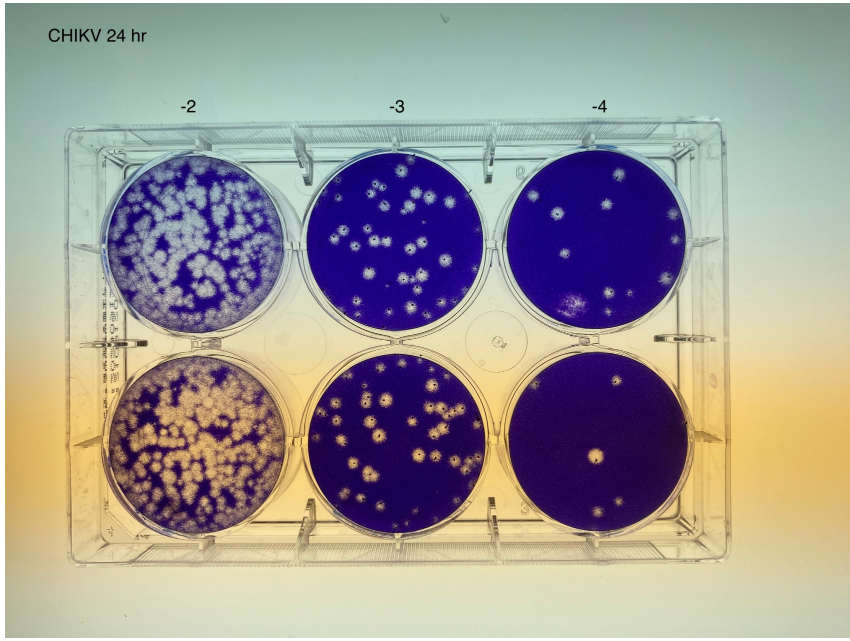

## ONNV 8h transfection

Dilution: 100 1000 10000

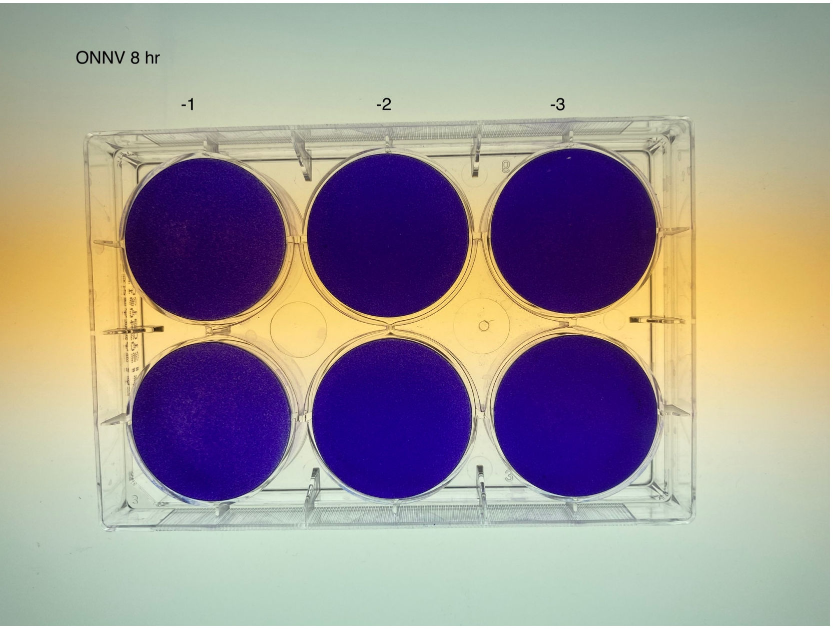


## ONNV 14h transfection

Dilution: 100 1000 10000

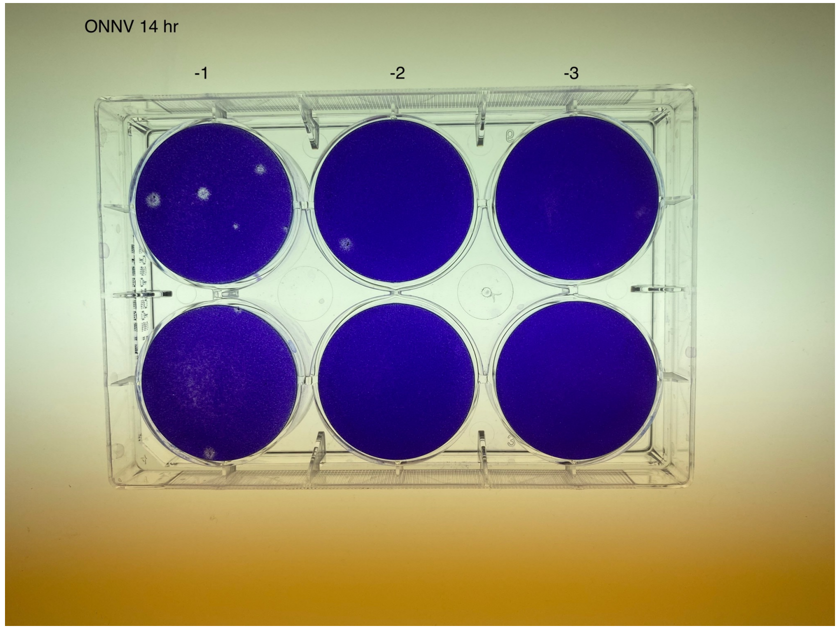


## ONNV 24h transfection

Dilution: 100 1000 10000

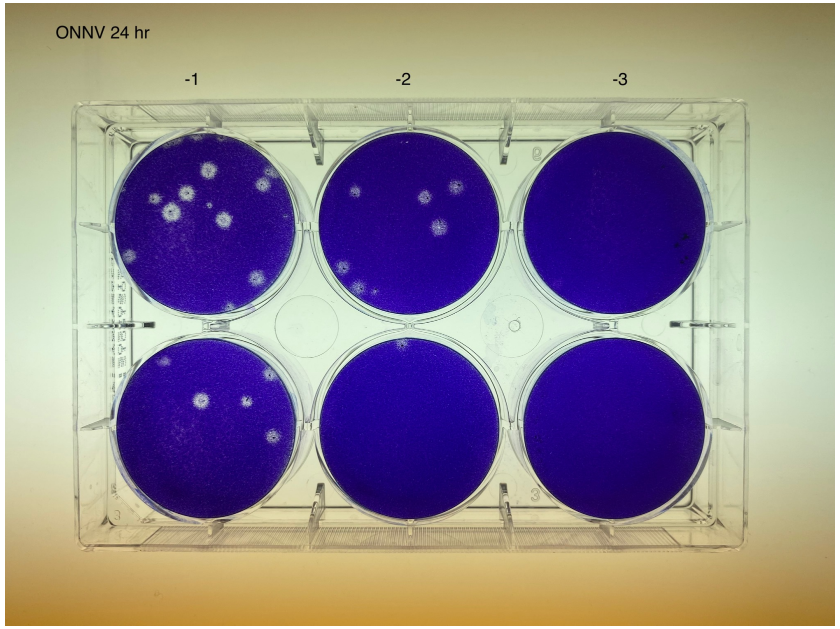

Supplement: Supplementary file 5 — Source data Fig. 1 [file 44318_2024_193_MOESM5_ESM.zip › Figure 1/1D/1D images with description.pdf]
