# Supplementary figures and images for: Interaction of chikungunya virus glycoproteins with macrophage factors controls virion production
Source: EMBO J. 2024 Sep 11;43(20):4625–55. doi: 10.1038/s44318-024-00193-3 (PMC11480453; doi:10.1038/s44318-024-00193-3)

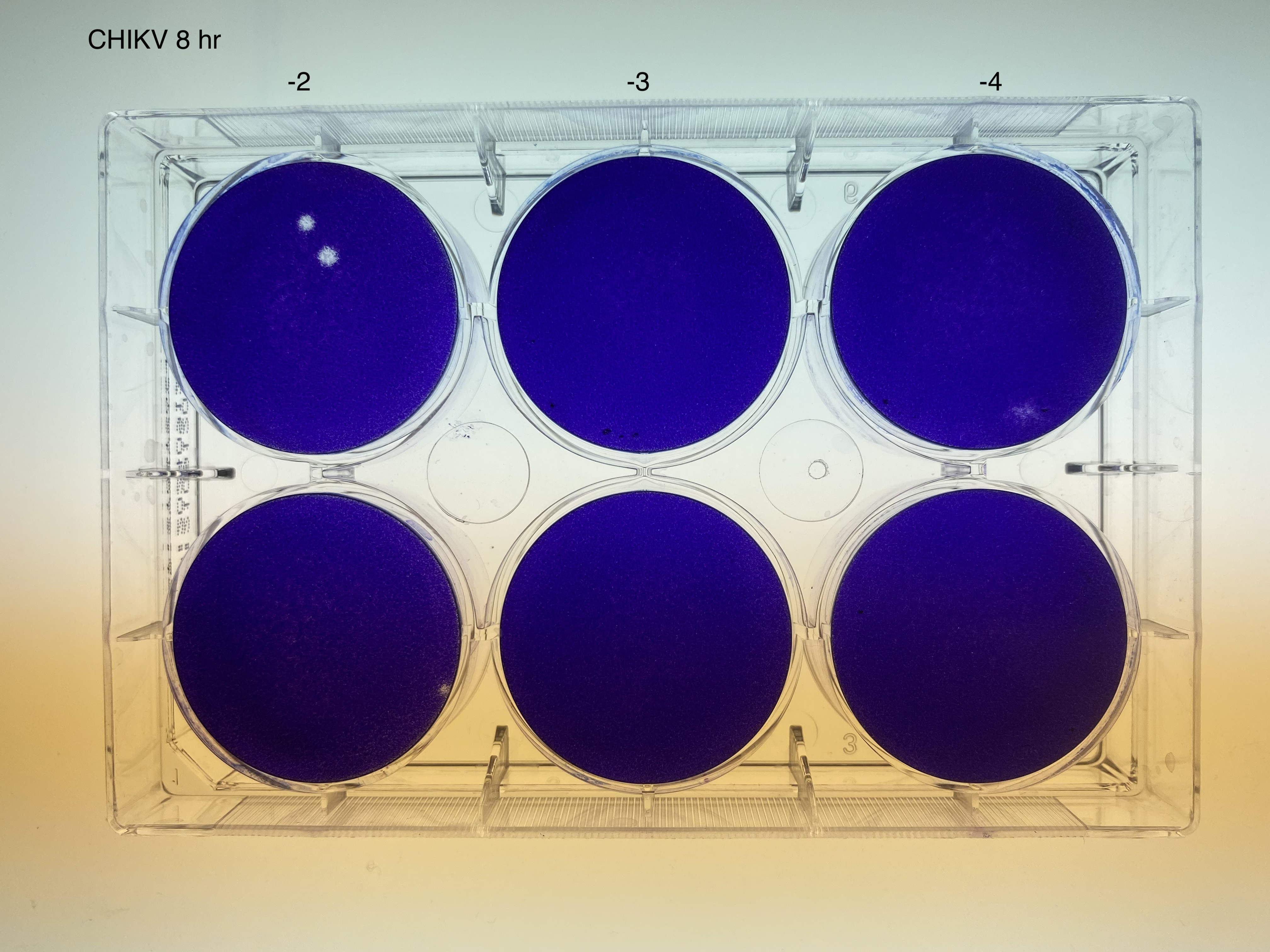

Supplement: Supplementary file 5 — Source data Fig. 1 [file 44318_2024_193_MOESM5_ESM.zip › Figure 1/1D/CHIKV 8 hr.tiff]

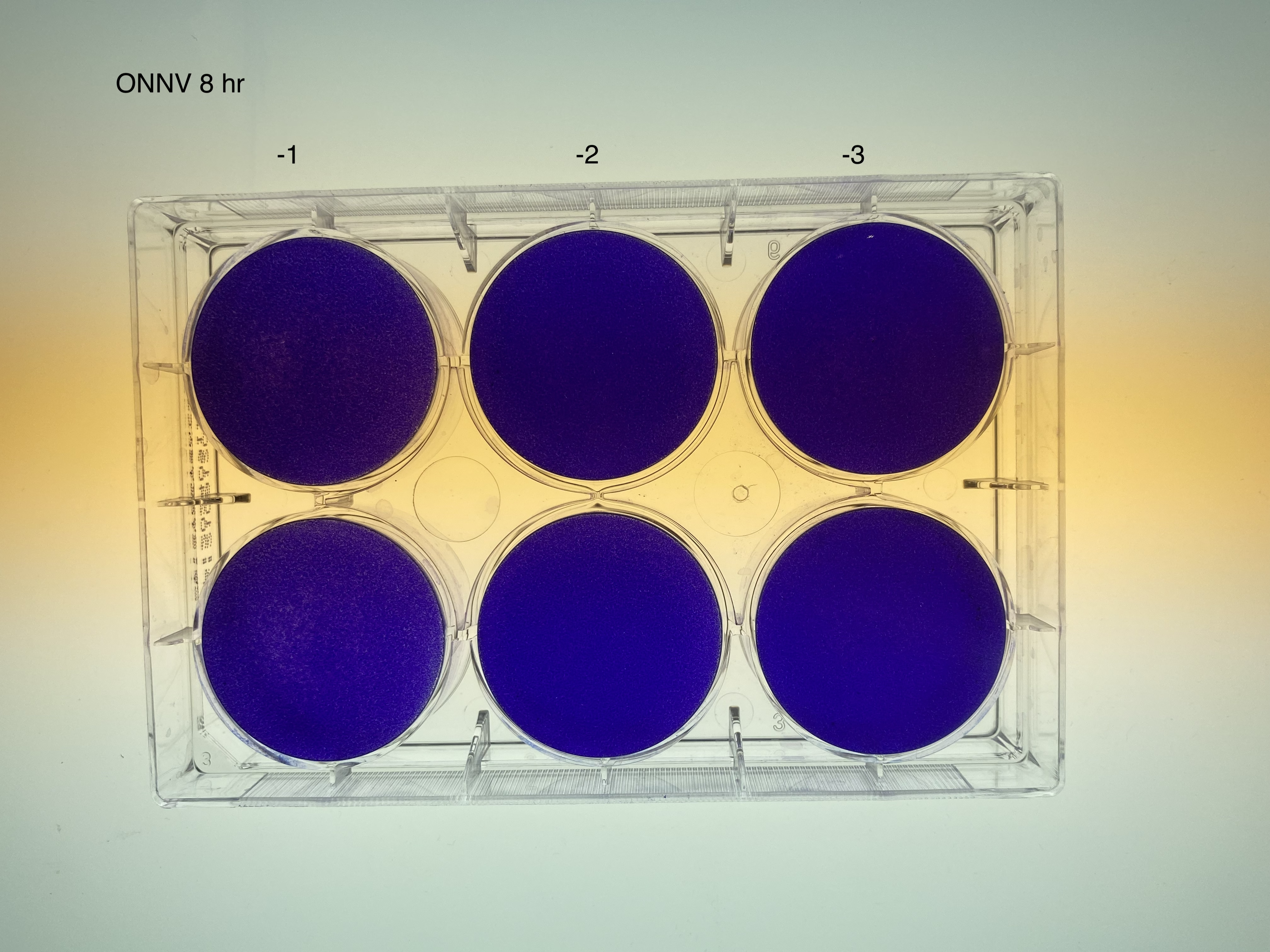

Supplement: Supplementary file 5 — Source data Fig. 1 [file 44318_2024_193_MOESM5_ESM.zip › Figure 1/1D/ONNV 8 hr.tiff]

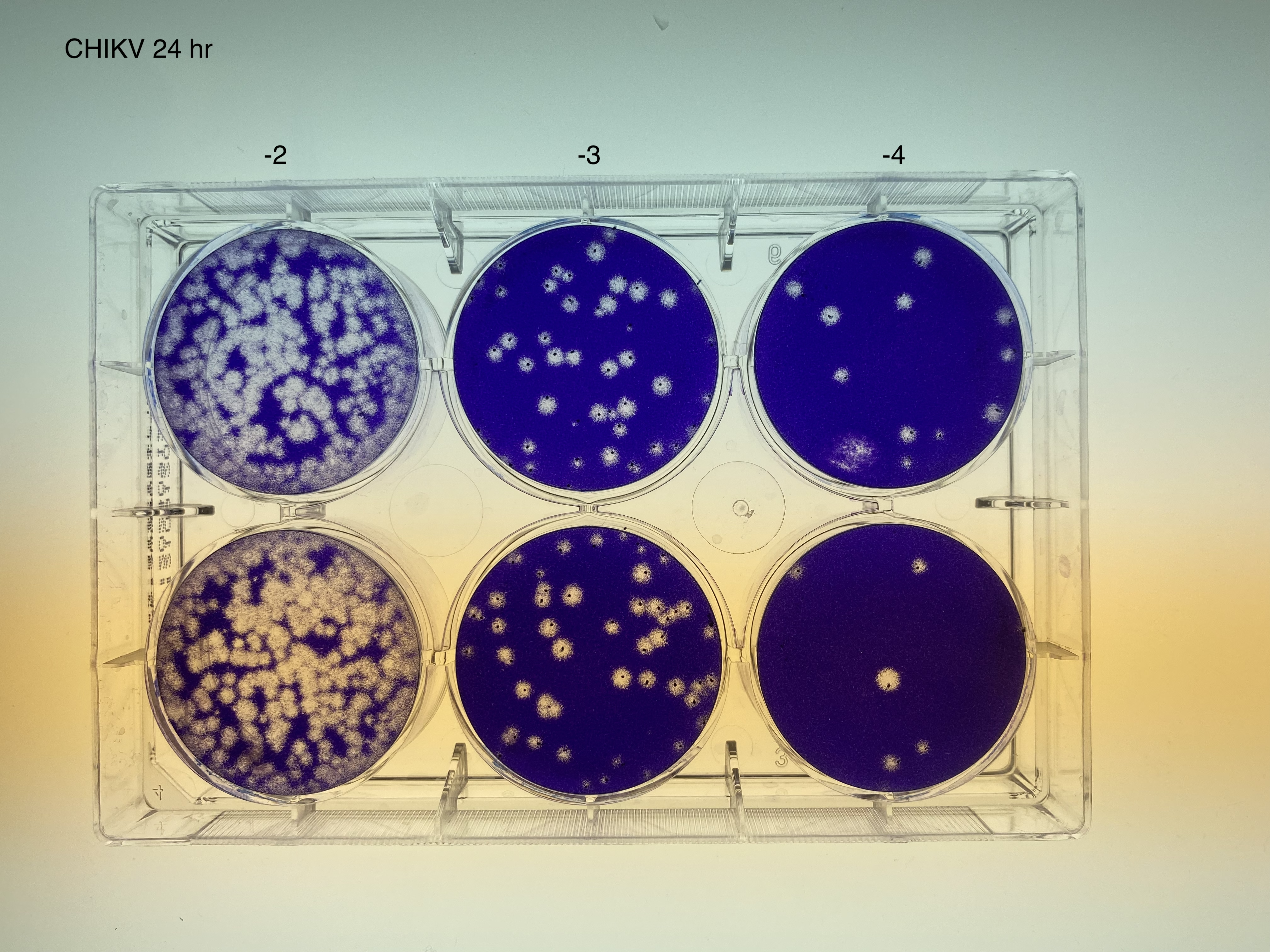

Supplement: Supplementary file 5 — Source data Fig. 1 [file 44318_2024_193_MOESM5_ESM.zip › Figure 1/1D/CHIKV 24 hr.tiff]

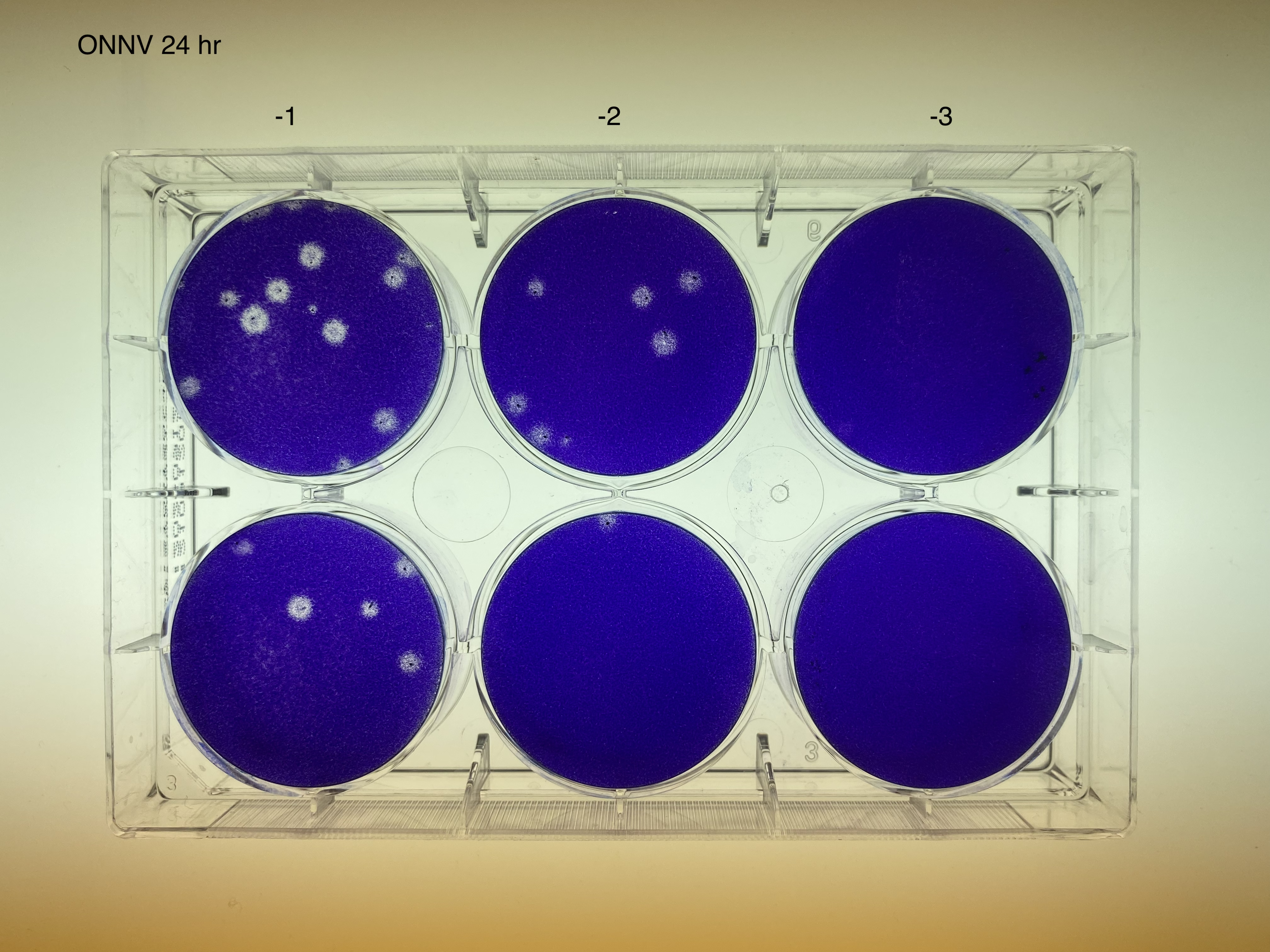

Supplement: Supplementary file 5 — Source data Fig. 1 [file 44318_2024_193_MOESM5_ESM.zip › Figure 1/1D/ONNV 24 hr.tiff]

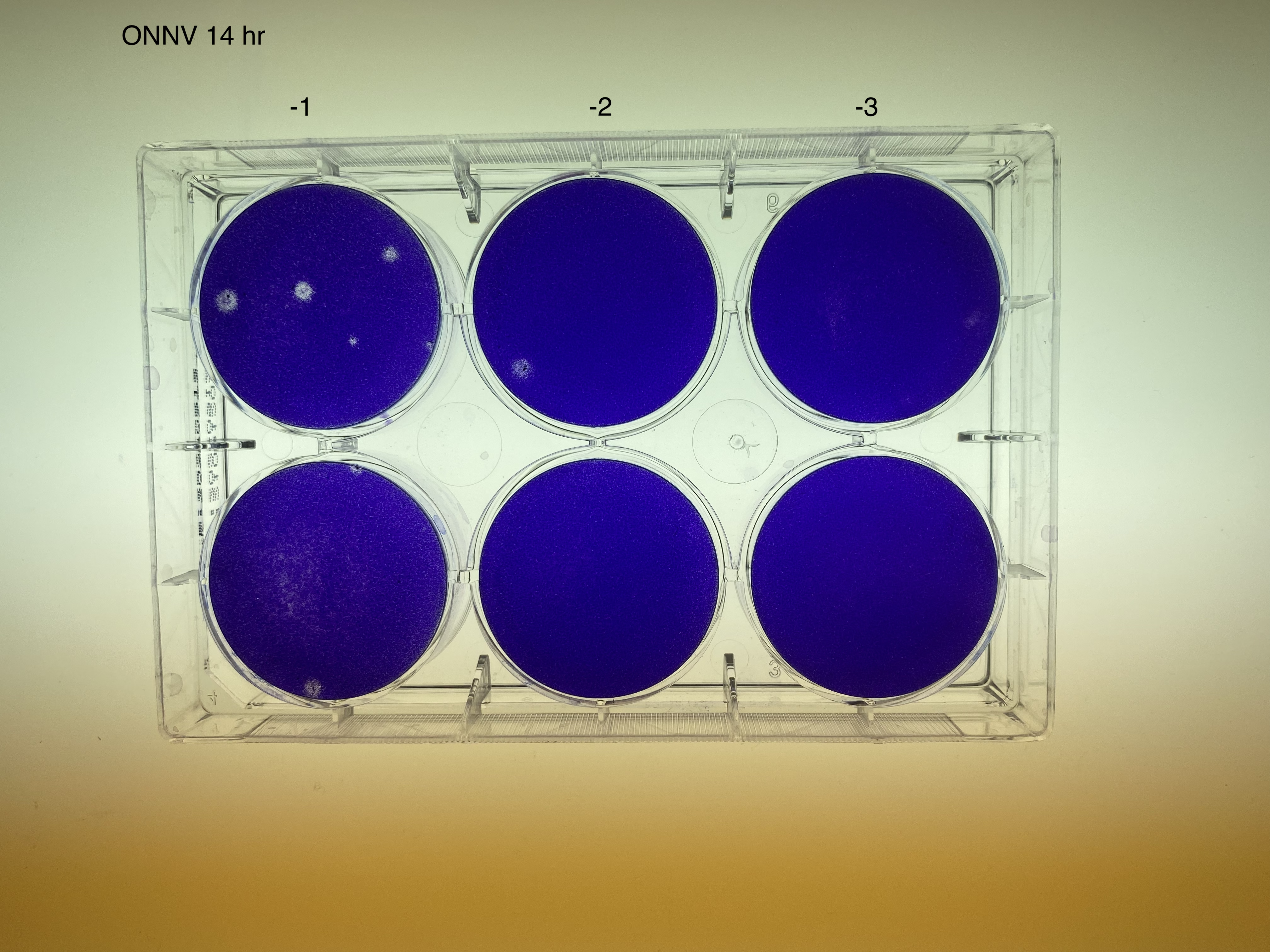

Supplement: Supplementary file 5 — Source data Fig. 1 [file 44318_2024_193_MOESM5_ESM.zip › Figure 1/1D/ONNV 14 hr.tiff]

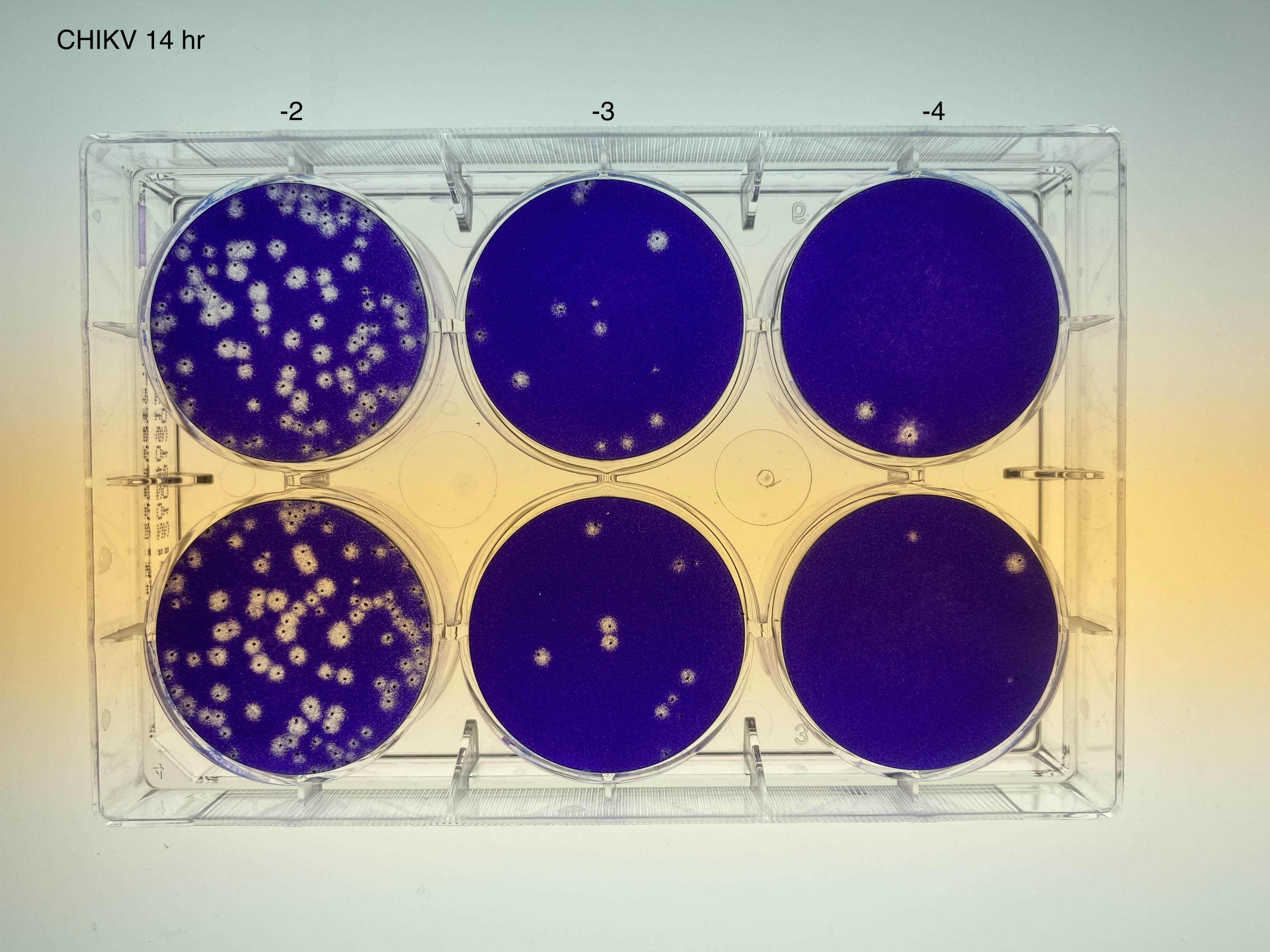

Supplement: Supplementary file 5 — Source data Fig. 1 [file 44318_2024_193_MOESM5_ESM.zip › Figure 1/1D/CHIKV 14 hr.tiff]

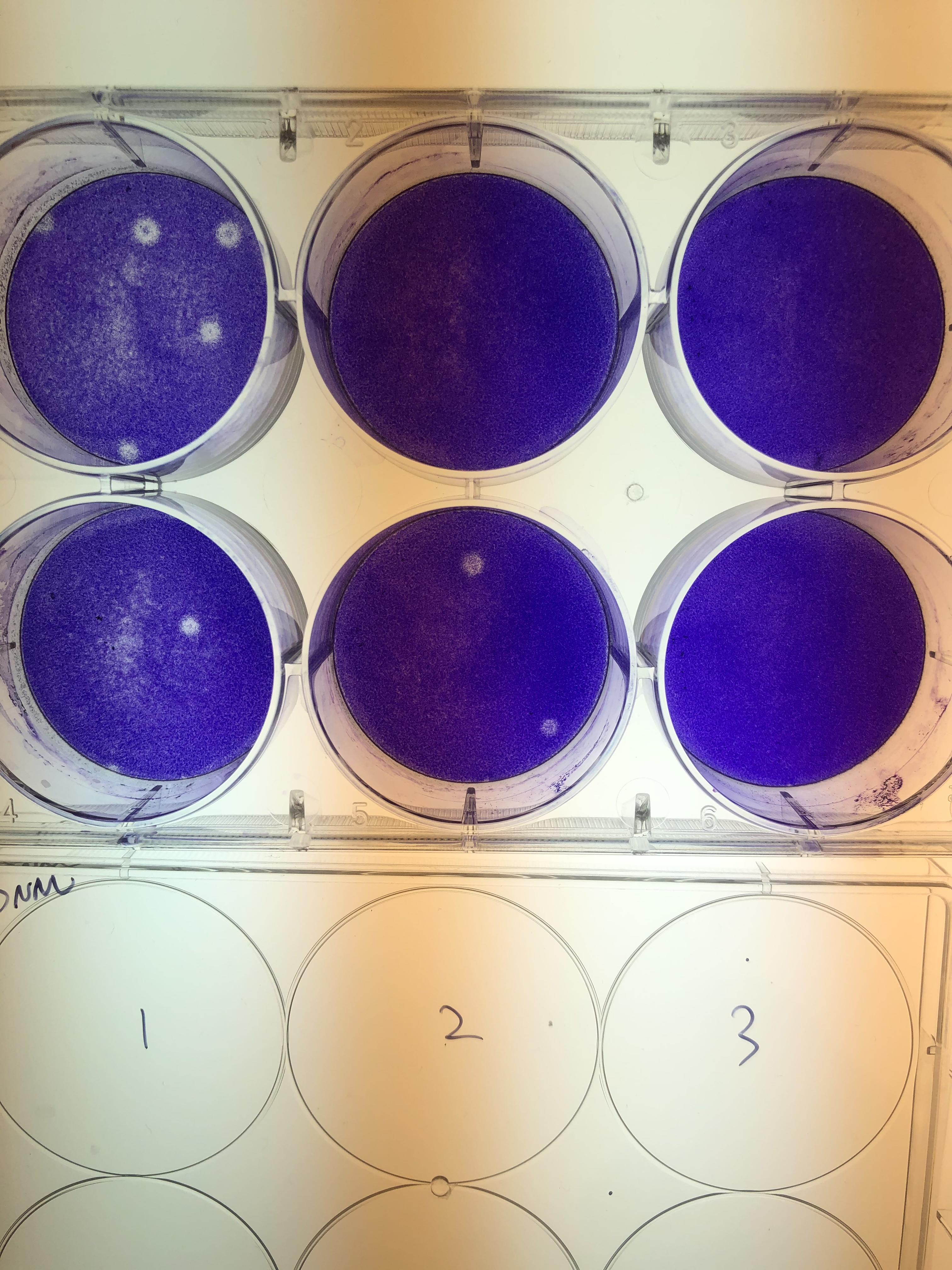

Supplement: Supplementary file 8 — Source data Fig. 4 [file 44318_2024_193_MOESM8_ESM.zip › Figure 4/4E/ONNV_ps.jpg]

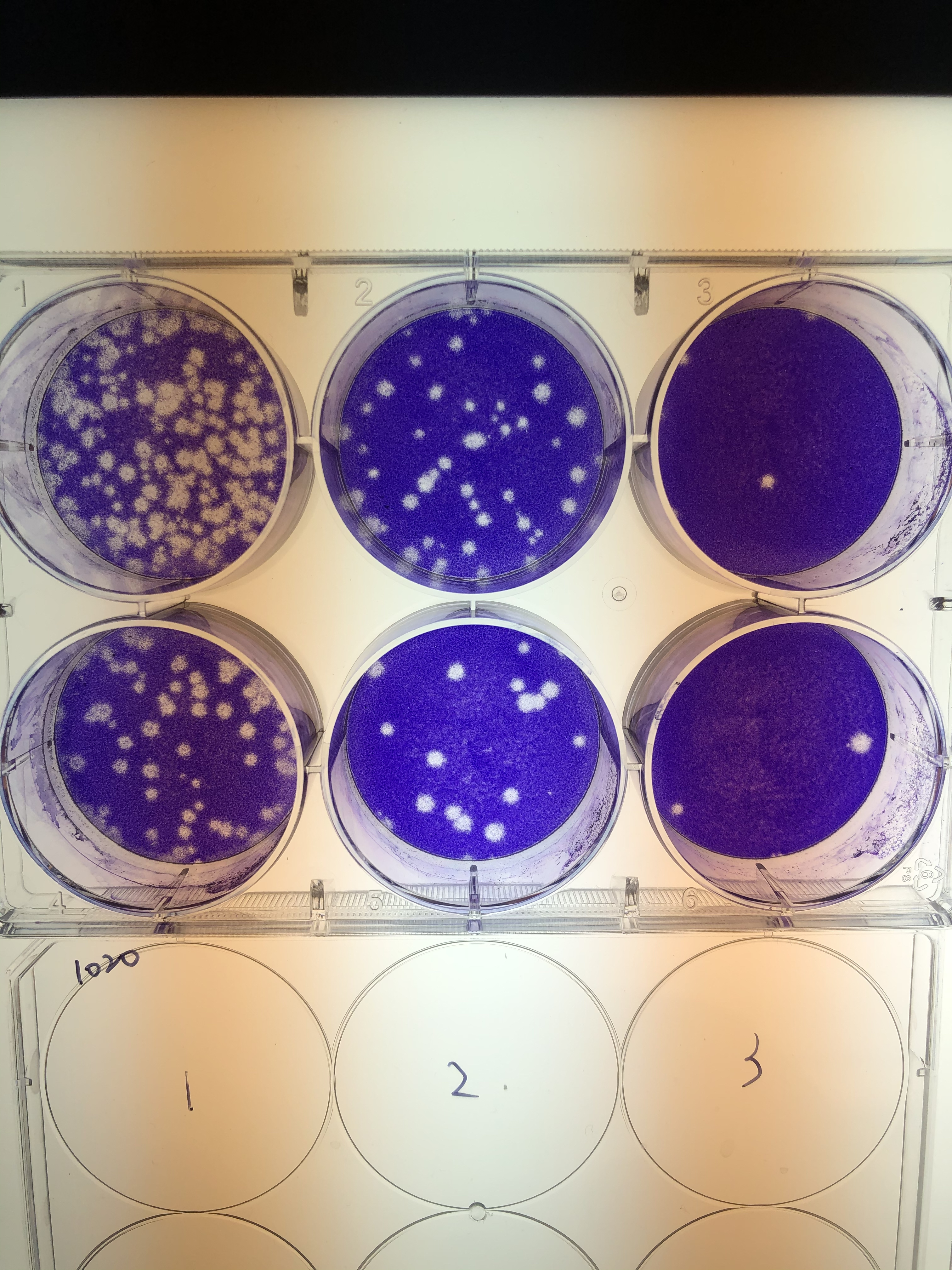

Supplement: Supplementary file 8 — Source data Fig. 4 [file 44318_2024_193_MOESM8_ESM.zip › Figure 4/4E/E1-E211K.jpg]

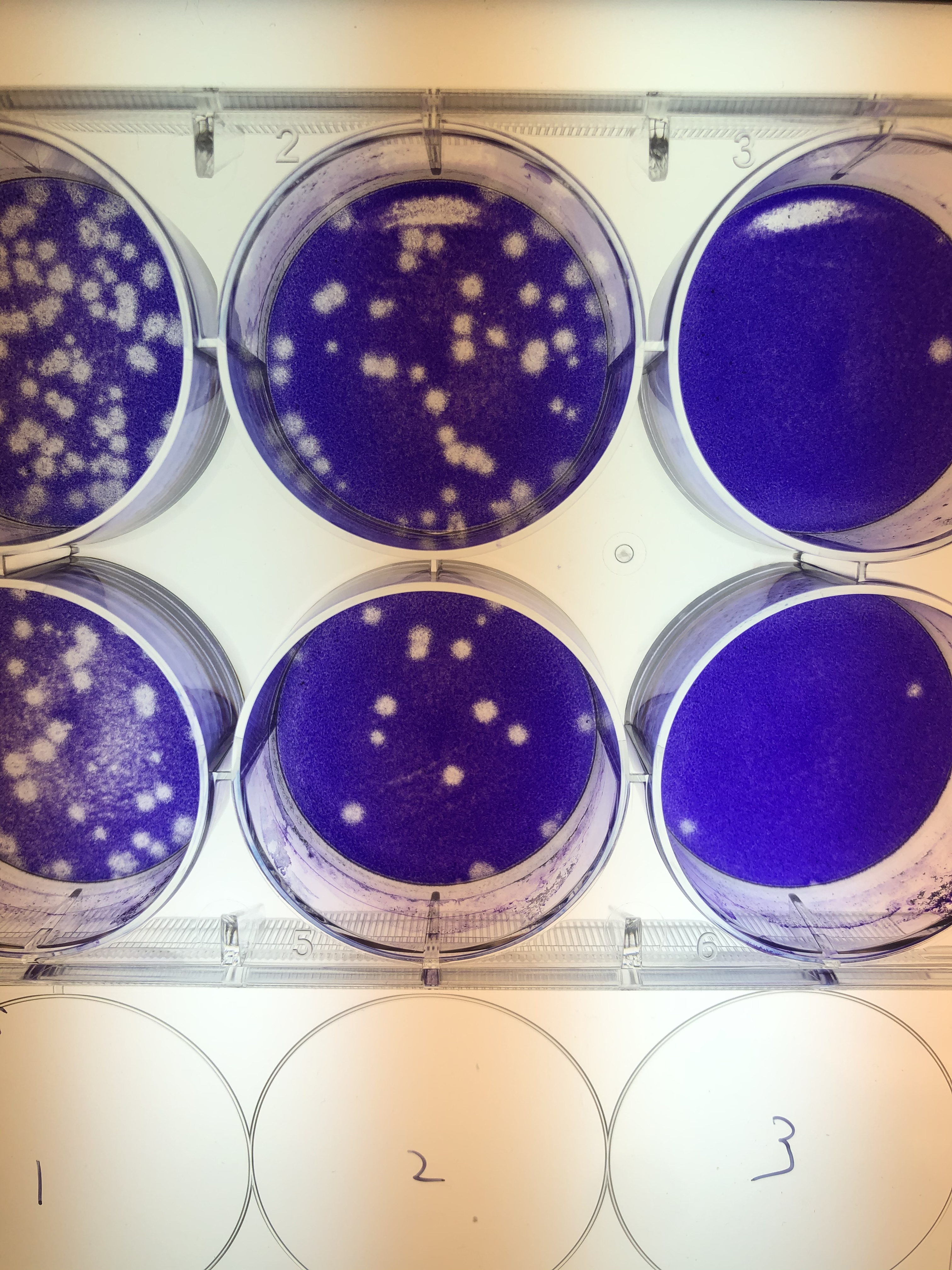

Supplement: Supplementary file 8 — Source data Fig. 4 [file 44318_2024_193_MOESM8_ESM.zip › Figure 4/4E/E1-R366K.jpg]

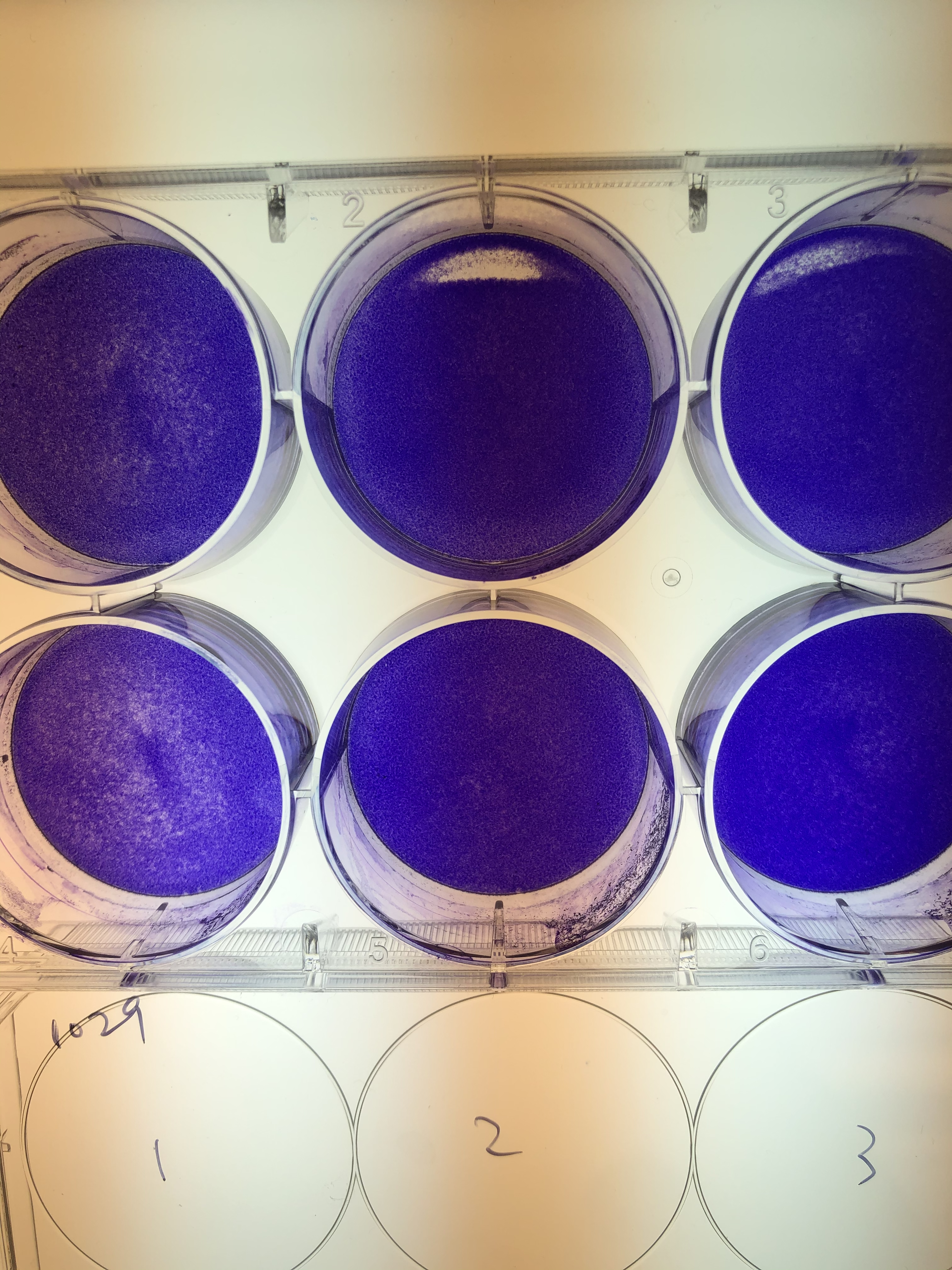

Supplement: Supplementary file 8 — Source data Fig. 4 [file 44318_2024_193_MOESM8_ESM.zip › Figure 4/4E/E1-V220I.jpg]

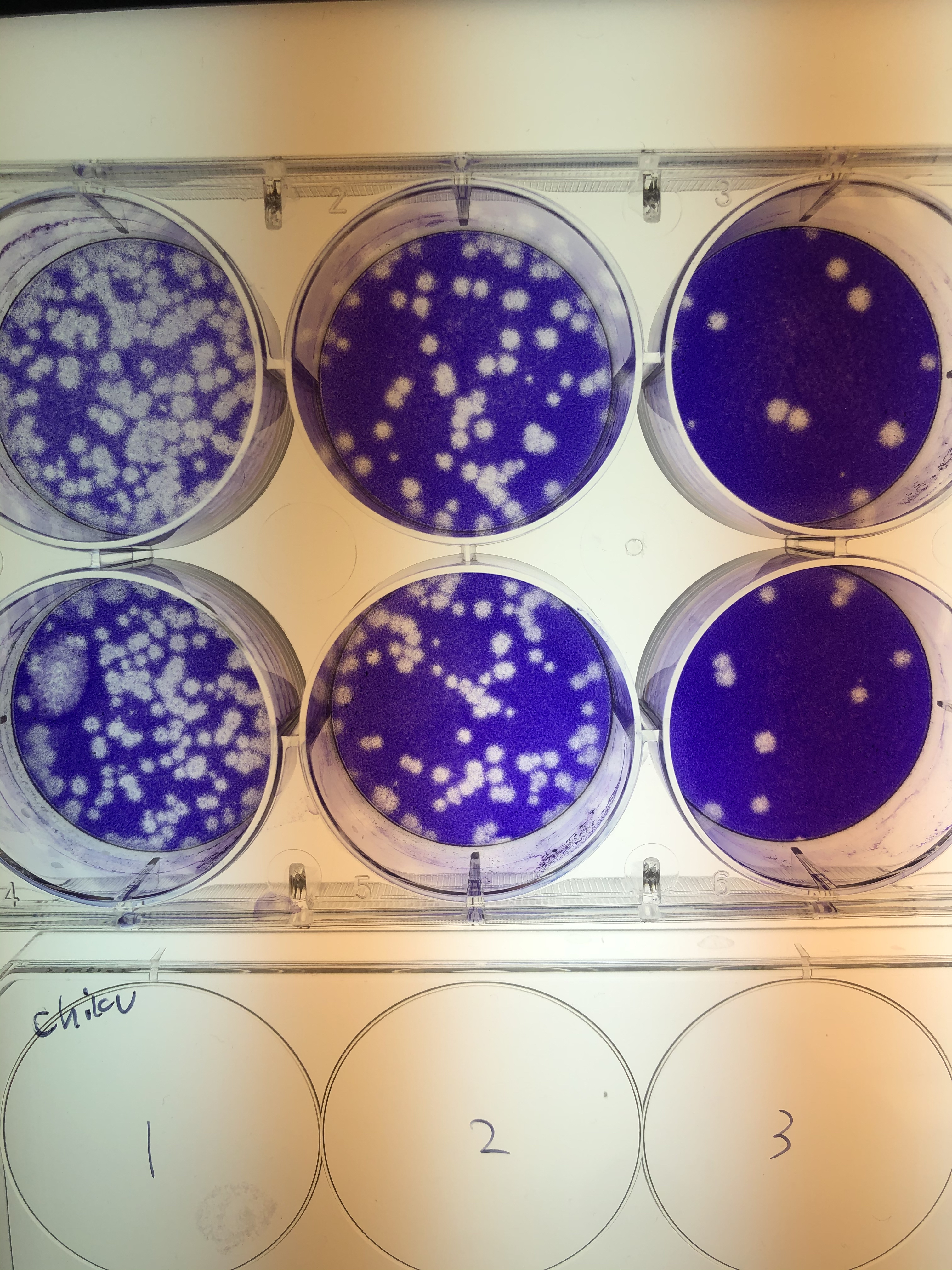

Supplement: Supplementary file 8 — Source data Fig. 4 [file 44318_2024_193_MOESM8_ESM.zip › Figure 4/4E/CHIKV_ps.jpg]

## Slide 1
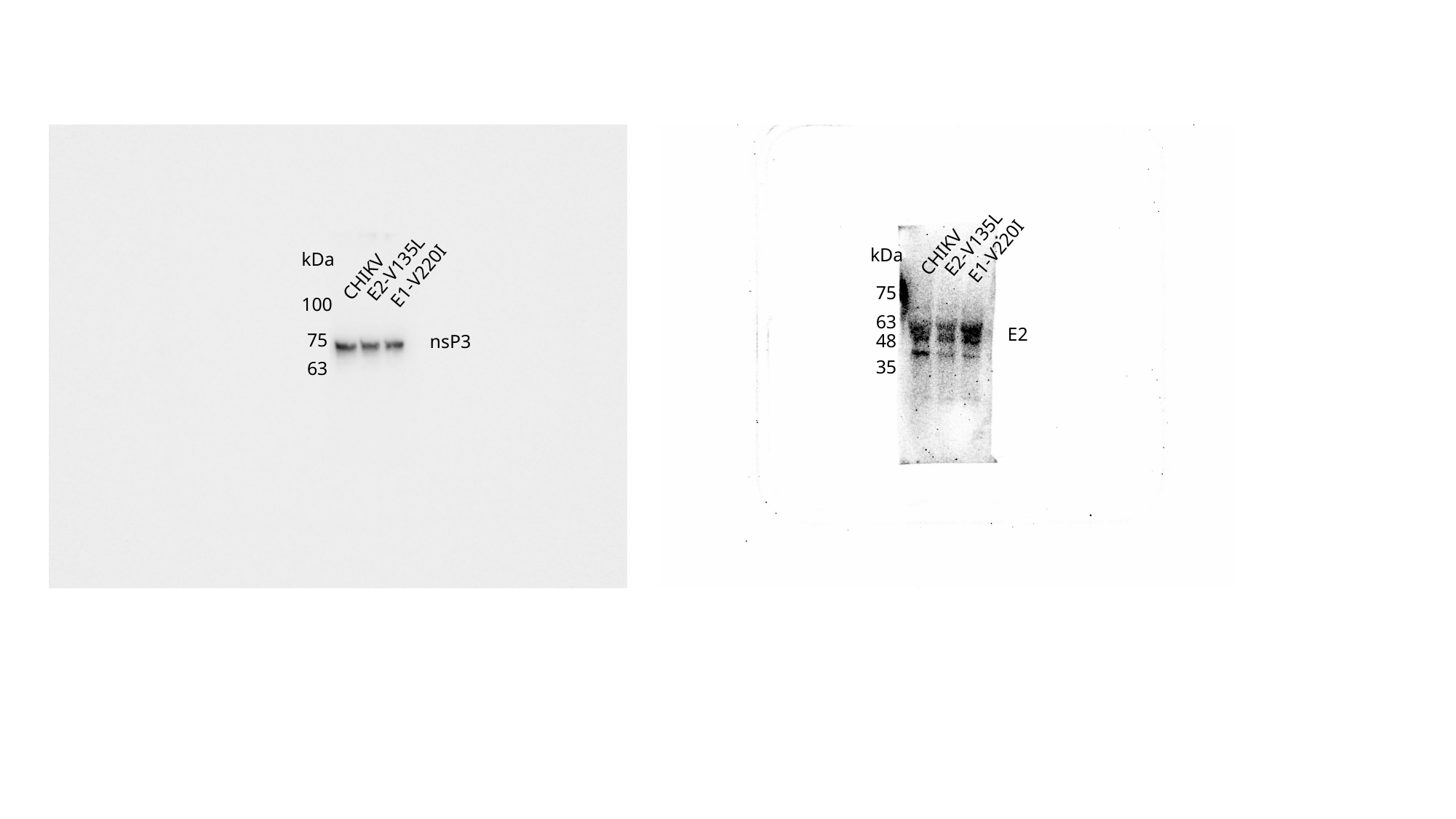

E2-V135L
E1-V220I
CHIKV
kDa
E2-V135L
kDa
E1-V220I
CHIKV
75
100
63
E2
75
48
nsP3
35
63

## Slide 2
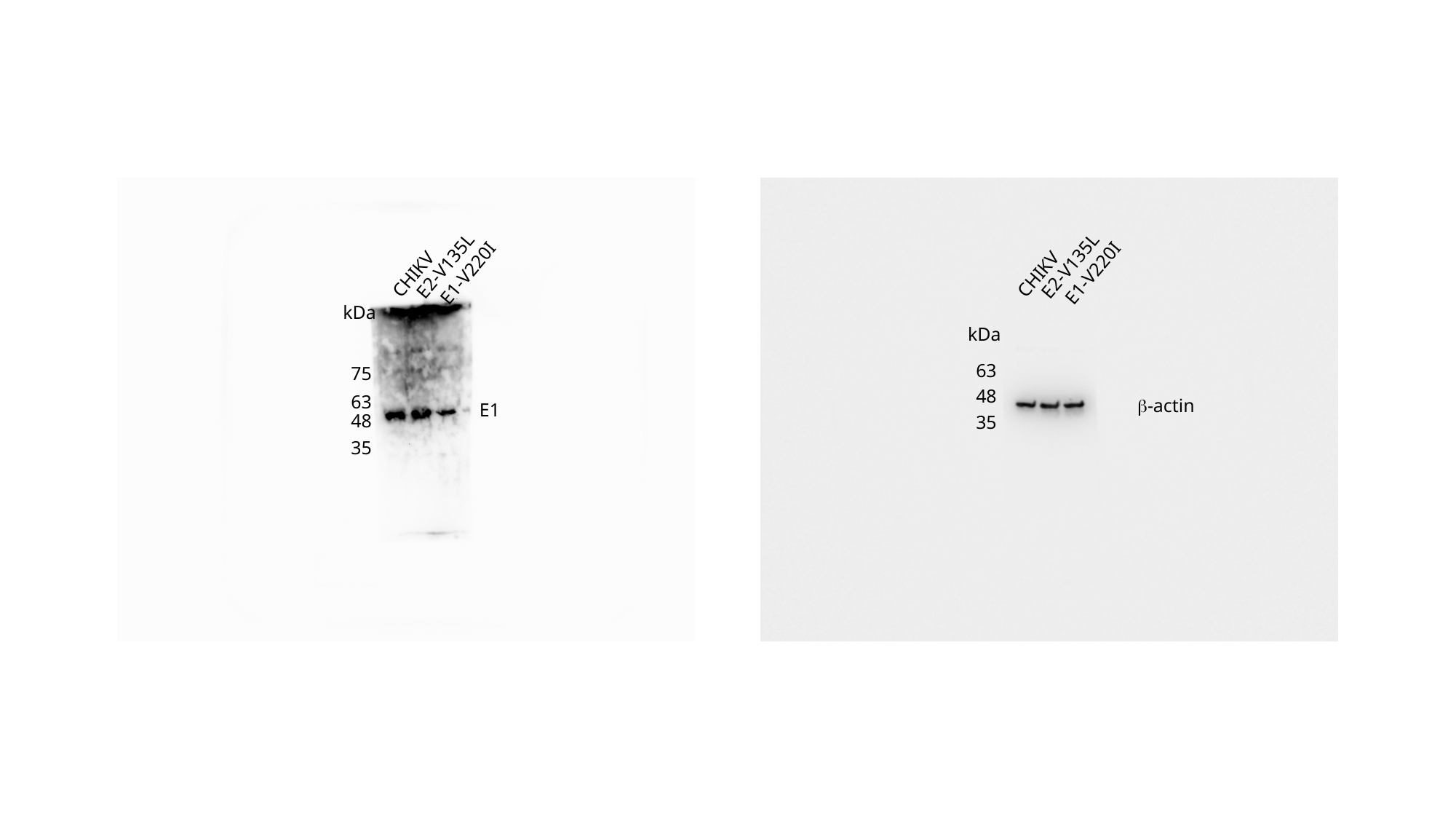

E2-V135L
E2-V135L
E1-V220I
E1-V220I
CHIKV
CHIKV
kDa
kDa
63
75
48
63
b-actin
E1
48
35
35

Supplement: Supplementary file 8 — Source data Fig. 4 [file 44318_2024_193_MOESM8_ESM.zip › Figure 4/4F/4F WB images.pptx]

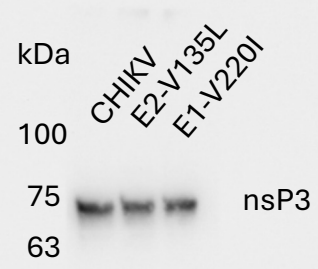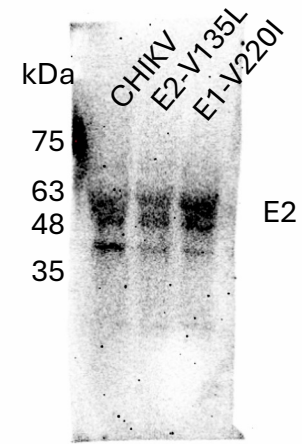

CHIKV  
E2-V135L  
E1-V220I

kDa

75

63

48

35

E1

CHIKV  
E2-V135L  
E1-V220I

kDa

63

48

35

$\beta$ -actin

Supplement: Supplementary file 8 — Source data Fig. 4 [file 44318_2024_193_MOESM8_ESM.zip › Figure 4/4F/4F WB images.pdf]

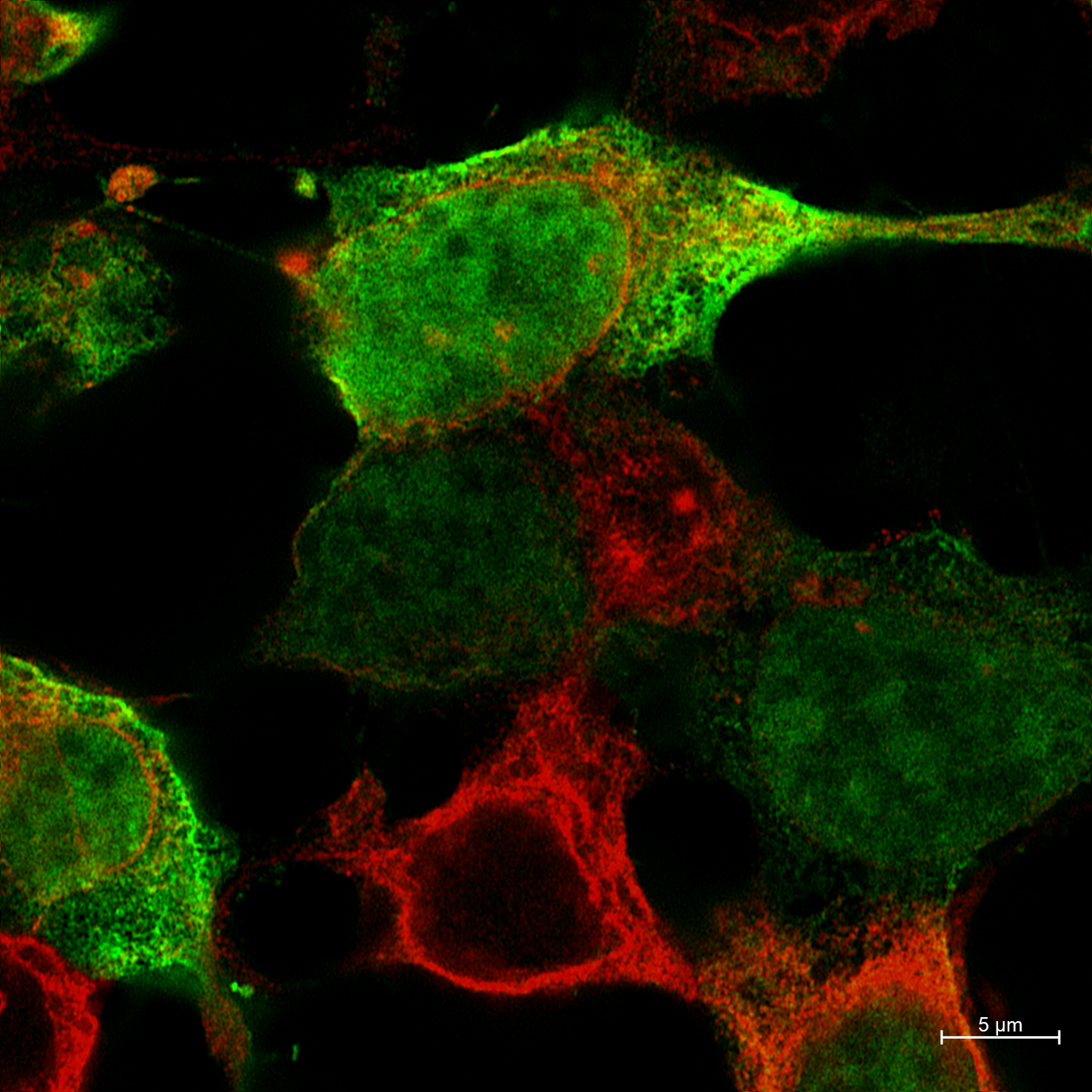

Supplement: Supplementary file 10 — Source data Fig. 7 [file 44318_2024_193_MOESM10_ESM.zip › Figure 7/7B/7B images/7B E1 vs eIF3k.tif]

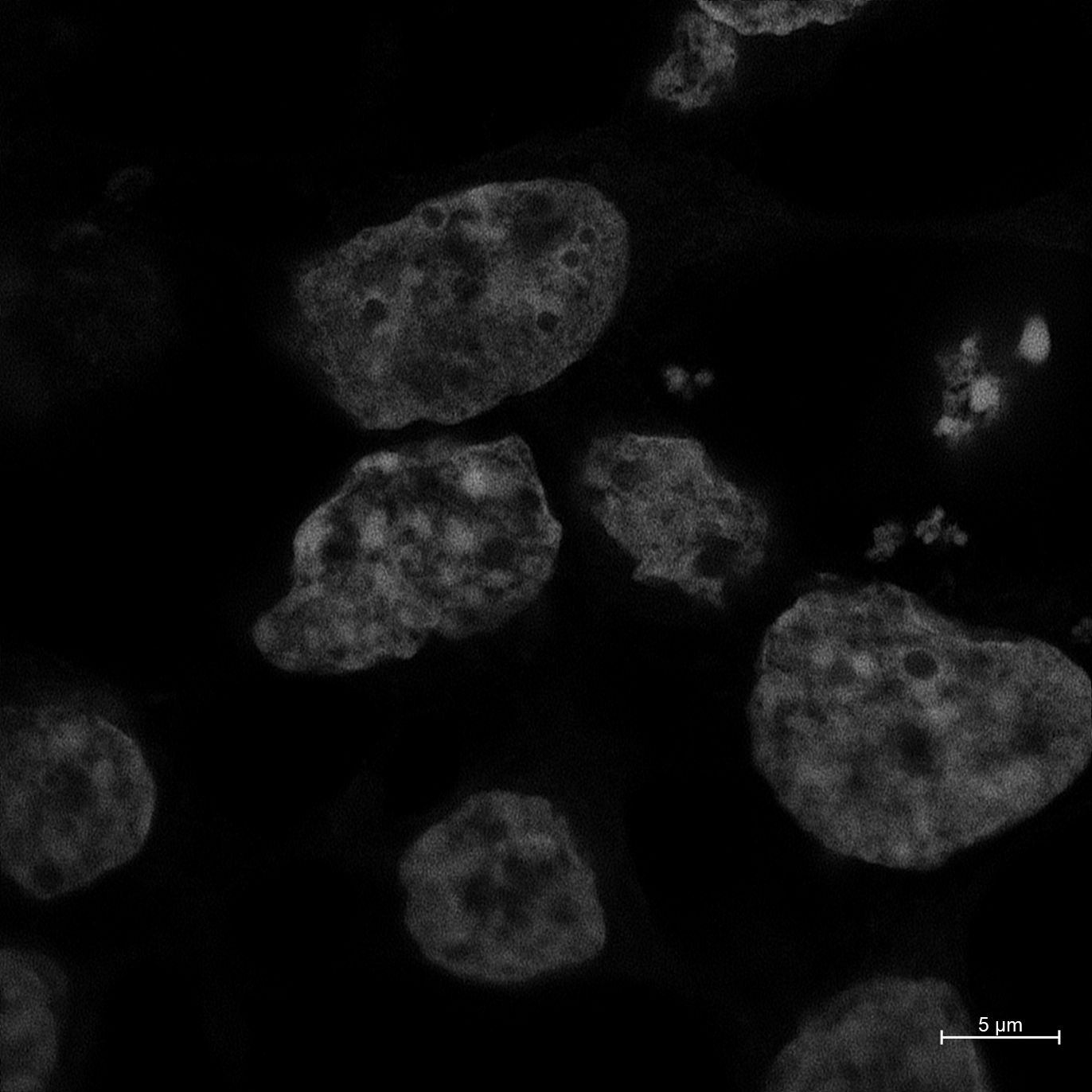

Supplement: Supplementary file 10 — Source data Fig. 7 [file 44318_2024_193_MOESM10_ESM.zip › Figure 7/7B/7B images/7B DAPI.tif]

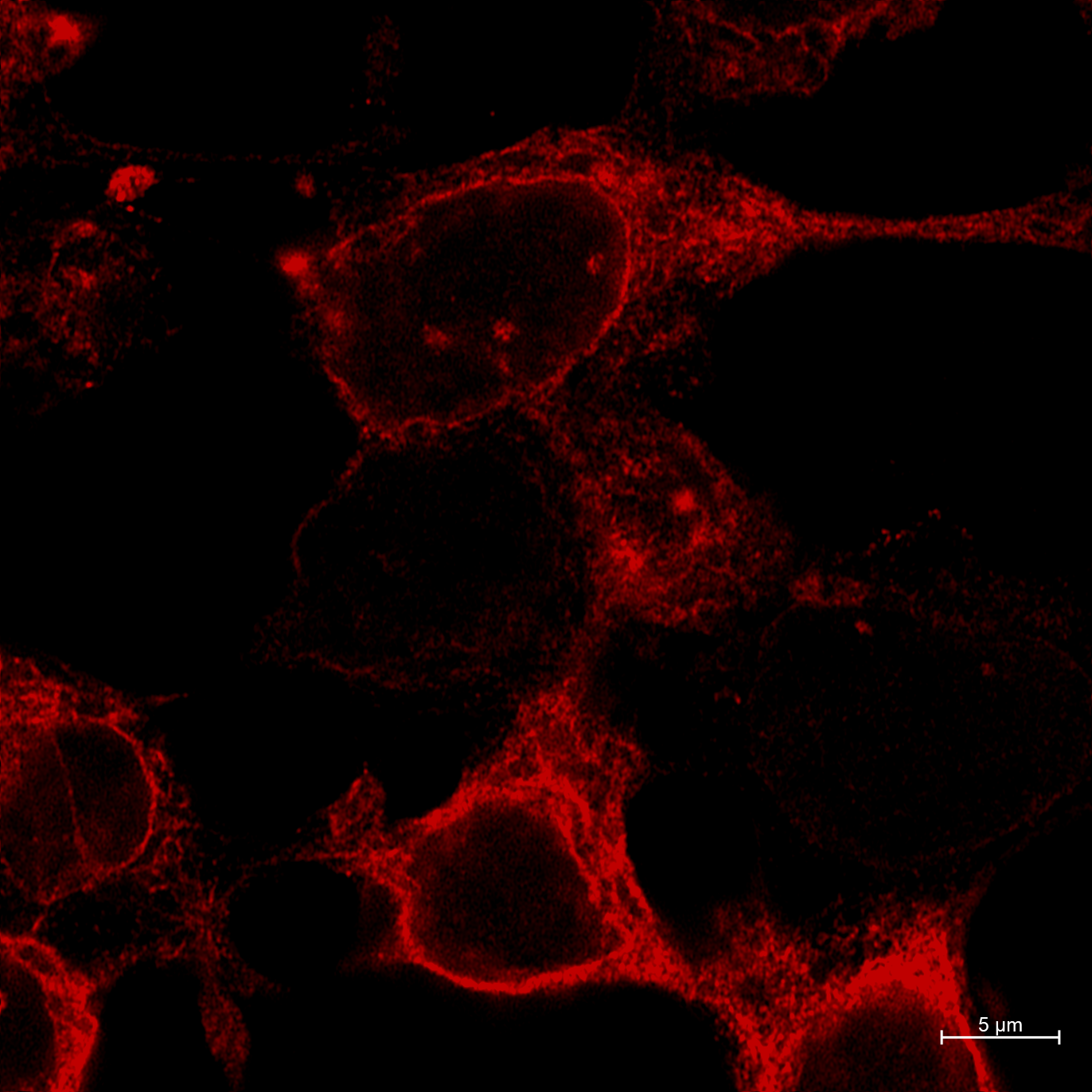

Supplement: Supplementary file 10 — Source data Fig. 7 [file 44318_2024_193_MOESM10_ESM.zip › Figure 7/7B/7B images/7B E1.tif]

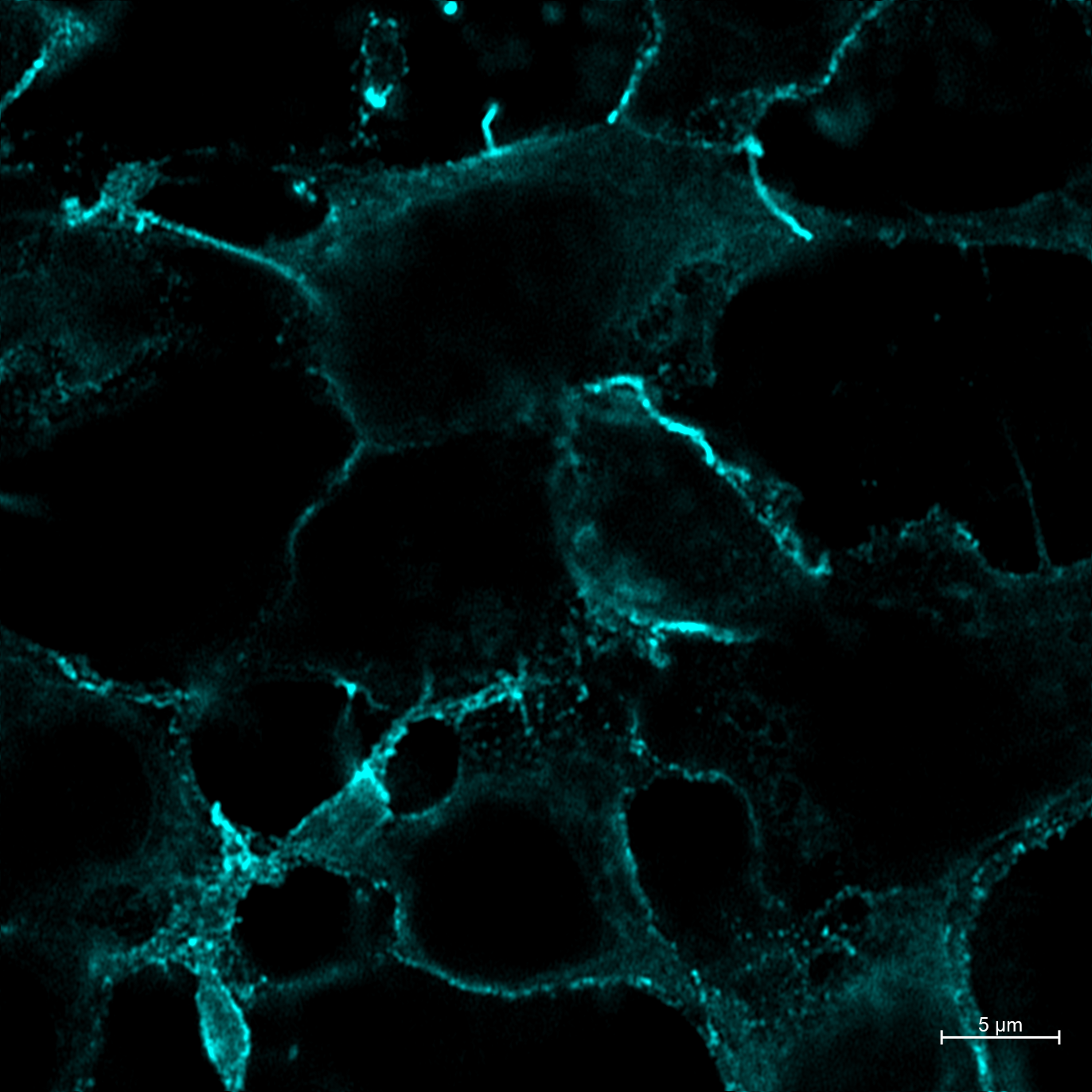

Supplement: Supplementary file 10 — Source data Fig. 7 [file 44318_2024_193_MOESM10_ESM.zip › Figure 7/7B/7B images/7B E2.tif]

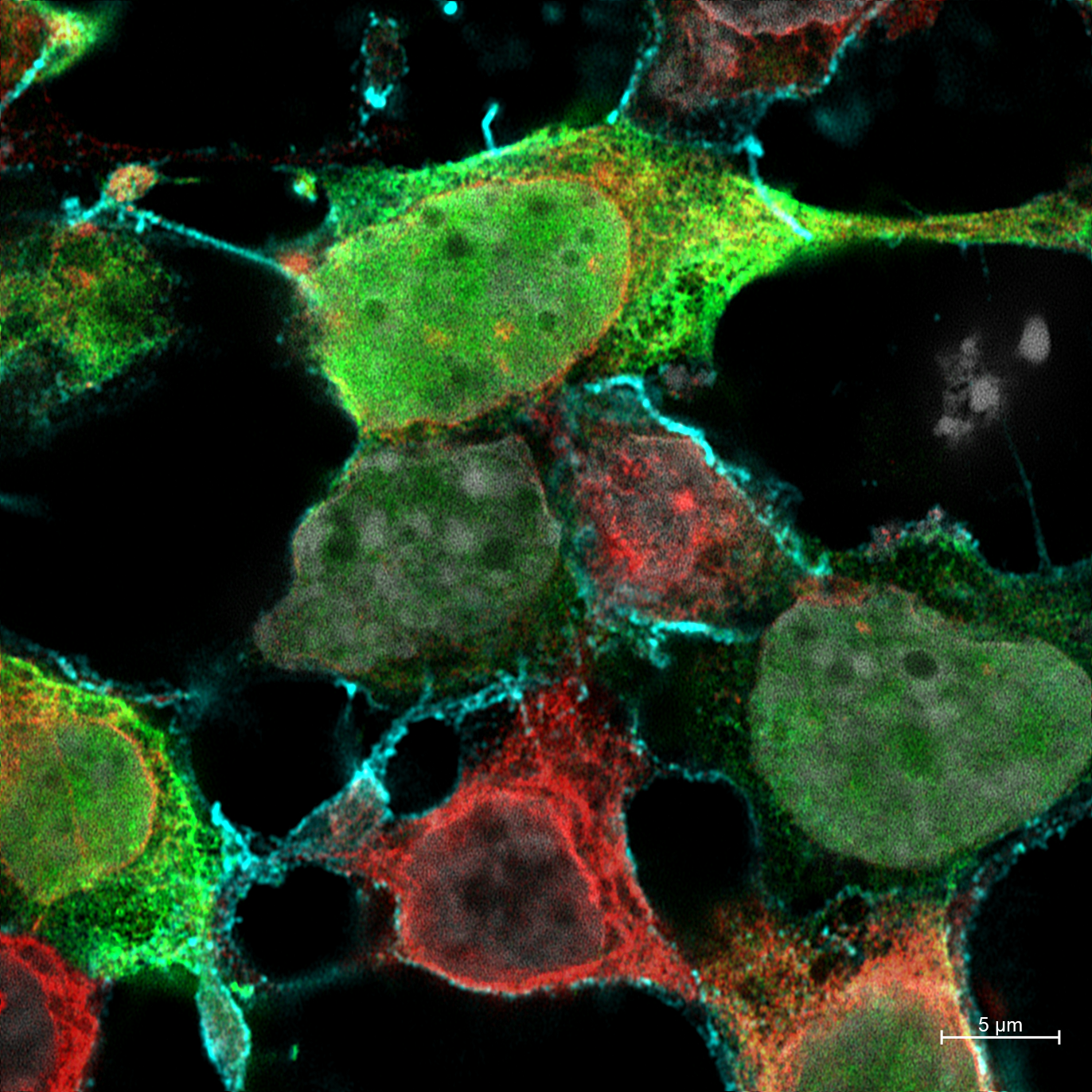

Supplement: Supplementary file 10 — Source data Fig. 7 [file 44318_2024_193_MOESM10_ESM.zip › Figure 7/7B/7B images/7B merged.tif]

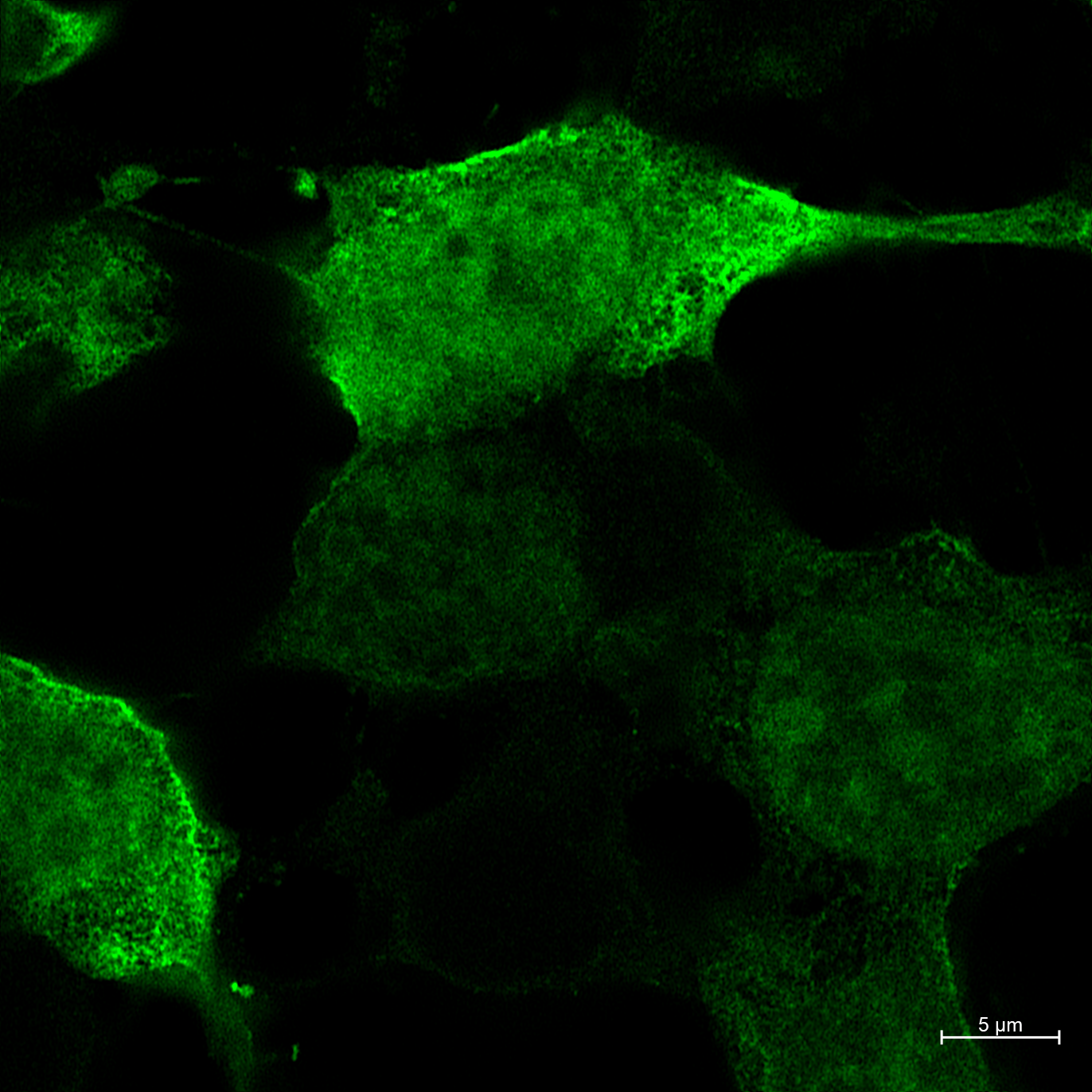

Supplement: Supplementary file 10 — Source data Fig. 7 [file 44318_2024_193_MOESM10_ESM.zip › Figure 7/7B/7B images/7B eIF3k.tif]

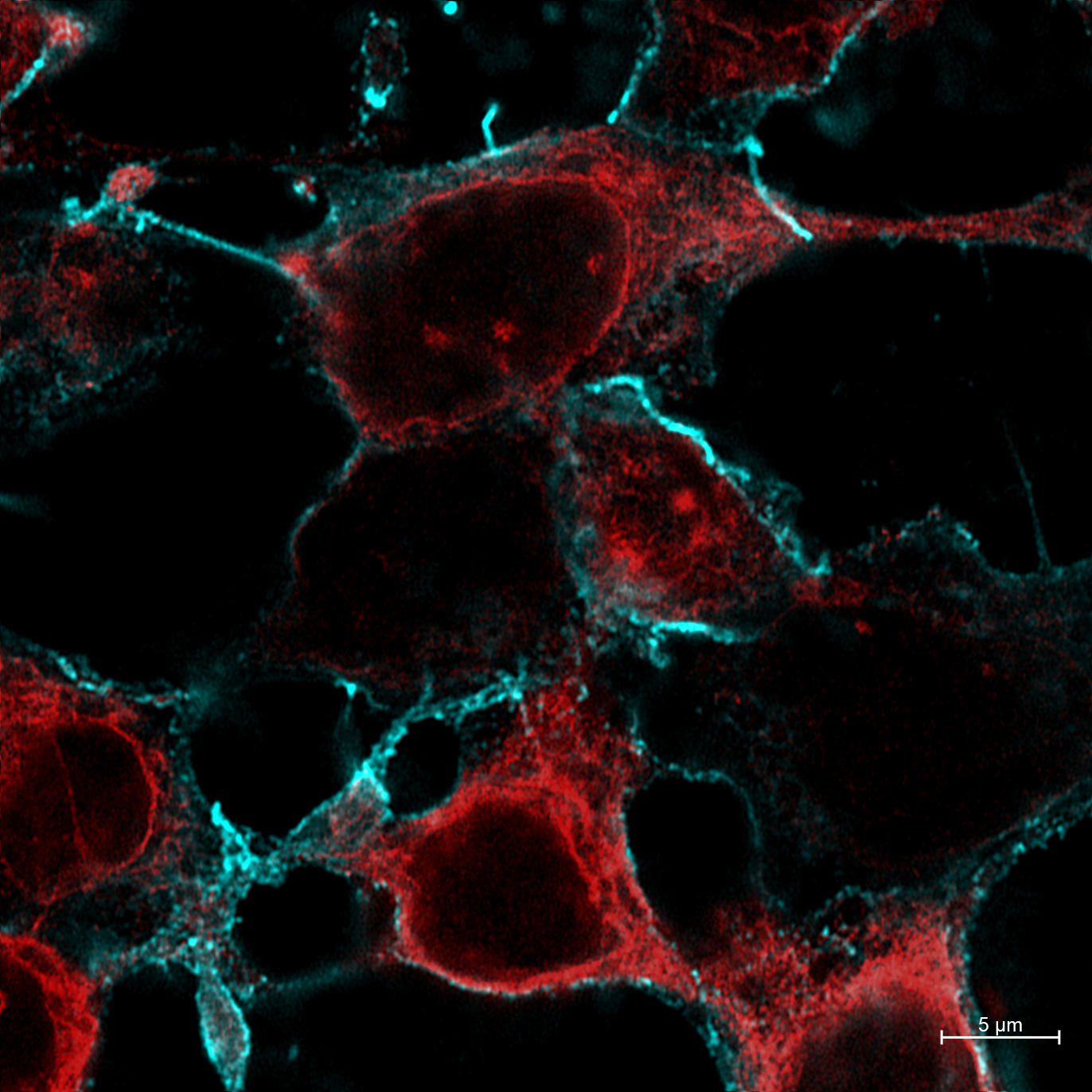

Supplement: Supplementary file 10 — Source data Fig. 7 [file 44318_2024_193_MOESM10_ESM.zip › Figure 7/7B/7B images/7B E1 vs E2.tif]

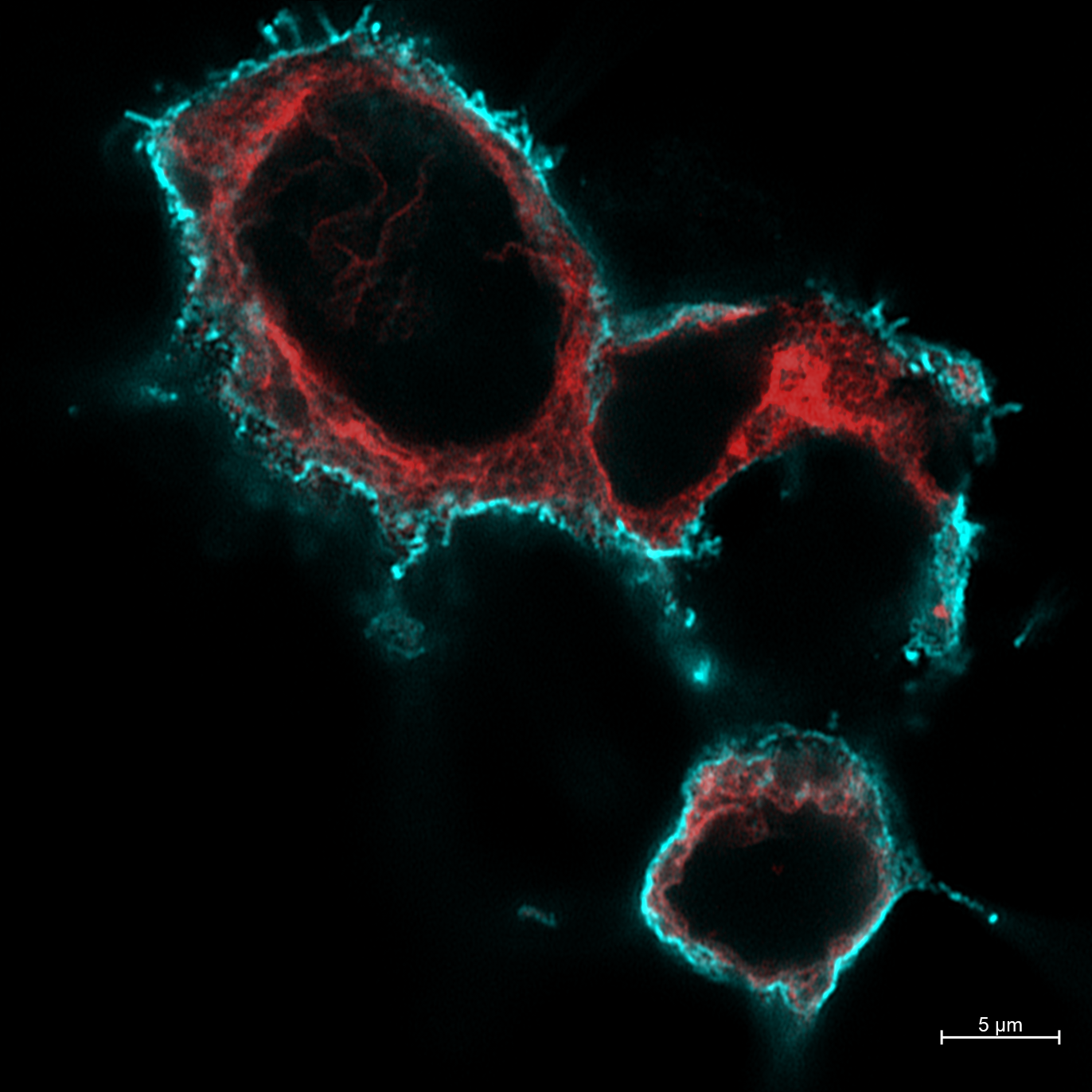

Supplement: Supplementary file 10 — Source data Fig. 7 [file 44318_2024_193_MOESM10_ESM.zip › Figure 7/7A/7A images/7A E1 vs E2.tif]

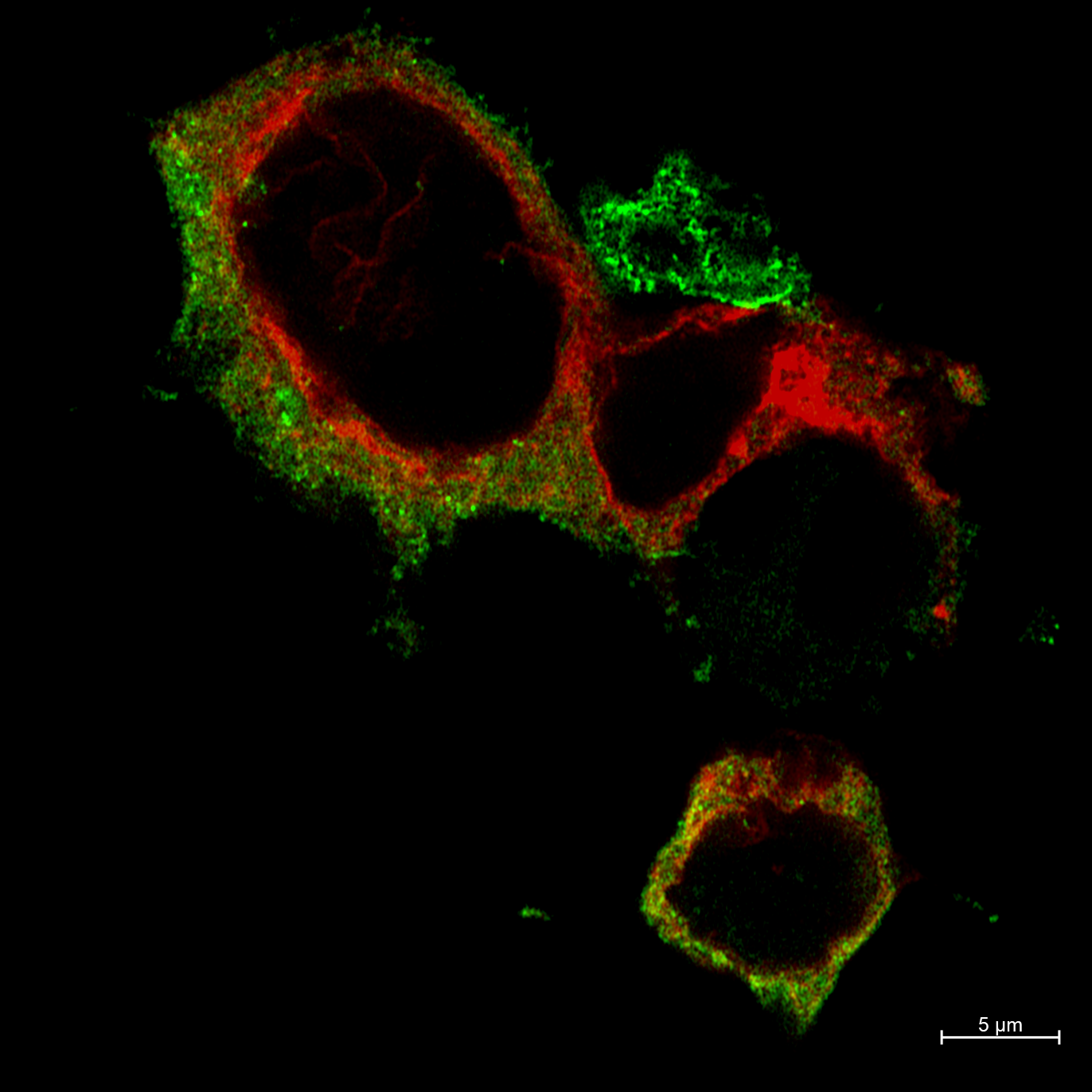

Supplement: Supplementary file 10 — Source data Fig. 7 [file 44318_2024_193_MOESM10_ESM.zip › Figure 7/7A/7A images/7A E1 vs SPCS3.tif]

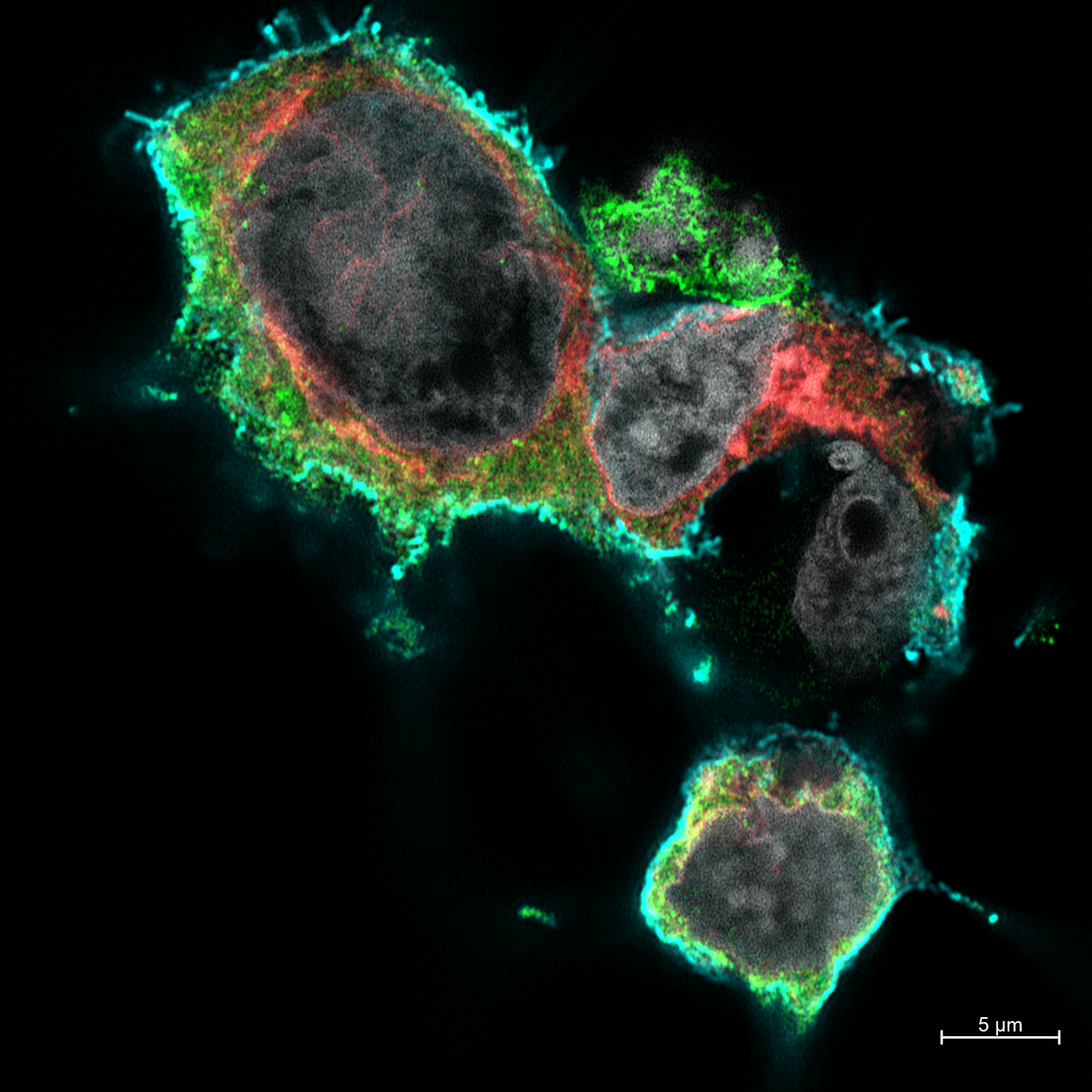

Supplement: Supplementary file 10 — Source data Fig. 7 [file 44318_2024_193_MOESM10_ESM.zip › Figure 7/7A/7A images/7A merged.tif]

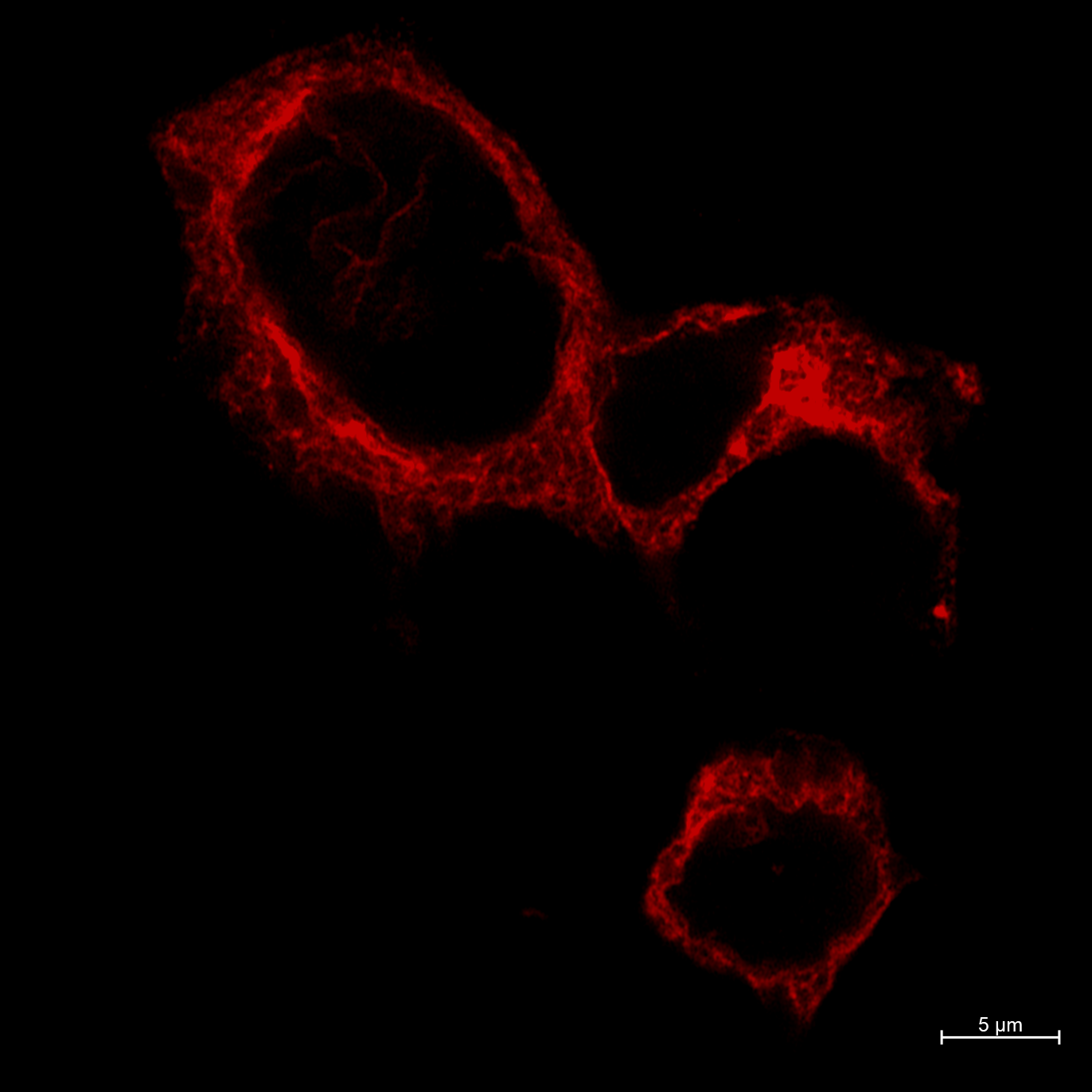

Supplement: Supplementary file 10 — Source data Fig. 7 [file 44318_2024_193_MOESM10_ESM.zip › Figure 7/7A/7A images/7A E1.tif]

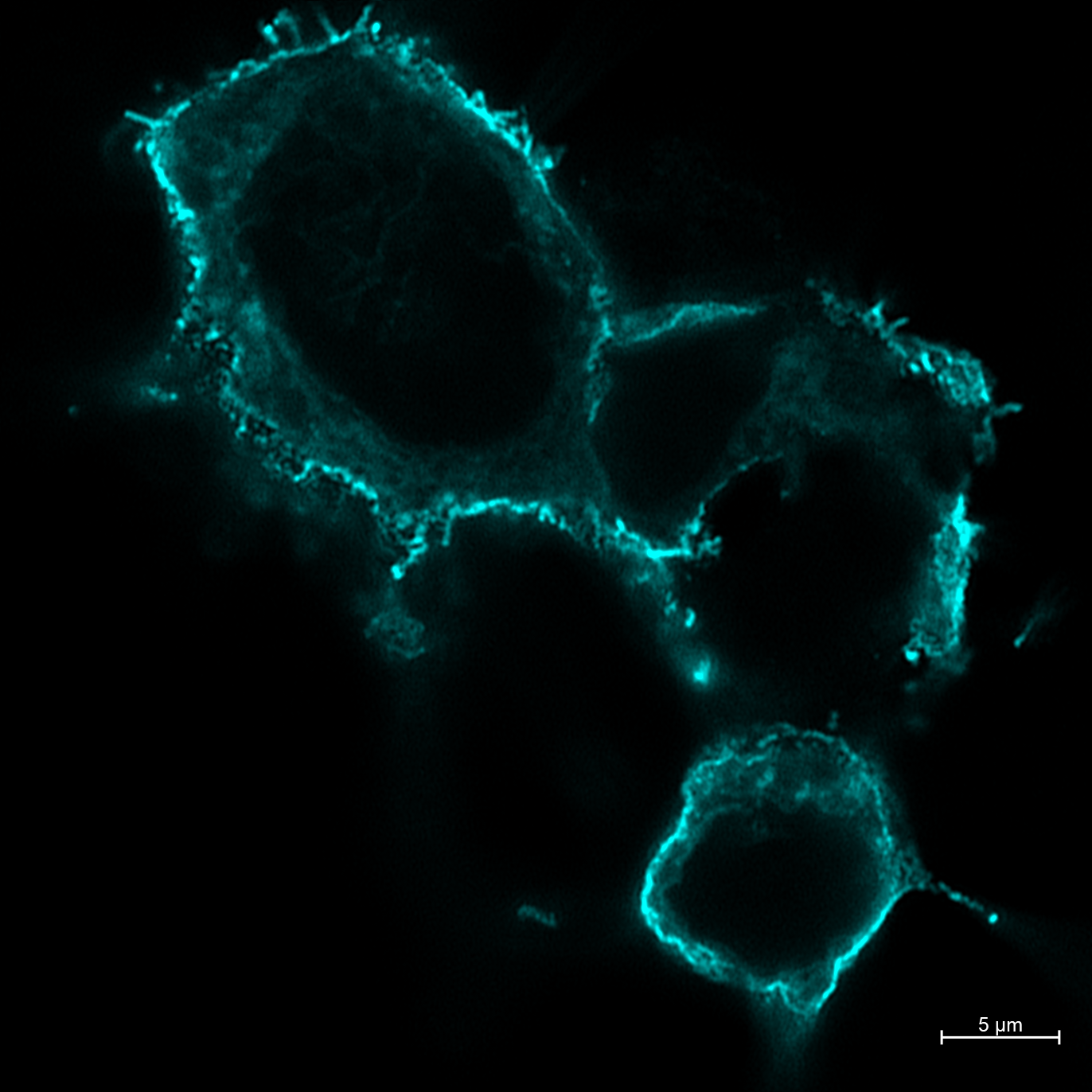

Supplement: Supplementary file 10 — Source data Fig. 7 [file 44318_2024_193_MOESM10_ESM.zip › Figure 7/7A/7A images/7A E2.tif]

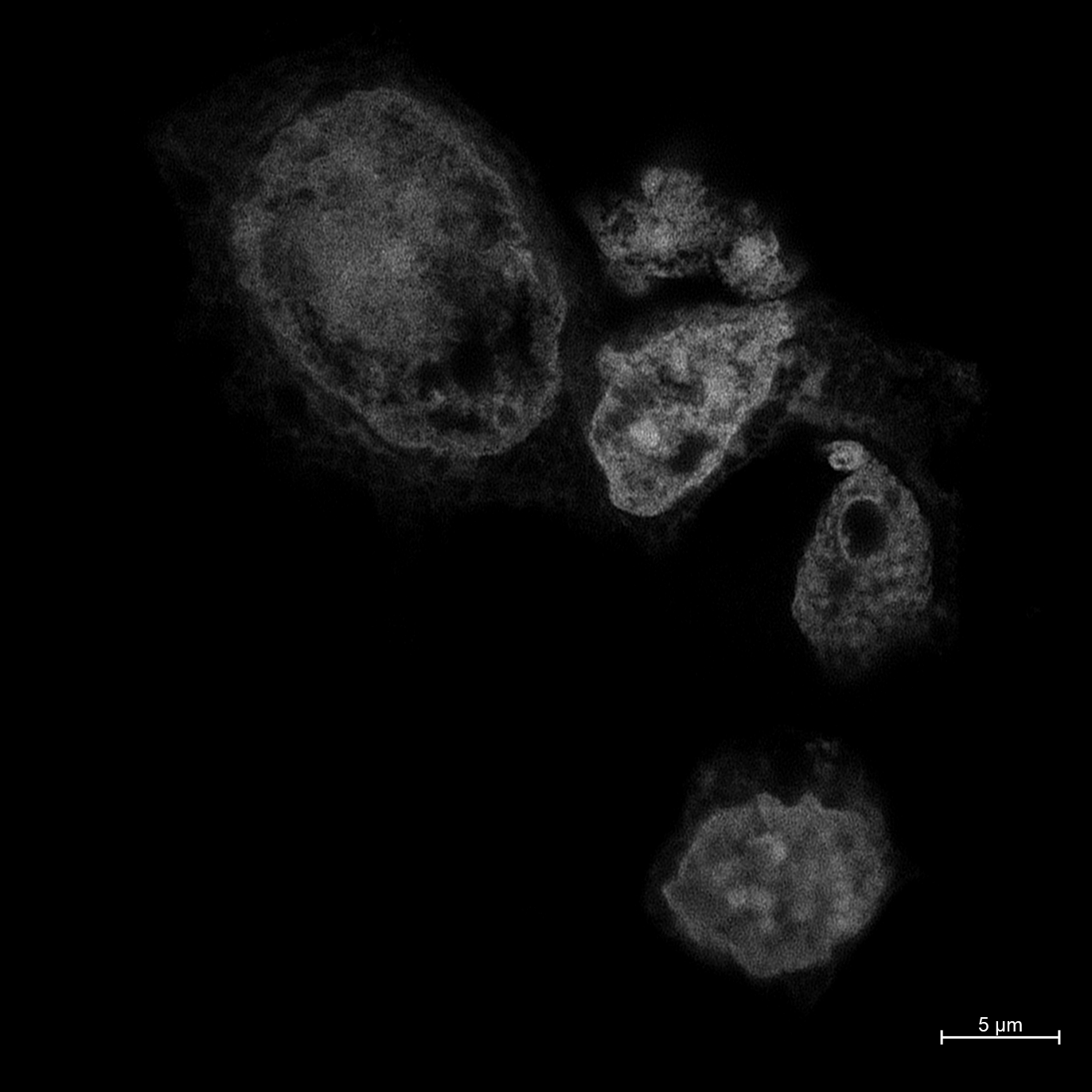

Supplement: Supplementary file 10 — Source data Fig. 7 [file 44318_2024_193_MOESM10_ESM.zip › Figure 7/7A/7A images/7A DAPI.tif]

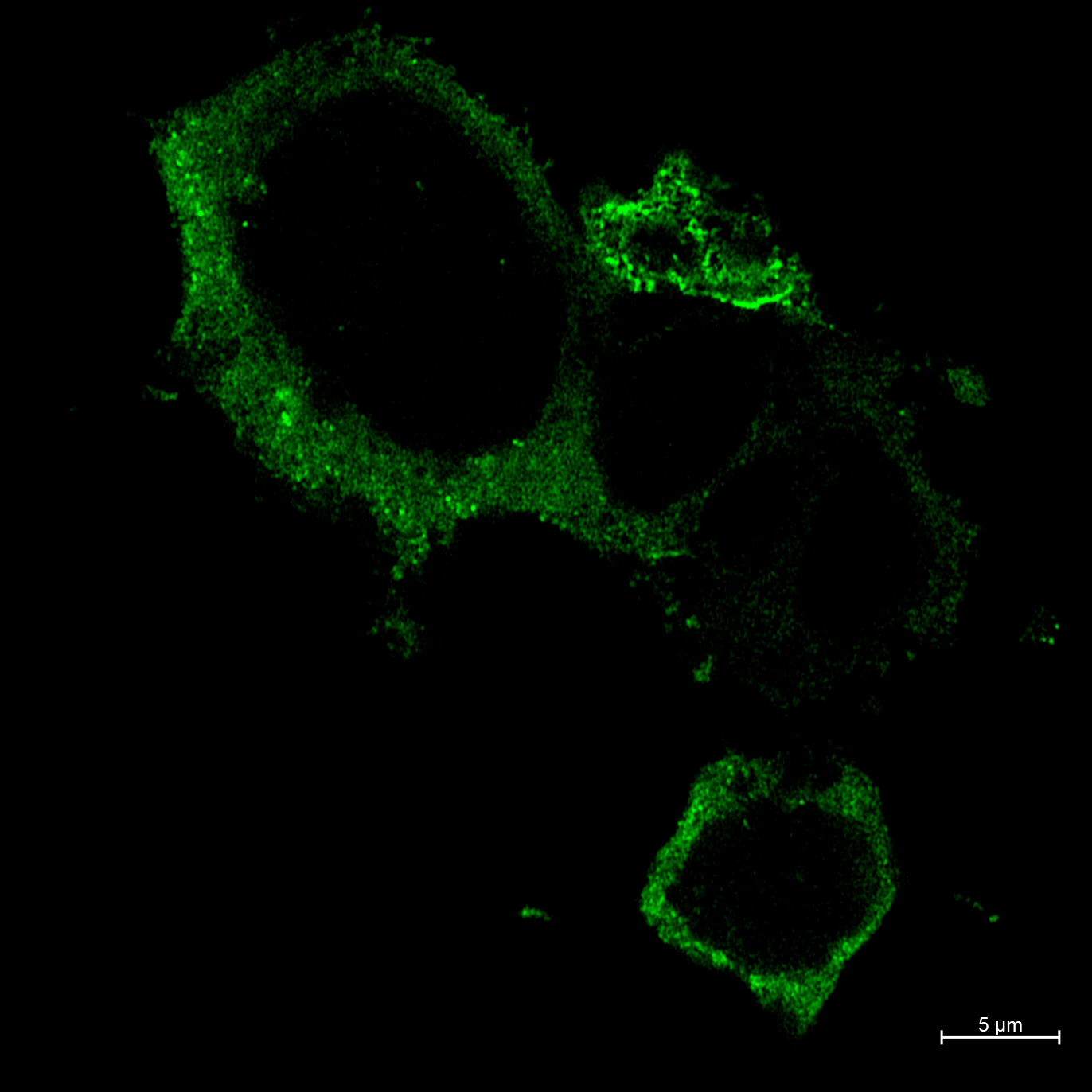

Supplement: Supplementary file 10 — Source data Fig. 7 [file 44318_2024_193_MOESM10_ESM.zip › Figure 7/7A/7A images/7A SPCS3.tif]

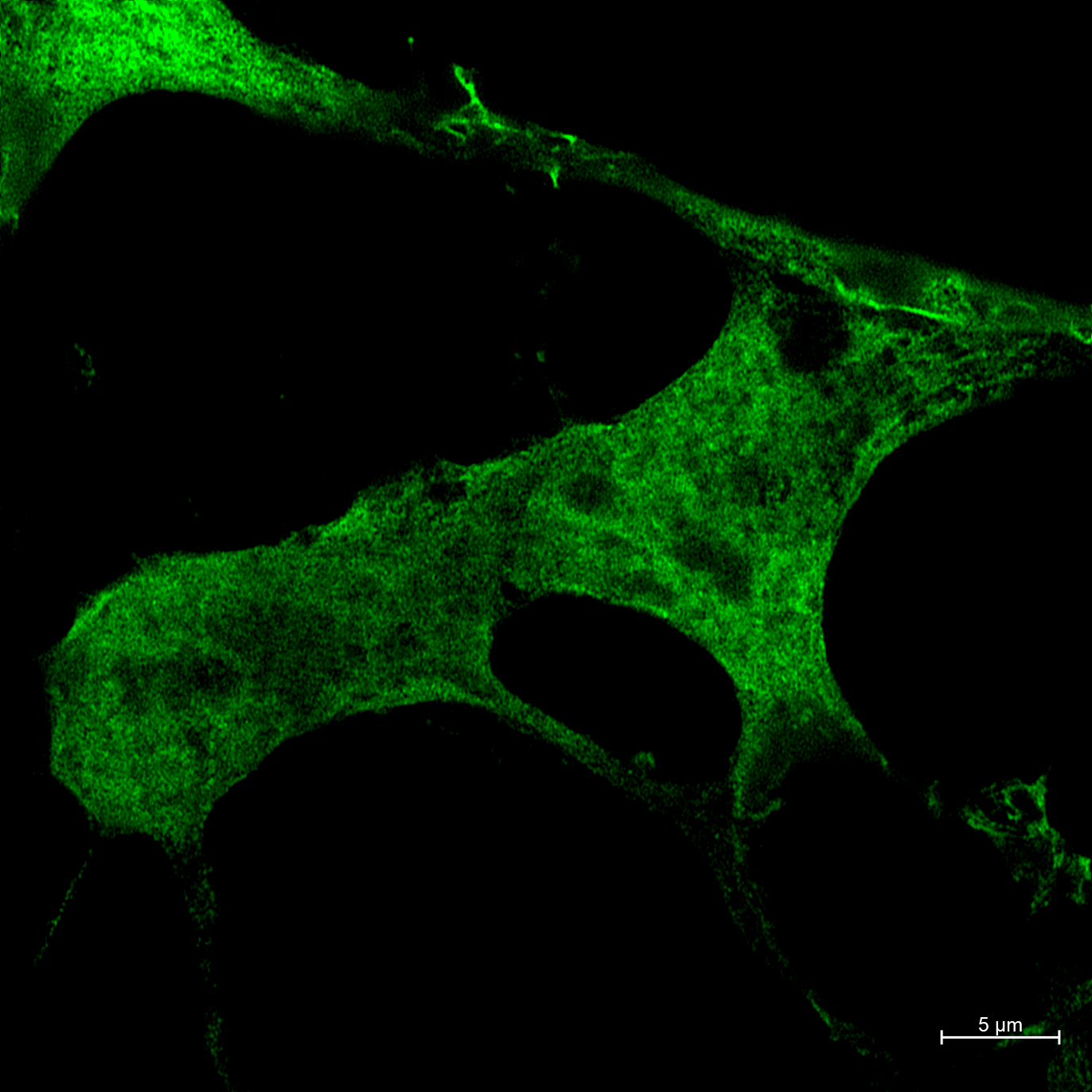

Supplement: Supplementary file 10 — Source data Fig. 7 [file 44318_2024_193_MOESM10_ESM.zip › Figure 7/7D/7D images/mock/7D-mock eIF3k.tif]

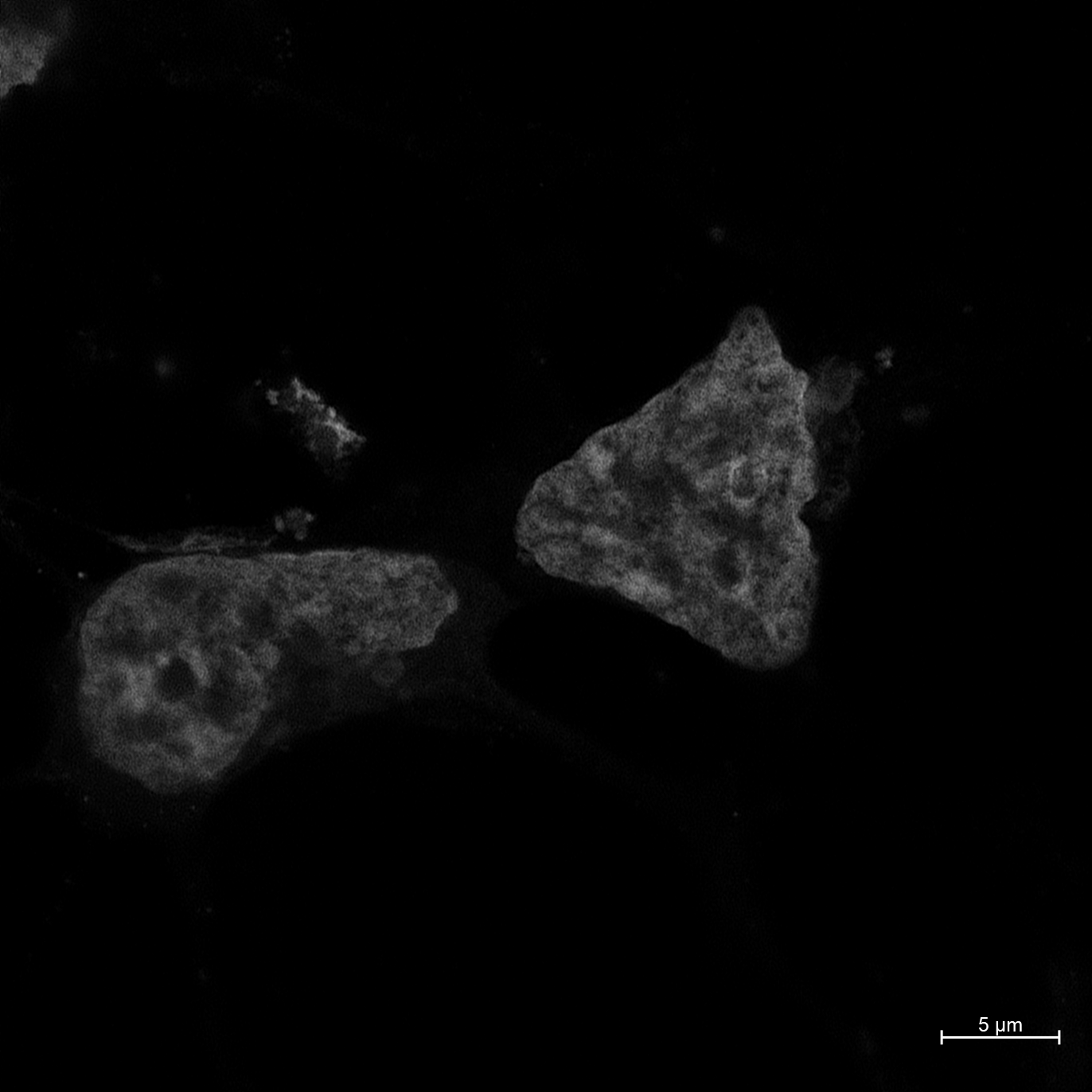

Supplement: Supplementary file 10 — Source data Fig. 7 [file 44318_2024_193_MOESM10_ESM.zip › Figure 7/7D/7D images/mock/7D-mock DAPI.tif]

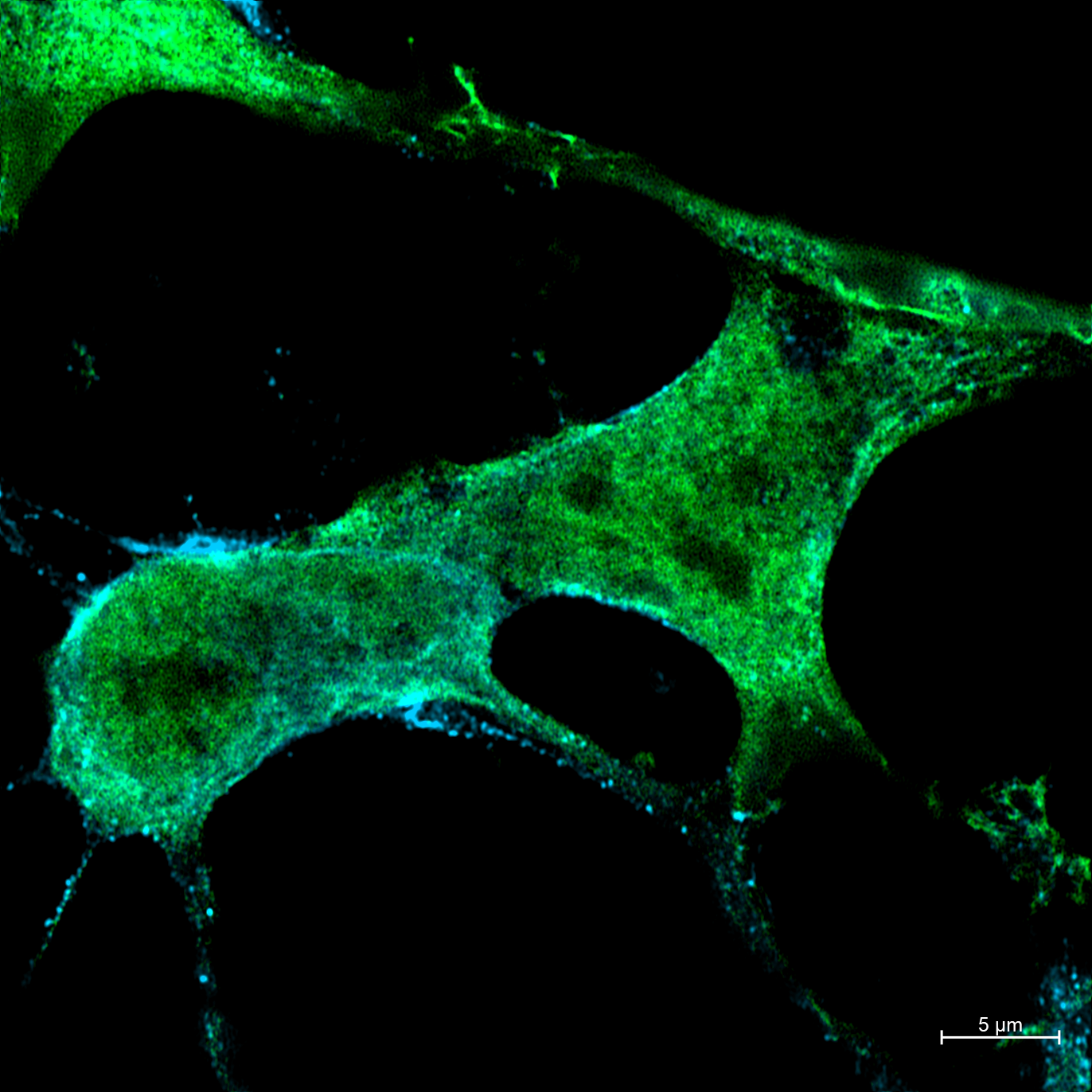

Supplement: Supplementary file 10 — Source data Fig. 7 [file 44318_2024_193_MOESM10_ESM.zip › Figure 7/7D/7D images/mock/7D-mock SPCS3 + eIF3k.tif]

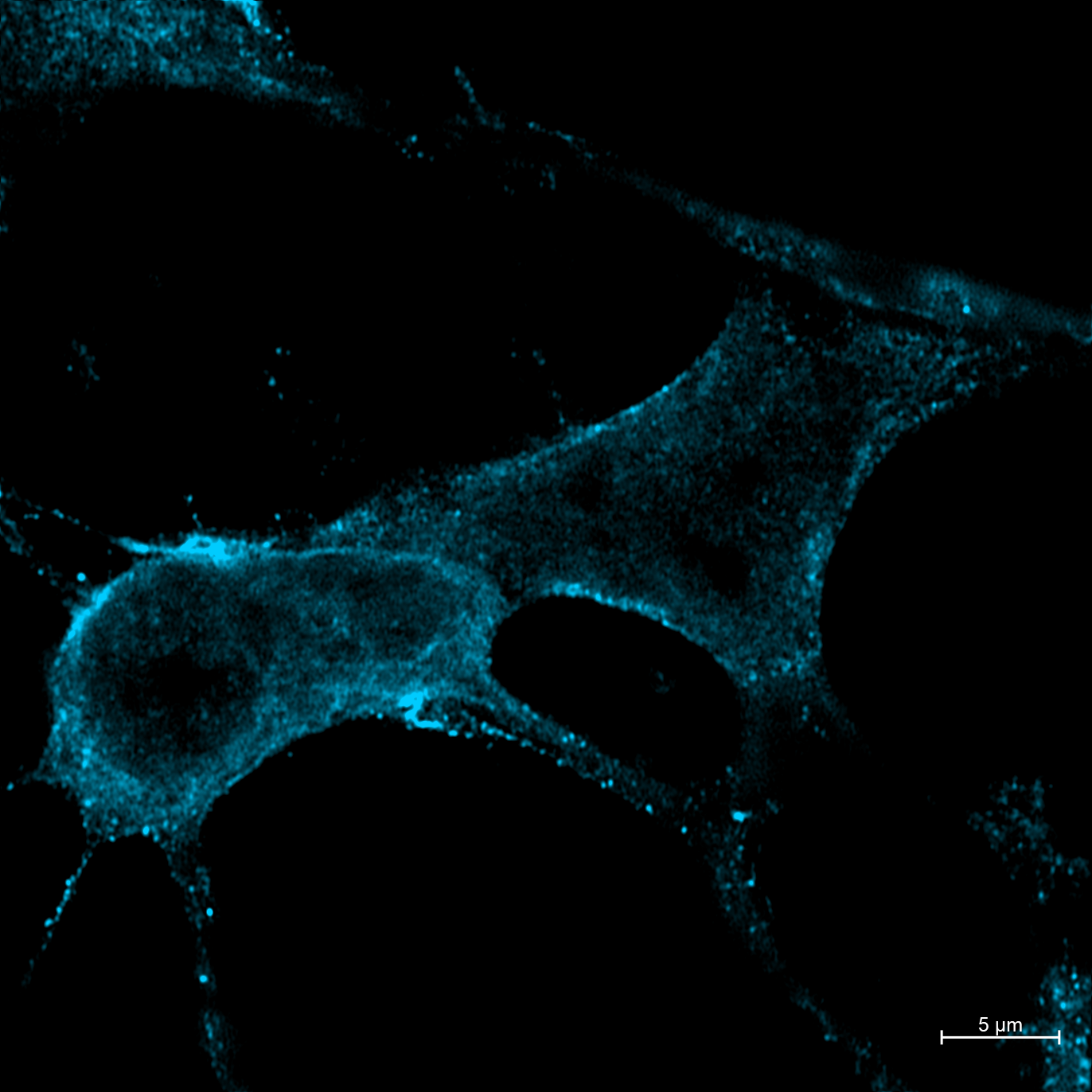

Supplement: Supplementary file 10 — Source data Fig. 7 [file 44318_2024_193_MOESM10_ESM.zip › Figure 7/7D/7D images/mock/7D-mock SPCS3.tif]

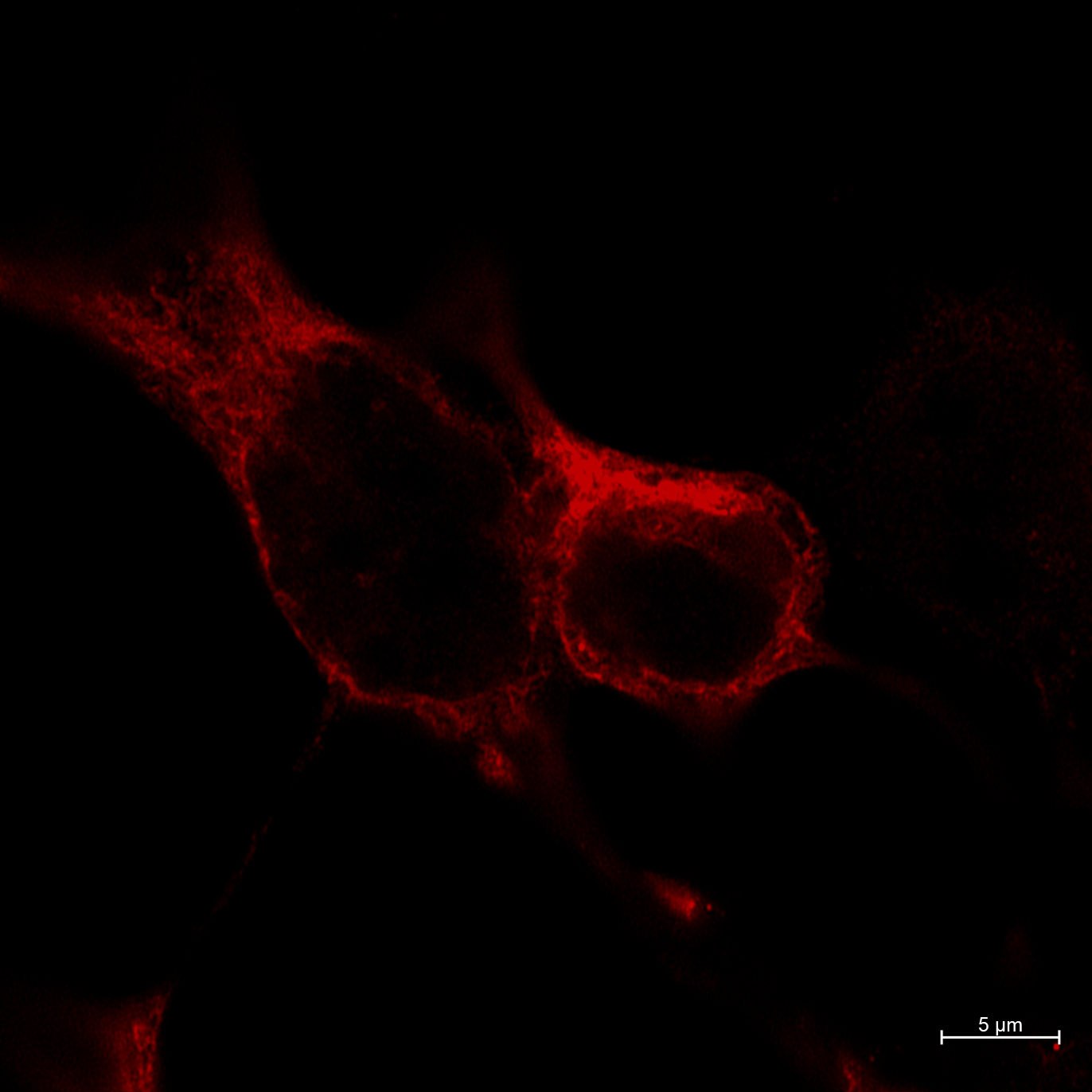

Supplement: Supplementary file 10 — Source data Fig. 7 [file 44318_2024_193_MOESM10_ESM.zip › Figure 7/7D/7D images/infected/7D-infected E1.tif]

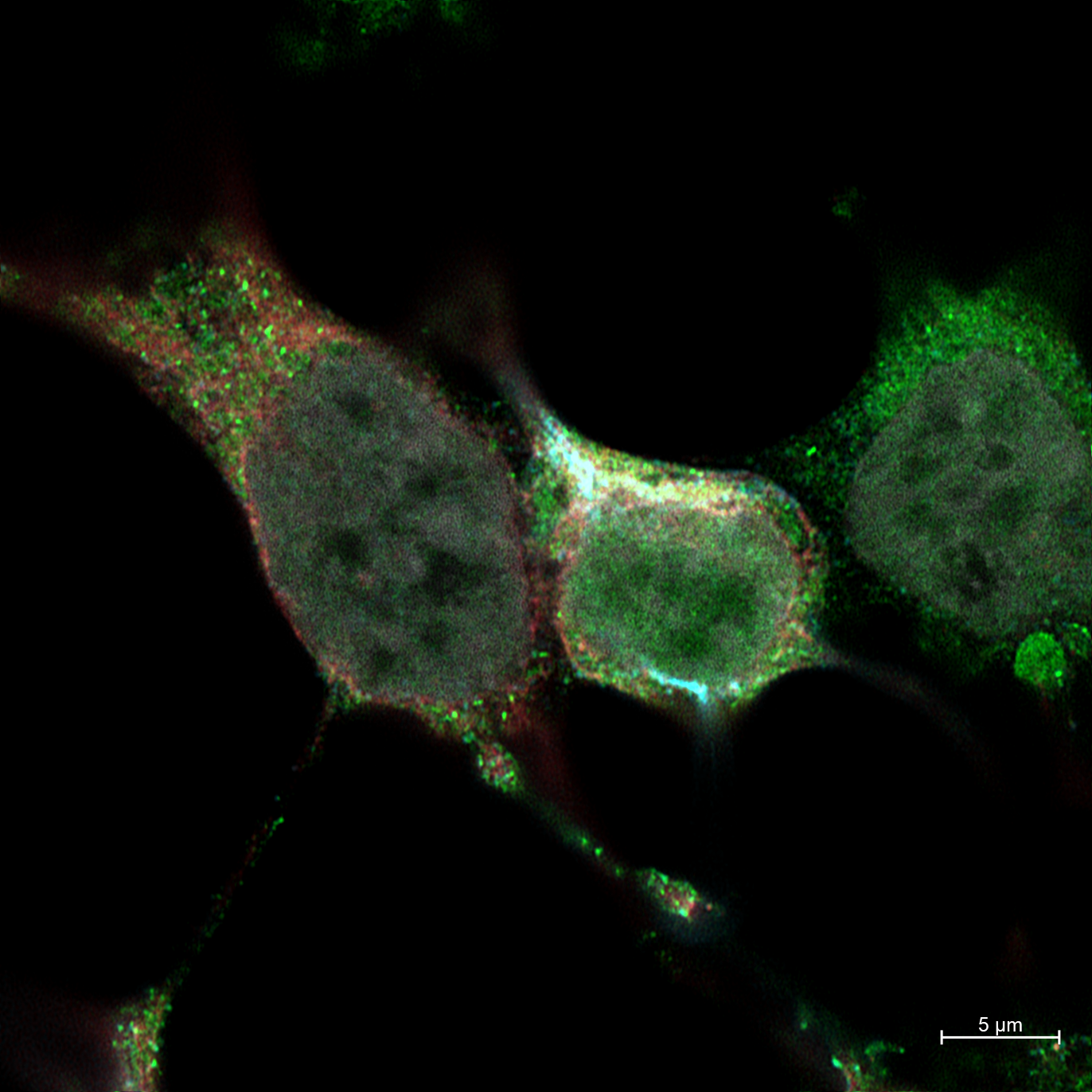

Supplement: Supplementary file 10 — Source data Fig. 7 [file 44318_2024_193_MOESM10_ESM.zip › Figure 7/7D/7D images/infected/ 7D-infected merged eIF3k+SPCS3+E1.tif]

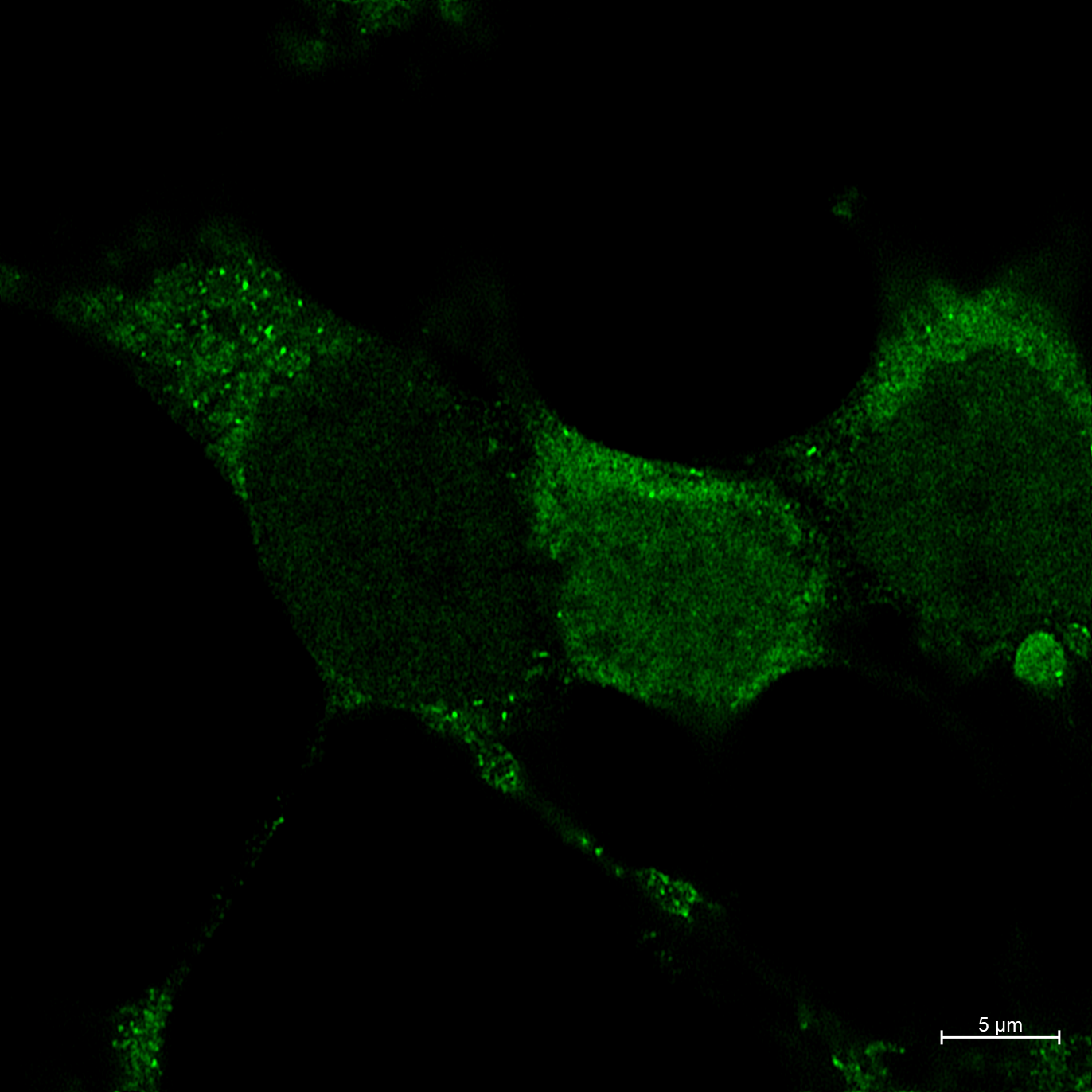

Supplement: Supplementary file 10 — Source data Fig. 7 [file 44318_2024_193_MOESM10_ESM.zip › Figure 7/7D/7D images/infected/7D-infected eIF3k.tif]

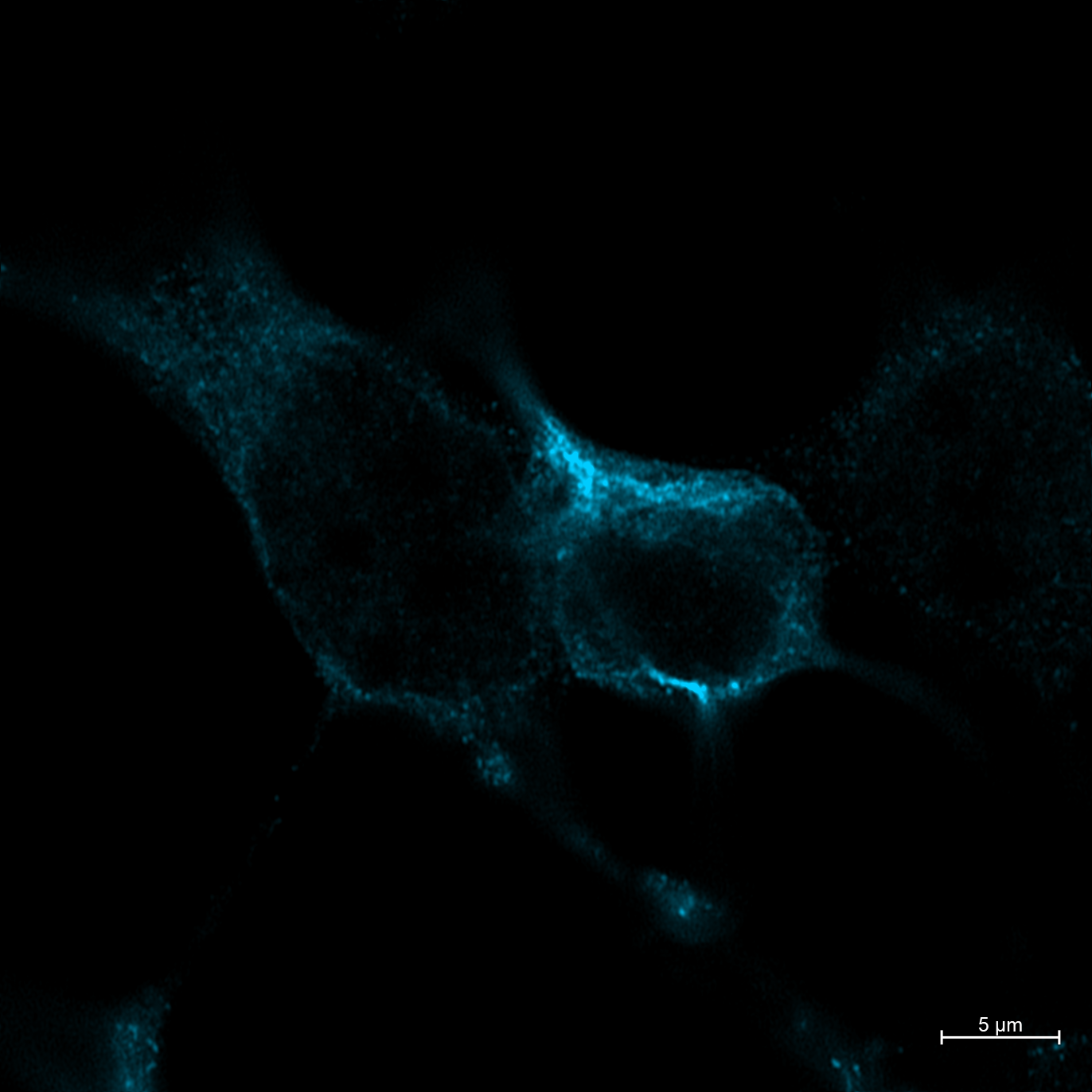

Supplement: Supplementary file 10 — Source data Fig. 7 [file 44318_2024_193_MOESM10_ESM.zip › Figure 7/7D/7D images/infected/7D-infected SPCS3.tif]

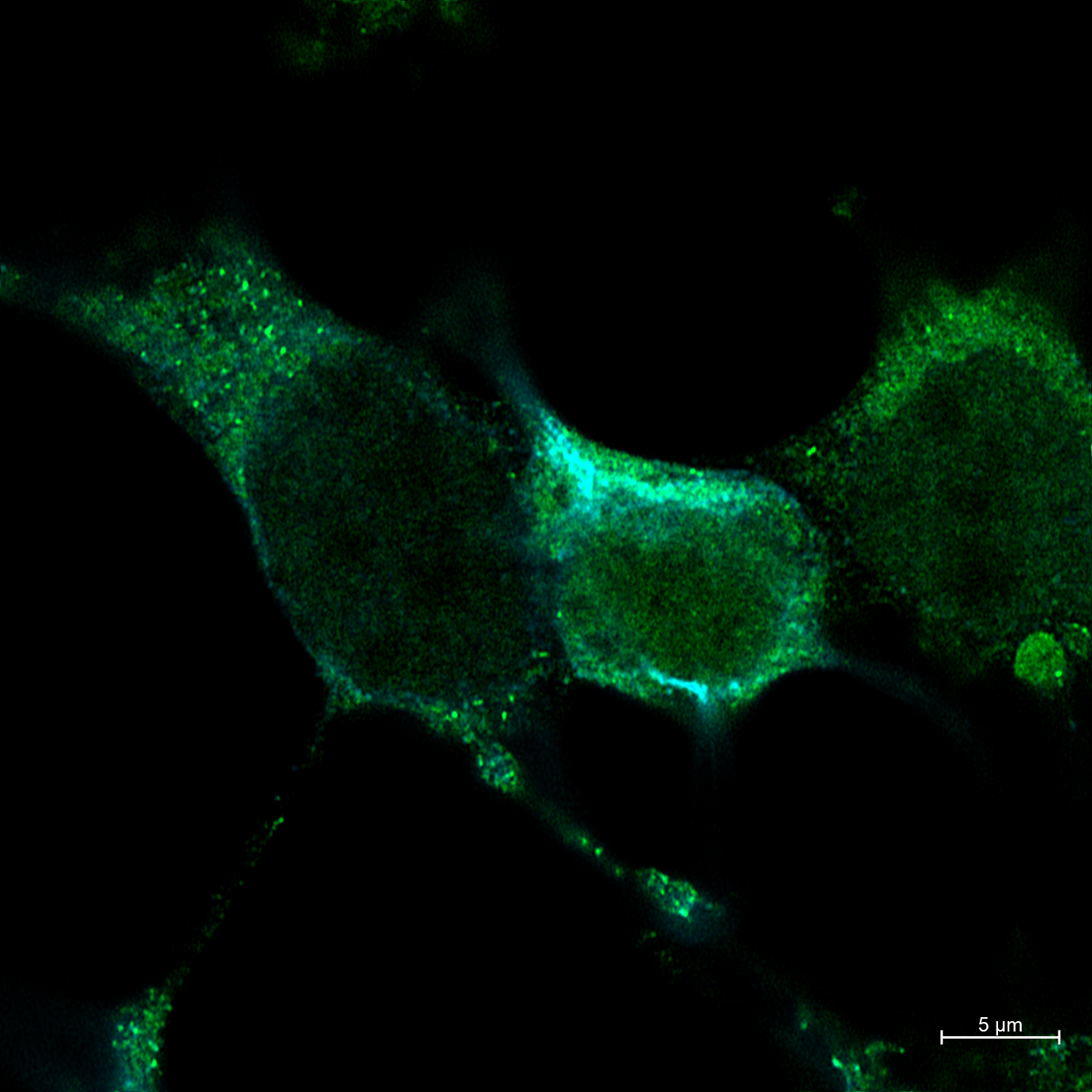

Supplement: Supplementary file 10 — Source data Fig. 7 [file 44318_2024_193_MOESM10_ESM.zip › Figure 7/7D/7D images/infected/7D-infected merged SPCS3+eIF3k.tif]

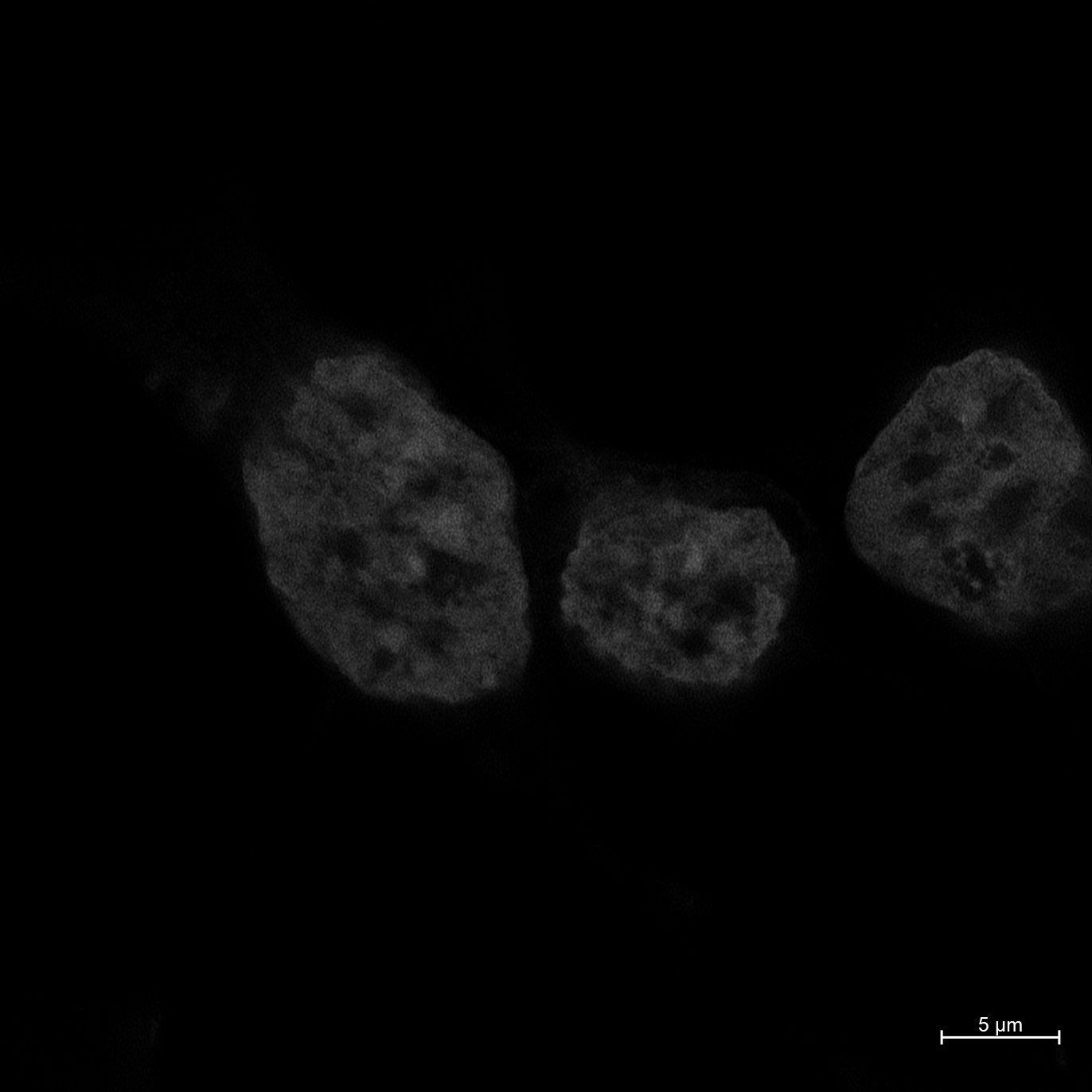

Supplement: Supplementary file 10 — Source data Fig. 7 [file 44318_2024_193_MOESM10_ESM.zip › Figure 7/7D/7D images/infected/7D-infected DAPI.tif]

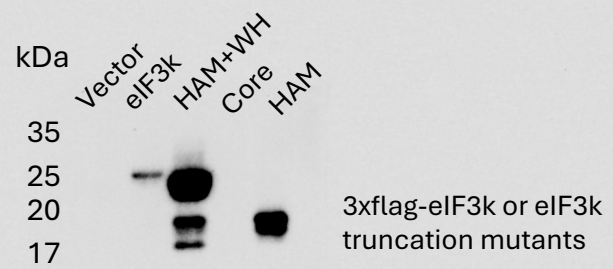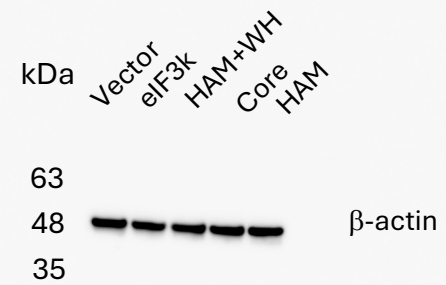

Supplement: Supplementary file 11 — Source data Fig. 8 [file 44318_2024_193_MOESM11_ESM.zip › Figure 8/8G/8G WB images.pdf]

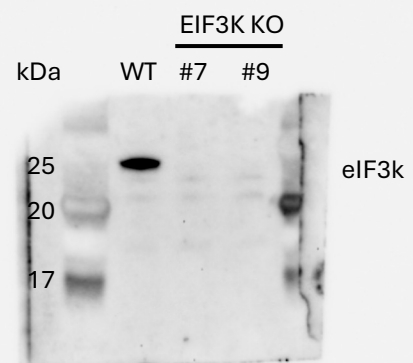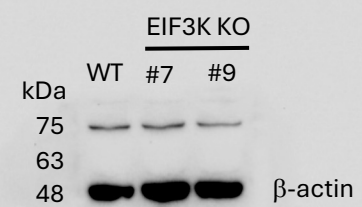

Supplement: Supplementary file 11 — Source data Fig. 8 [file 44318_2024_193_MOESM11_ESM.zip › Figure 8/8A/8A WB images.pdf]

## Slide 1
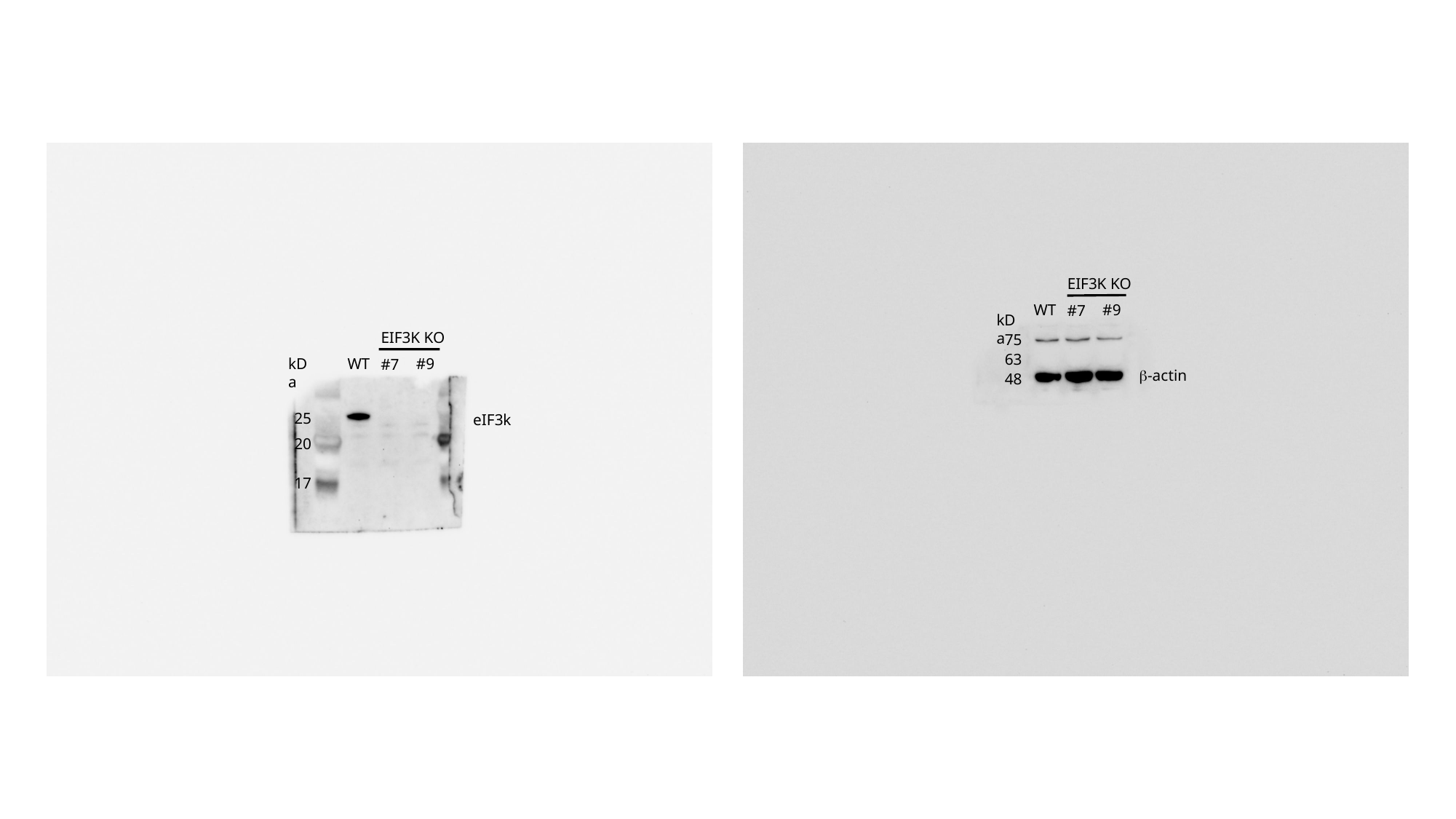

EIF3K KO
WT
#9
#7
kDa
EIF3K KO
75
63
kDa
WT
#9
#7
b-actin
48
25
eIF3k
20
17

Supplement: Supplementary file 11 — Source data Fig. 8 [file 44318_2024_193_MOESM11_ESM.zip › Figure 8/8A/8A WB images.pptx]
