# Supplementary material for: Interaction of chikungunya virus glycoproteins with macrophage factors controls virion production
Source: EMBO J. 2024 Sep 11;43(20):4625–55. doi: 10.1038/s44318-024-00193-3 (PMC11480453; doi:10.1038/s44318-024-00193-3)
Supplement: Supplementary file 8 — Source data Fig. 4 [file 44318_2024_193_MOESM8_ESM.zip › Figure 4/4E/4E image description.pptx]

## Slide 1
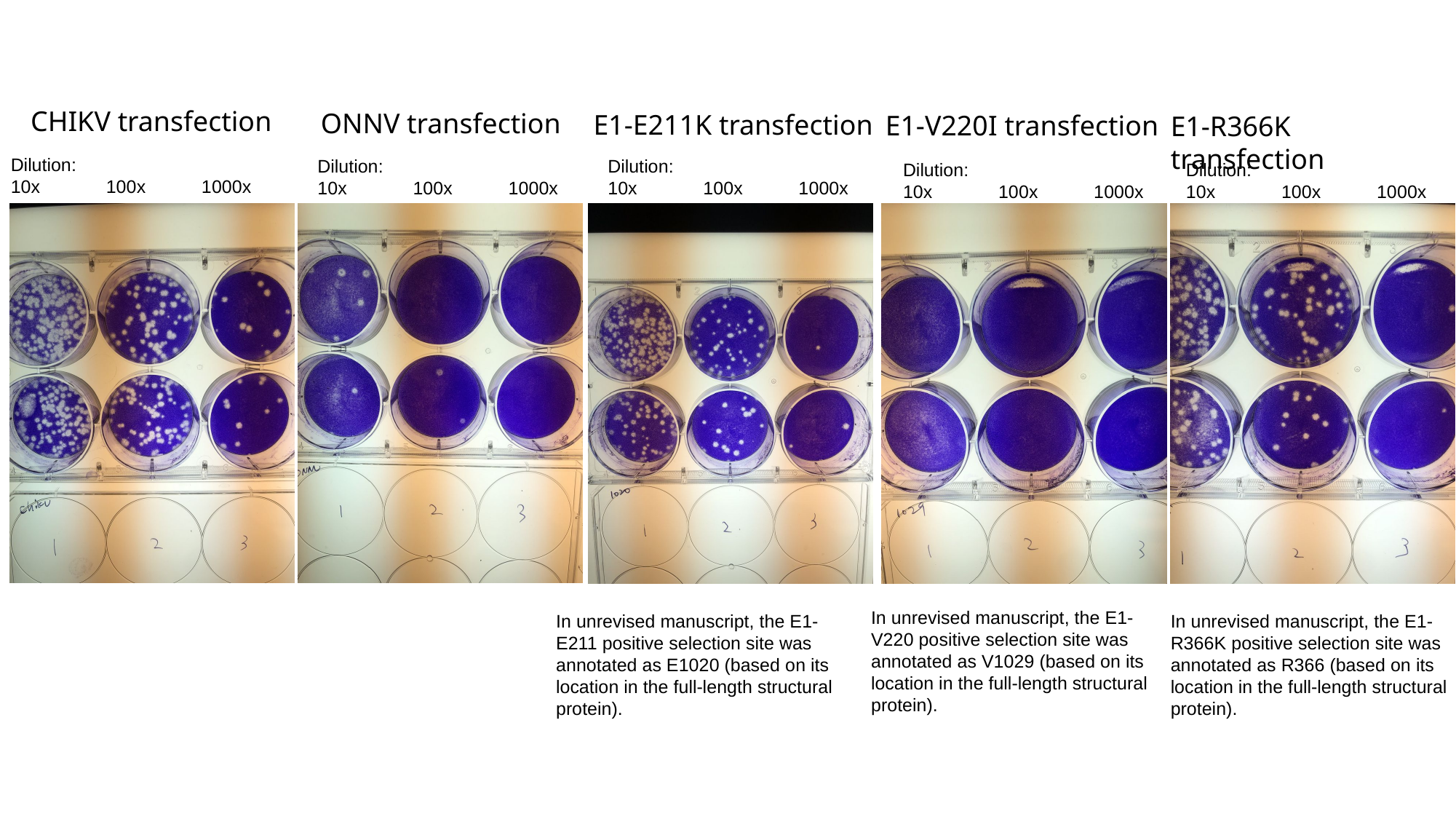

CHIKV transfection
ONNV transfection
E1-E211K transfection
E1-V220I transfection
E1-R366K transfection
Dilution:
10x 100x 1000x
Dilution:
10x 100x 1000x
Dilution:
10x 100x 1000x
Dilution:
10x 100x 1000x
Dilution:
10x 100x 1000x
In unrevised manuscript, the E1-V220 positive selection site was annotated as V1029 (based on its location in the full-length structural protein).
In unrevised manuscript, the E1-R366K positive selection site was annotated as R366 (based on its location in the full-length structural protein).
In unrevised manuscript, the E1-E211 positive selection site was annotated as E1020 (based on its location in the full-length structural protein).
