# Supplementary material for: Interaction of chikungunya virus glycoproteins with macrophage factors controls virion production
Source: EMBO J. 2024 Sep 11;43(20):4625–55. doi: 10.1038/s44318-024-00193-3 (PMC11480453; doi:10.1038/s44318-024-00193-3)
Supplement: Supplementary file 8 — Source data Fig. 4 [file 44318_2024_193_MOESM8_ESM.zip › Figure 4/4C/Data description.docx]

CSV files: The P value of each codon site in CHIKV structural protein is measured by FEL and MEME analysis.

JSON files: The complete positive selection reports (output as JSON files) are generated by HyPhy and can be read through Python or DataMonkey Server.
