# Supplementary material for: Interaction of chikungunya virus glycoproteins with macrophage factors controls virion production
Source: EMBO J. 2024 Sep 11;43(20):4625–55. doi: 10.1038/s44318-024-00193-3 (PMC11480453; doi:10.1038/s44318-024-00193-3)
Supplement: Supplementary file 9 — Source data Fig. 6 [file 44318_2024_193_MOESM9_ESM.zip › Figure 6/6D/6D WB image.pptx]

## Slide 1
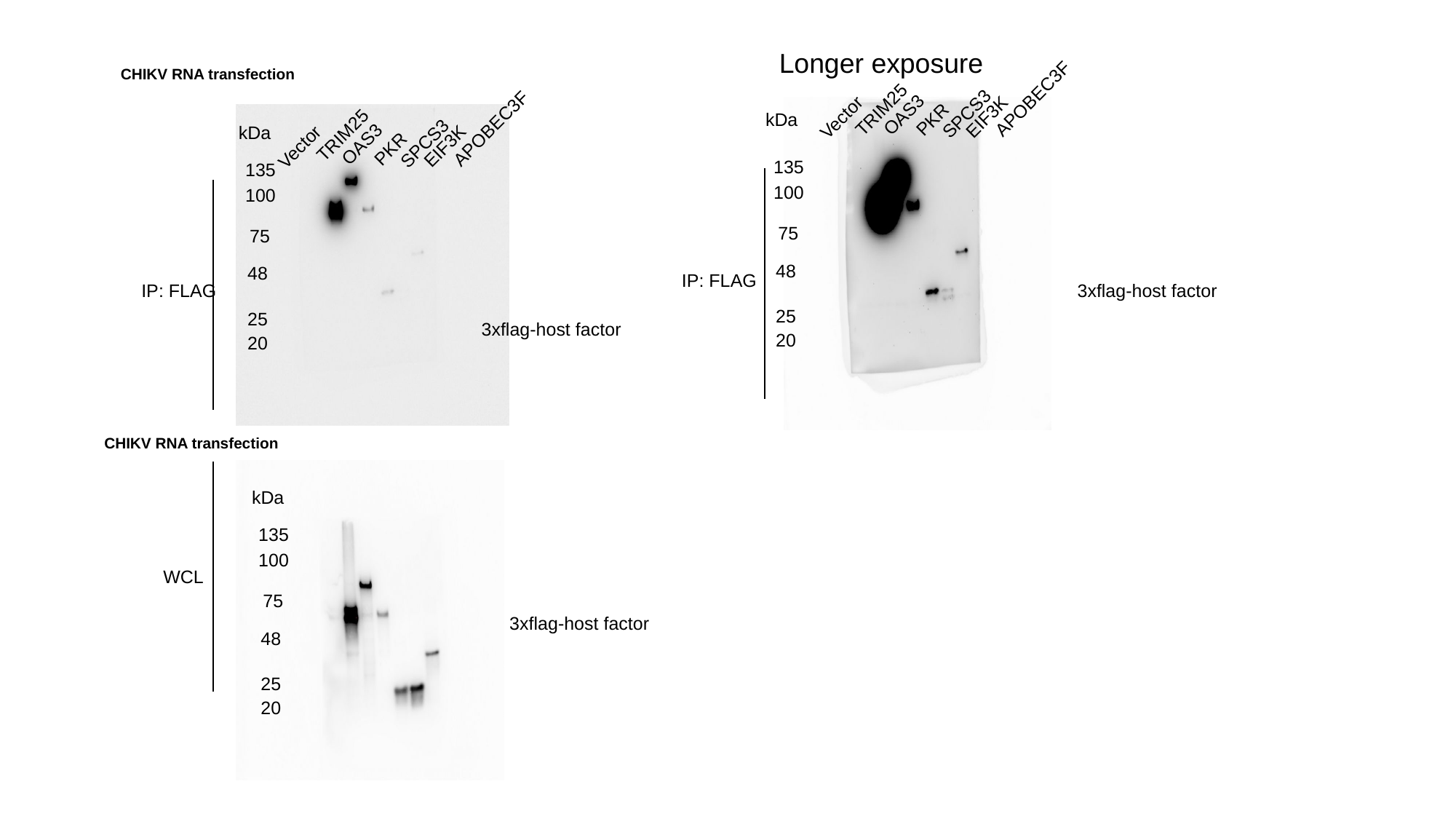

Longer exposure
CHIKV RNA transfection
APOBEC3F
OAS3
PKR
TRIM25
SPCS3
Vector
EIF3K
APOBEC3F
kDa
TRIM25
kDa
OAS3
PKR
SPCS3
Vector
EIF3K
135
135
100
100
75
75
48
48
IP: FLAG
3xflag-host factor
IP: FLAG
25
25
3xflag-host factor
20
20
CHIKV RNA transfection
kDa
135
100
WCL
75
3xflag-host factor
48
25
20

## Slide 2
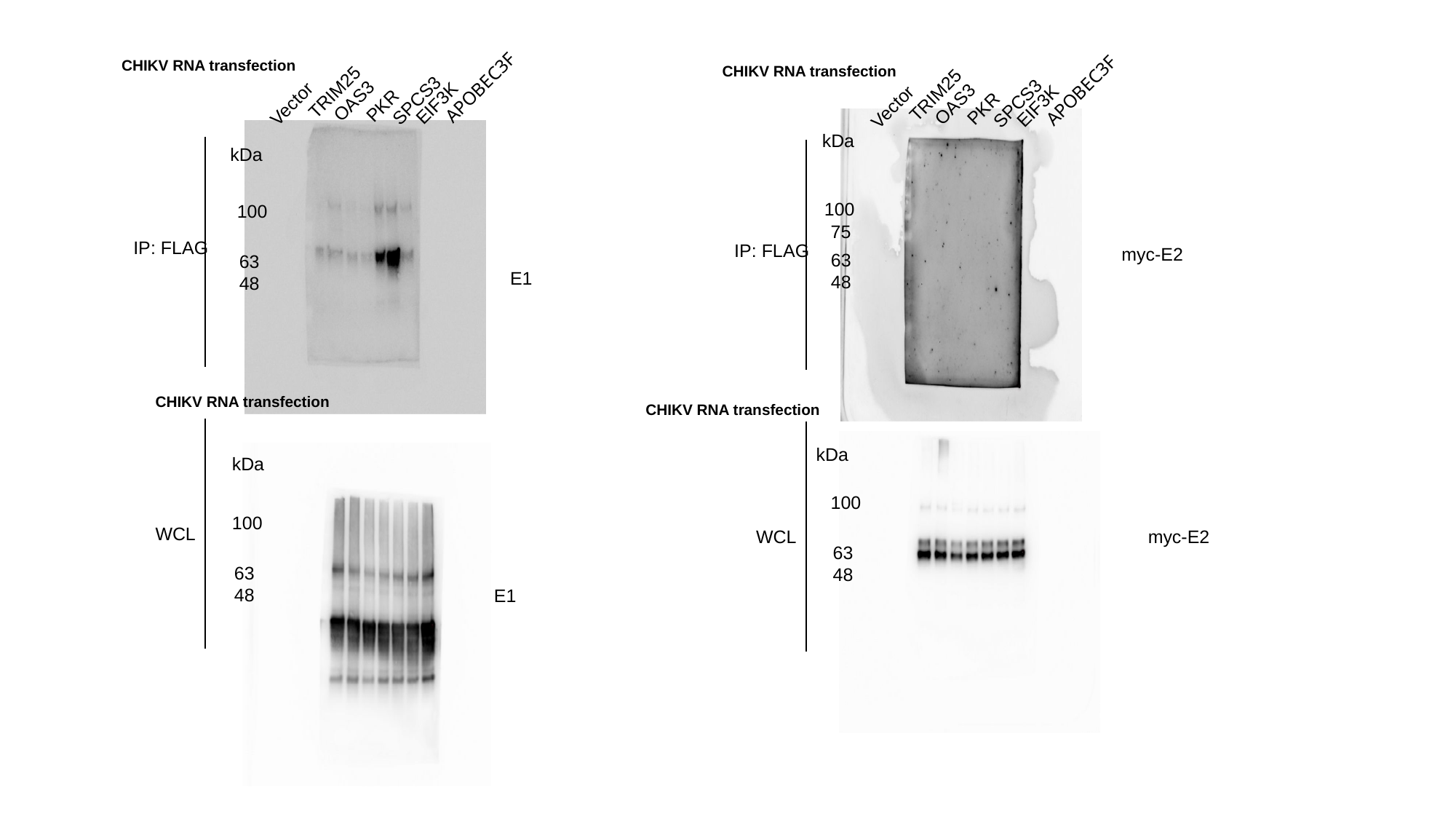

CHIKV RNA transfection
APOBEC3F
CHIKV RNA transfection
APOBEC3F
TRIM25
OAS3
TRIM25
PKR
SPCS3
OAS3
Vector
EIF3K
PKR
SPCS3
Vector
EIF3K
kDa
kDa
100
100
75
IP: FLAG
IP: FLAG
myc-E2
63
63
E1
48
48
CHIKV RNA transfection
CHIKV RNA transfection
kDa
kDa
100
100
WCL
myc-E2
WCL
63
63
48
48
E1

## Slide 3
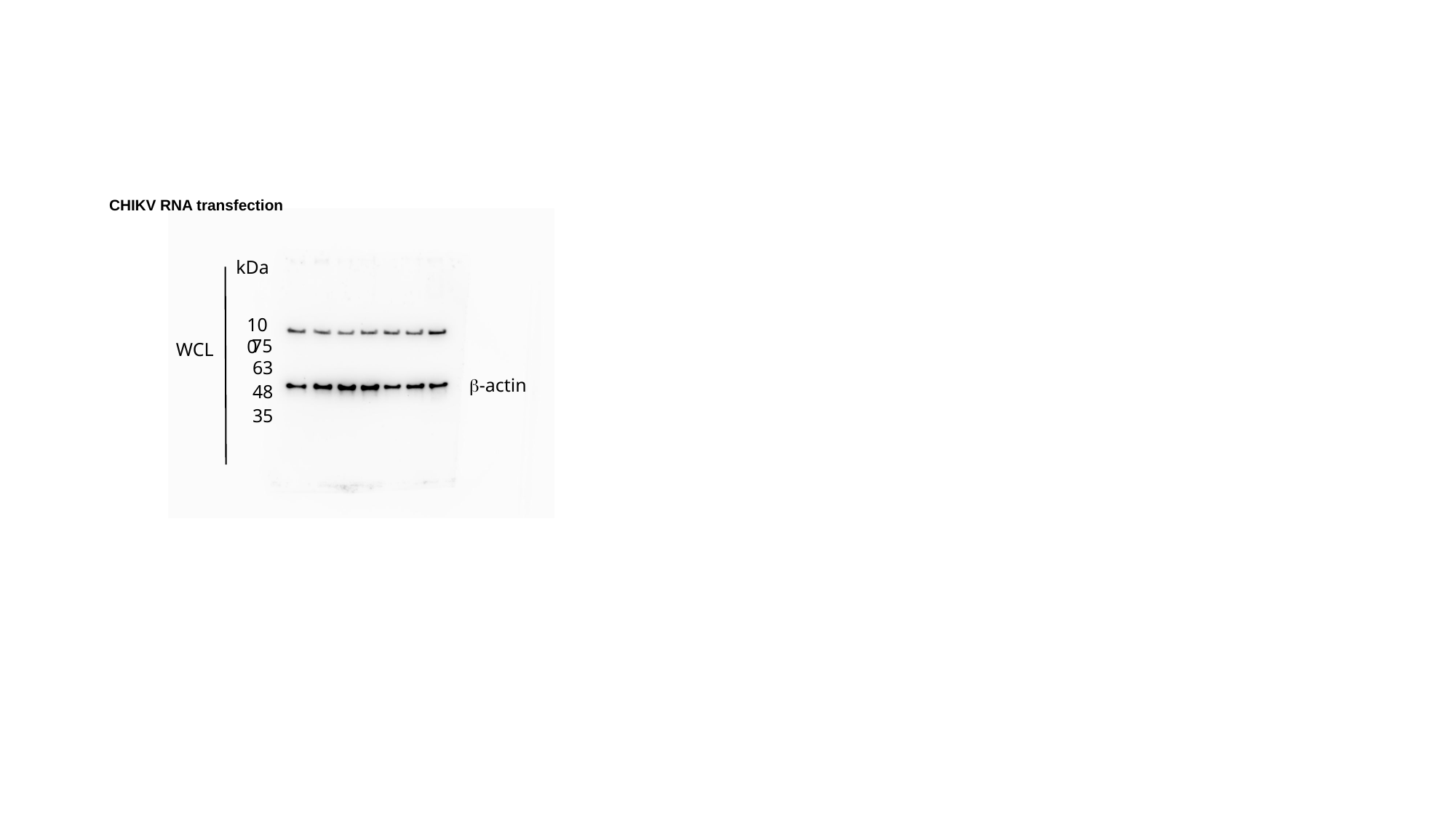

CHIKV RNA transfection
kDa
100
75
WCL
63
b-actin
48
35
