# Supplementary material for: Interaction of chikungunya virus glycoproteins with macrophage factors controls virion production
Source: EMBO J. 2024 Sep 11;43(20):4625–55. doi: 10.1038/s44318-024-00193-3 (PMC11480453; doi:10.1038/s44318-024-00193-3)

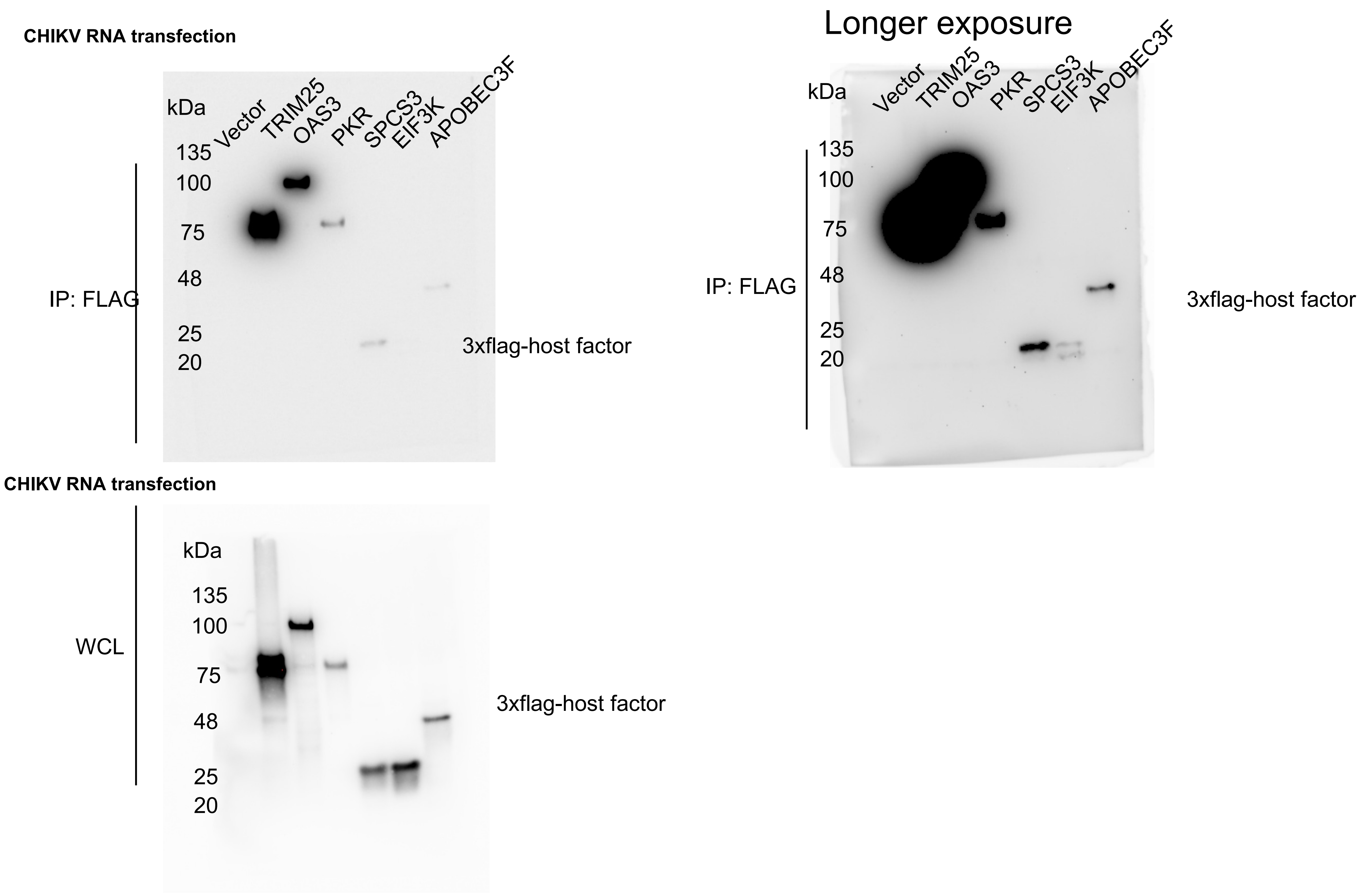

CHIKV RNA transfection
kDa
Vector
TRIM25
OAS3
PKR
SPCS3
EIF3K
APOBEC3F
135
100
75
48
25
20
IP: FLAG
3xflag-host factor
CHIKV RNA transfection
WCL
kDa
135
100
75
48
25
20
3xflag-host factor
Longer exposure
kDa
Vector
TRIM25
OAS3
PKR
SPCS3
EIF3K
APOBEC3F
135
100
75
48
25
20
IP: FLAG
3xflag-host factor

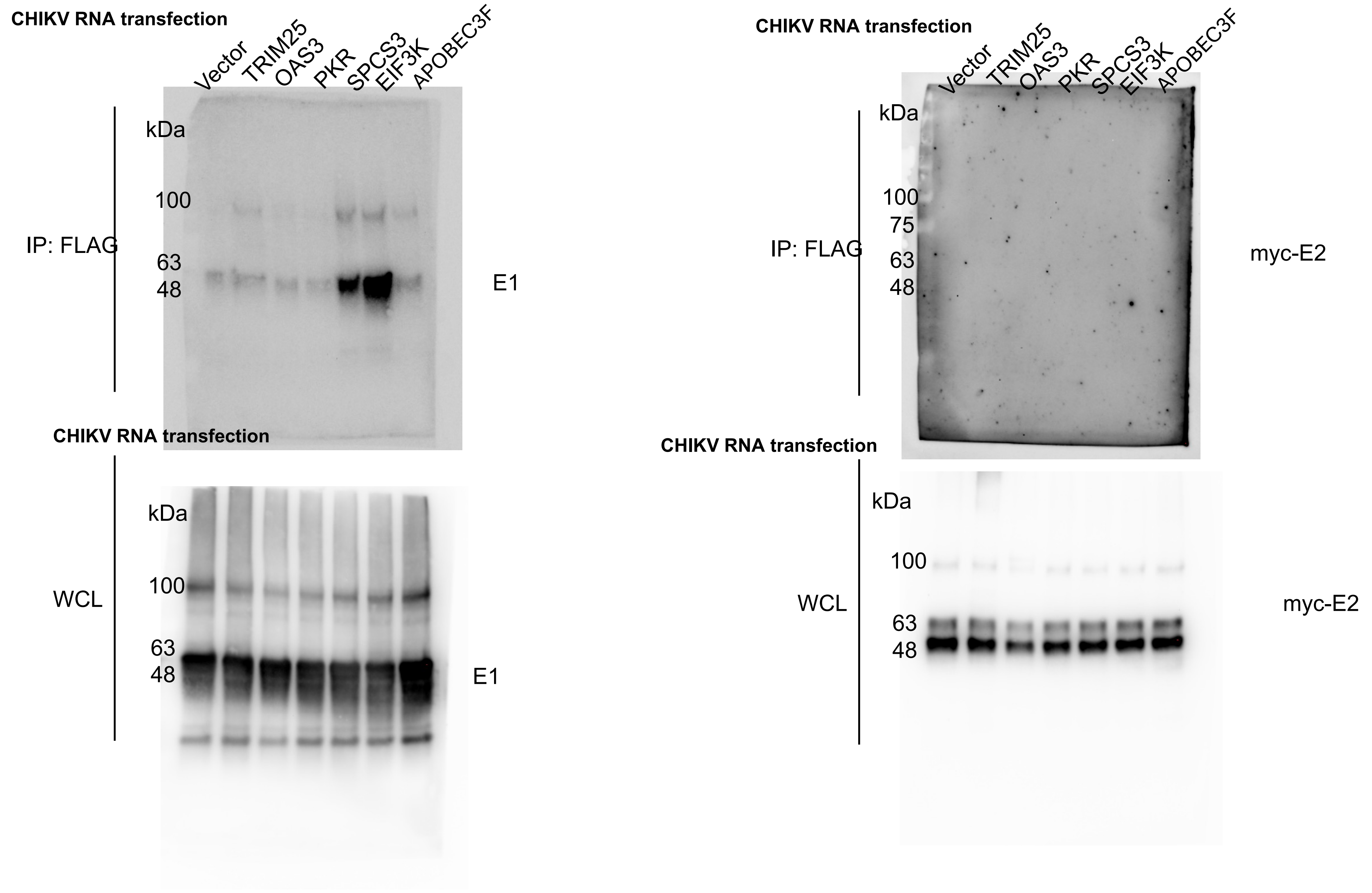
CHIKV RNA transfection
Vector
TRIM25
OAS3
PKR
SPCS3
EIF3K
APOBEC3F
kDa
100
63
48
IP: FLAG
E1
CHIKV RNA transfection
kDa
100
63
48
WCL
E1
CHIKV RNA transfection
Vector
TRIM25
OAS3
PKR
SPCS3
EIF3K
APOBEC3F
kDa
100
75
63
48
IP: FLAG
myc-E2
CHIKV RNA transfection
kDa
100
63
48
WCL
myc-E2

CHIKV RNA transfection

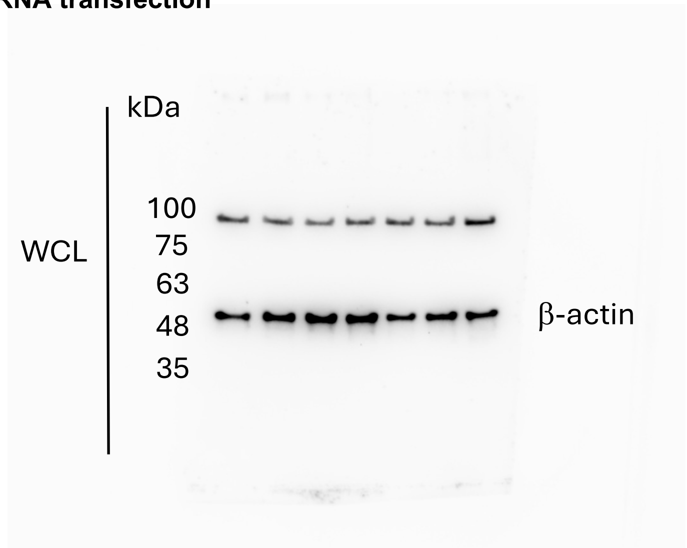

Supplement: Supplementary file 9 — Source data Fig. 6 [file 44318_2024_193_MOESM9_ESM.zip › Figure 6/6D/6D WB image.pdf]
