# Supplementary material for: Interaction of chikungunya virus glycoproteins with macrophage factors controls virion production
Source: EMBO J. 2024 Sep 11;43(20):4625–55. doi: 10.1038/s44318-024-00193-3 (PMC11480453; doi:10.1038/s44318-024-00193-3)

IP fraction

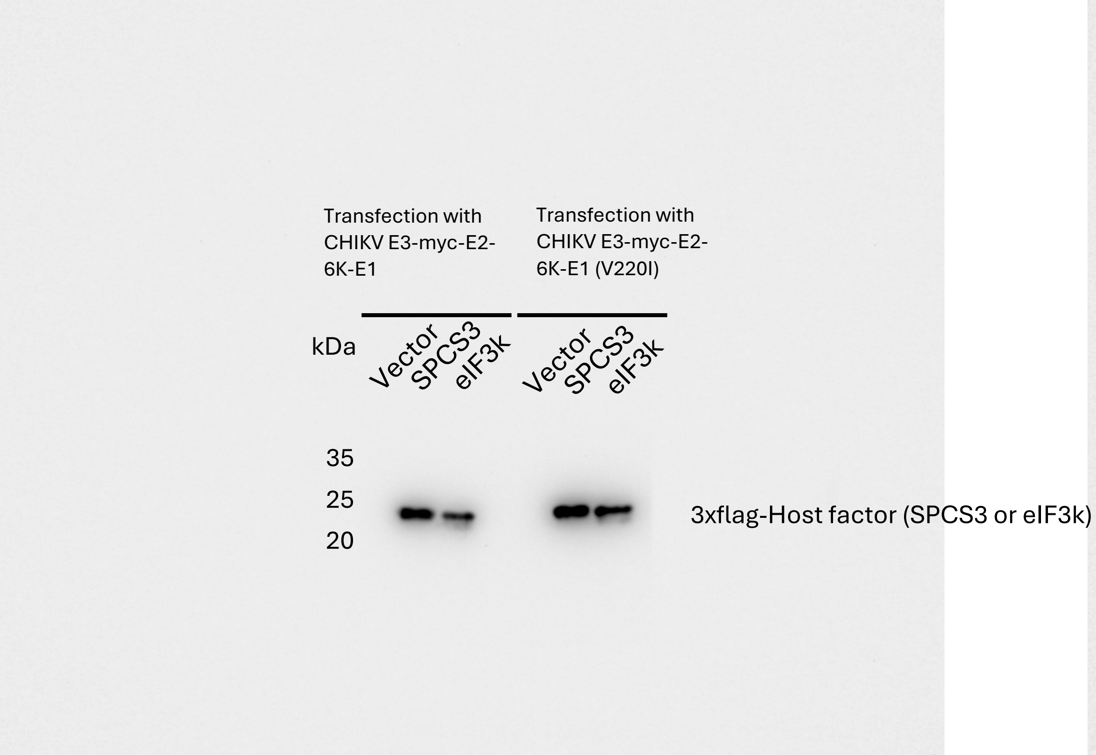
Transfection with CHIKV E3-myc-E2-6K-E1
Transfection with CHIKV E3-myc-E2-6K-E1 (V220I)
kDa
Vector
SPCS3
eIF3k
Vector
SPCS3
eIF3k
35
25
20
3xflag-Host factor (SPCS3 or eIF3k)

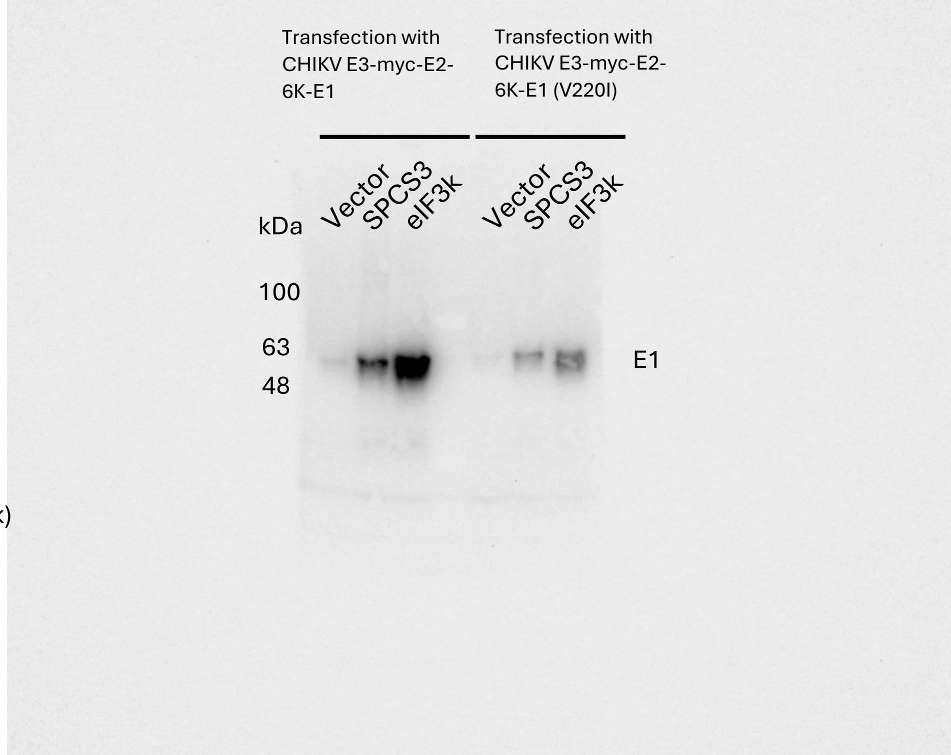
Transfection with CHIKV E3-myc-E2-6K-E1
Transfection with CHIKV E3-myc-E2-6K-E1 (V220I)
kDa
Vector
SPCS3
eIF3k
Vector
SPCS3
eIF3k
100
63
48
E1

WCL fraction

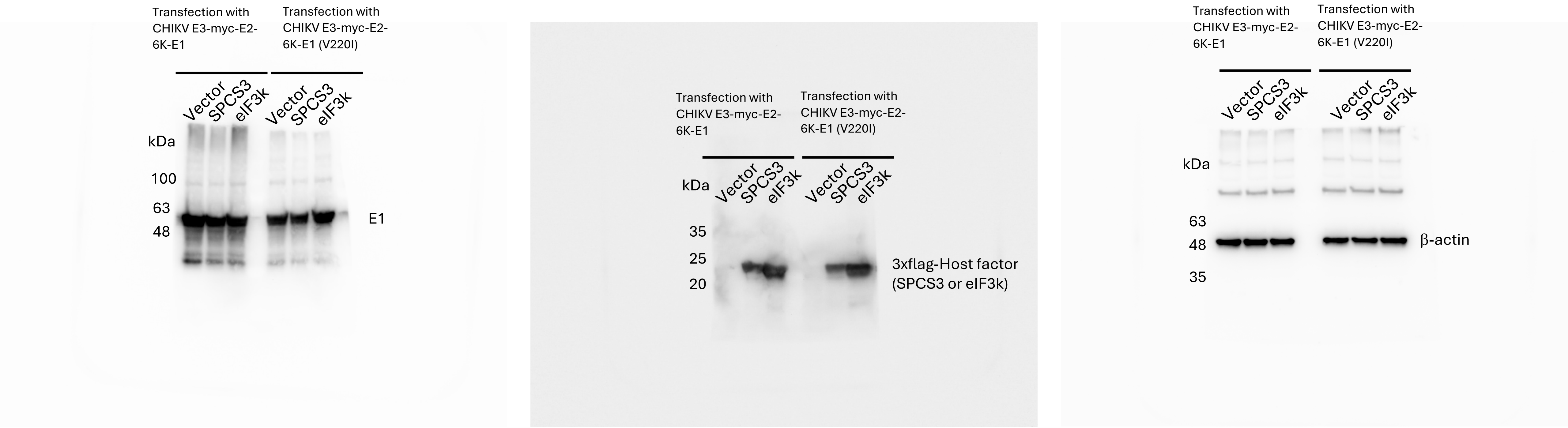

Supplement: Supplementary file 10 — Source data Fig. 7 [file 44318_2024_193_MOESM10_ESM.zip › Figure 7/7C/7C WB images.pdf]
