# Supplementary material for: Interaction of chikungunya virus glycoproteins with macrophage factors controls virion production
Source: EMBO J. 2024 Sep 11;43(20):4625–55. doi: 10.1038/s44318-024-00193-3 (PMC11480453; doi:10.1038/s44318-024-00193-3)
Supplement: Supplementary file 10 — Source data Fig. 7 [file 44318_2024_193_MOESM10_ESM.zip › Figure 7/7C/7C WB images.pptx]

## Slide 1
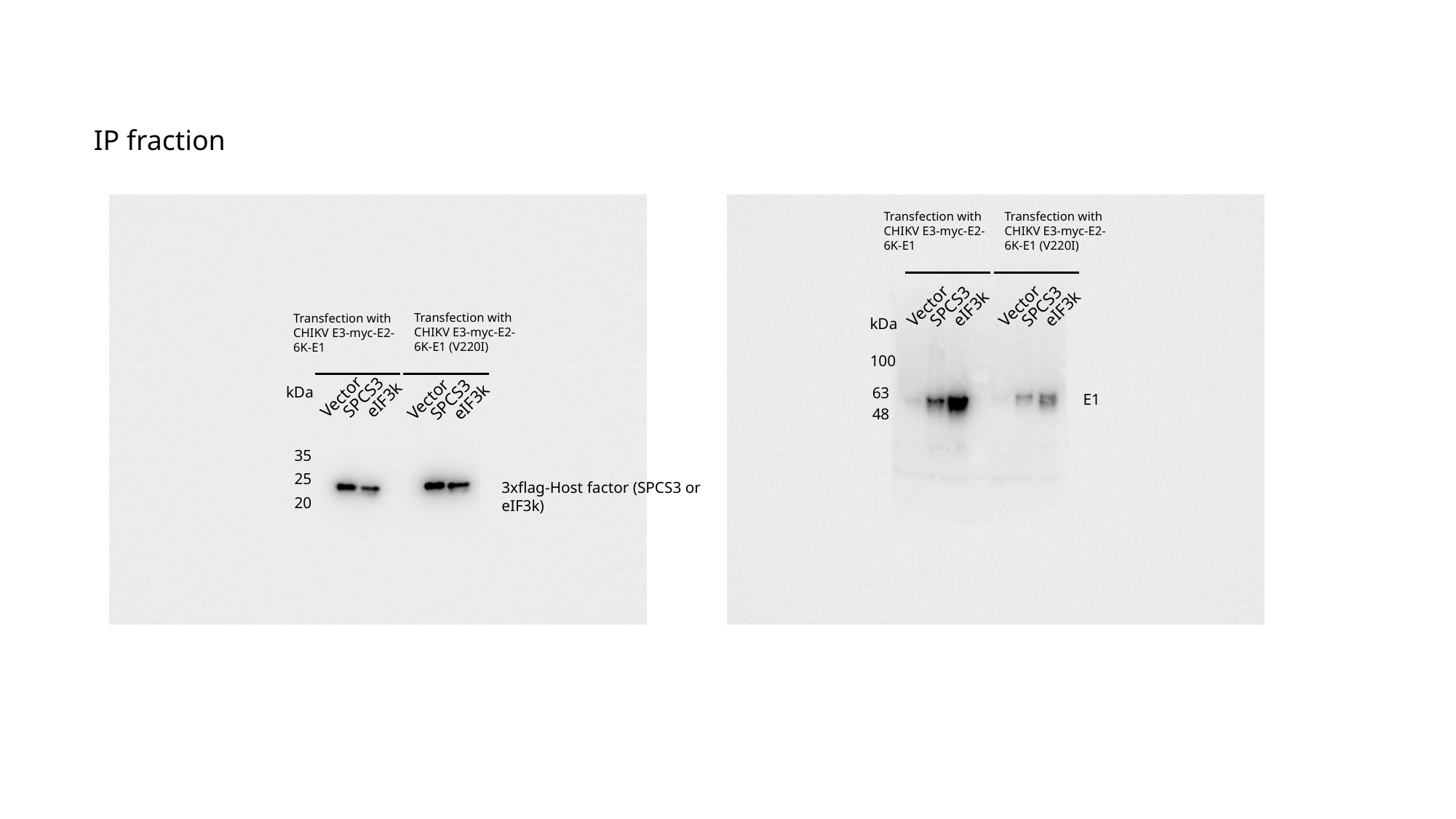

IP fraction
Transfection with CHIKV E3-myc-E2-6K-E1 (V220I)
Transfection with CHIKV E3-myc-E2-6K-E1
SPCS3
eIF3k
Vector
SPCS3
eIF3k
Vector
Transfection with CHIKV E3-myc-E2-6K-E1 (V220I)
Transfection with CHIKV E3-myc-E2-6K-E1
kDa
100
SPCS3
eIF3k
Vector
SPCS3
eIF3k
Vector
kDa
63
E1
48
35
25
3xflag-Host factor (SPCS3 or eIF3k)
20

## Slide 2
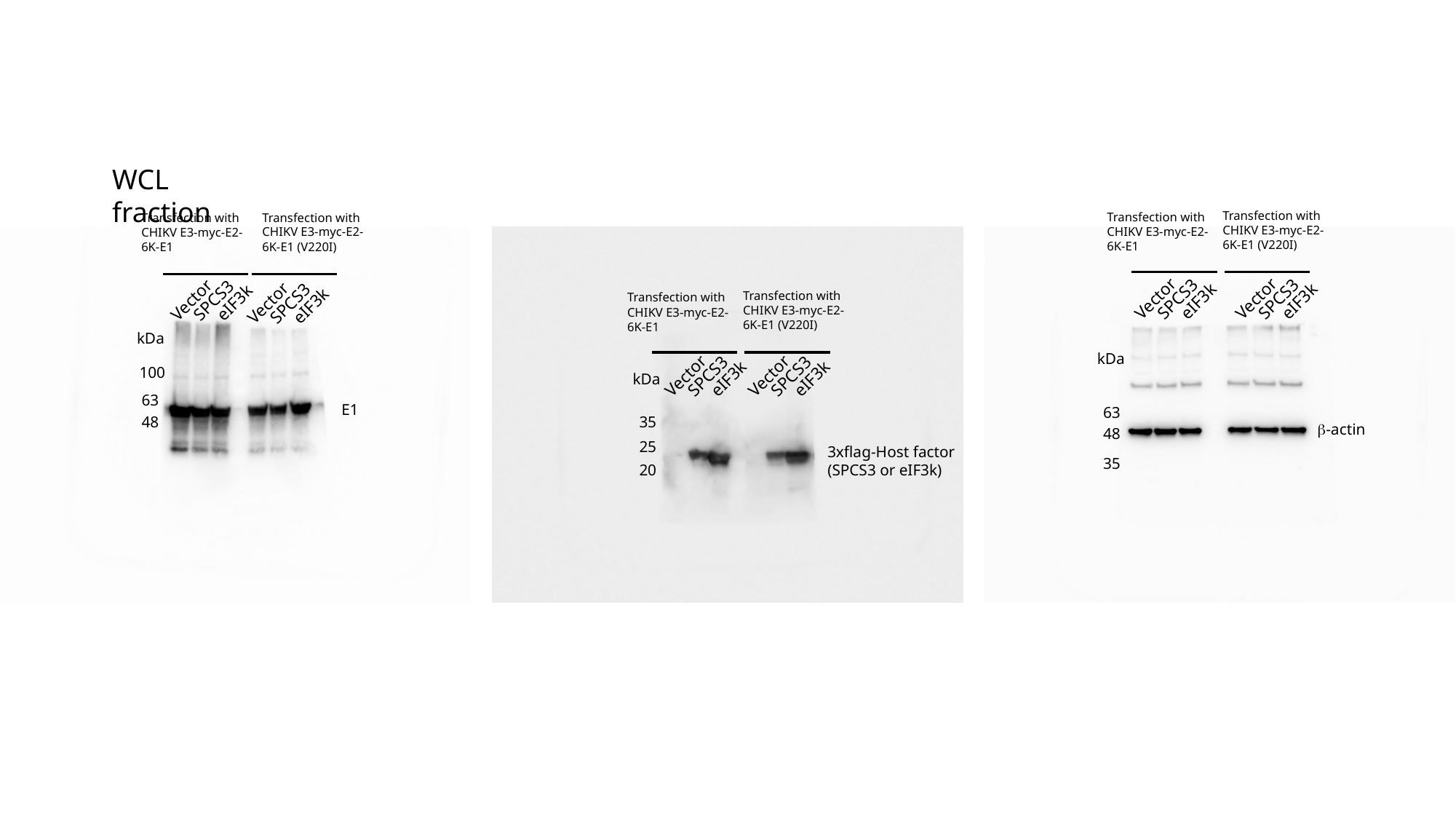

WCL fraction
Transfection with CHIKV E3-myc-E2-6K-E1 (V220I)
Transfection with CHIKV E3-myc-E2-6K-E1
Transfection with CHIKV E3-myc-E2-6K-E1 (V220I)
Transfection with CHIKV E3-myc-E2-6K-E1
SPCS3
eIF3k
Vector
SPCS3
eIF3k
Vector
SPCS3
eIF3k
Vector
SPCS3
eIF3k
Vector
Transfection with CHIKV E3-myc-E2-6K-E1 (V220I)
Transfection with CHIKV E3-myc-E2-6K-E1
kDa
kDa
SPCS3
eIF3k
Vector
SPCS3
eIF3k
Vector
100
kDa
63
E1
63
35
48
b-actin
48
25
3xflag-Host factor (SPCS3 or eIF3k)
35
20
