# Supplementary material for: Interaction of chikungunya virus glycoproteins with macrophage factors controls virion production
Source: EMBO J. 2024 Sep 11;43(20):4625–55. doi: 10.1038/s44318-024-00193-3 (PMC11480453; doi:10.1038/s44318-024-00193-3)
Supplement: Supplementary file 11 — Source data Fig. 8 [file 44318_2024_193_MOESM11_ESM.zip › Figure 8/8C-8D/8C-8D WB images.pdf]

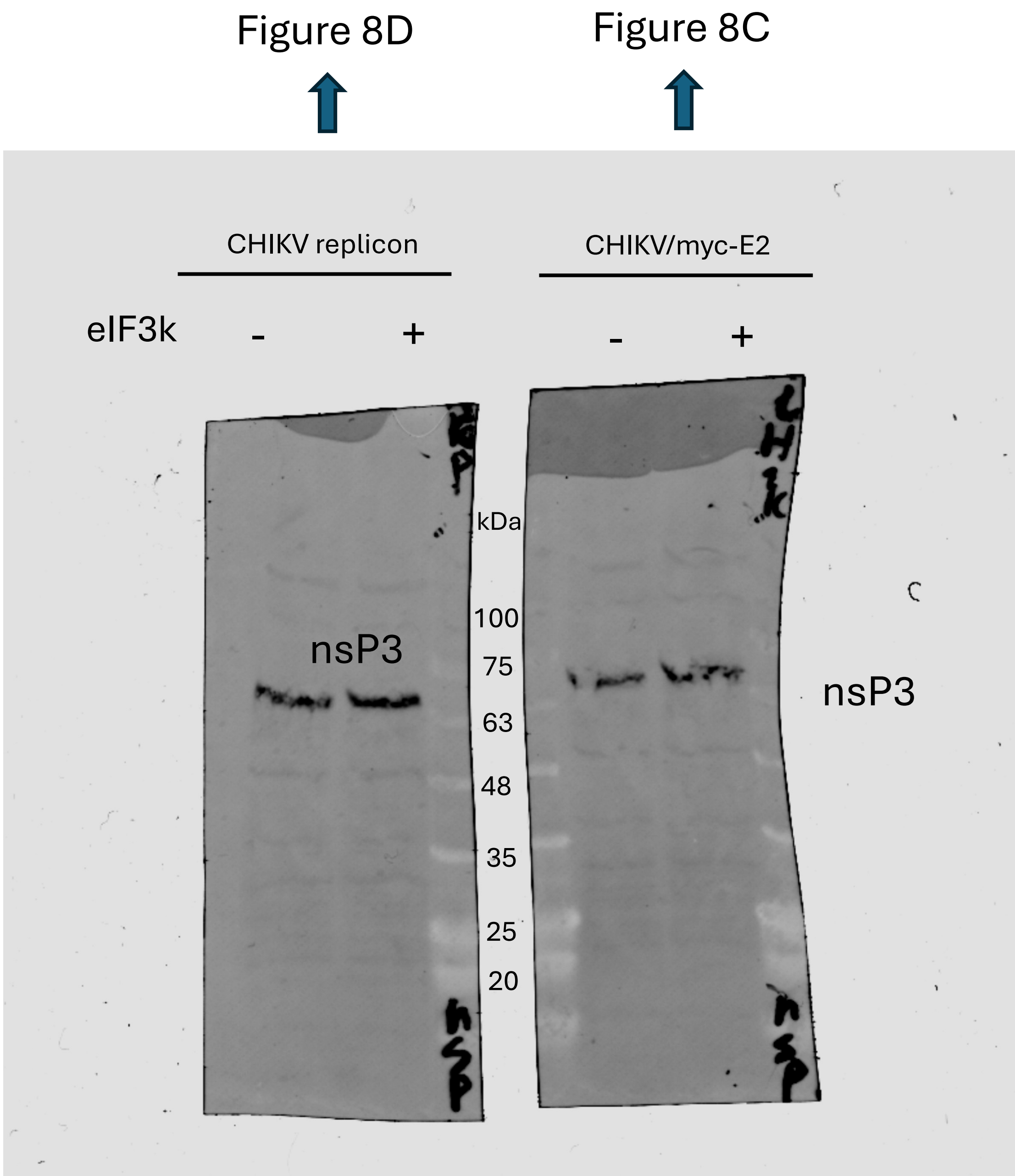
Figure 8D
Figure 8C
CHIKV replicon
CHIKV/myc-E2
eIF3k
-
+
-
+
kDa
100
75
63
48
35
25
20
nsP3
nsP3

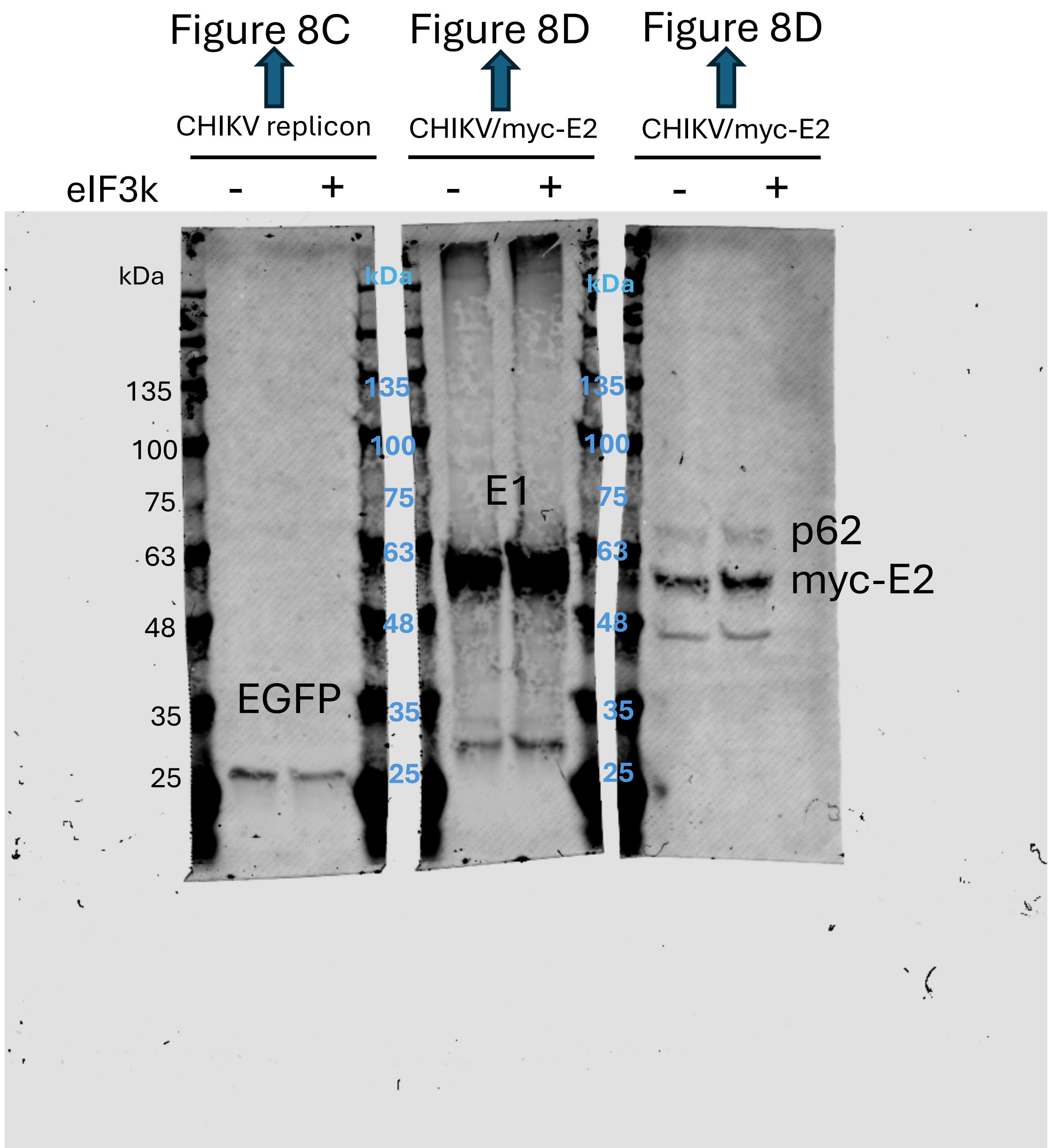
Figure 8C
Figure 8D
Figure 8D
CHIKV replicon
CHIKV/myc-E2
CHIKV/myc-E2
eIF3k
-
+
-
+
-
+
kDa
135
100
75
63
48
35
25
E1
EGFP
p62
myc-E2

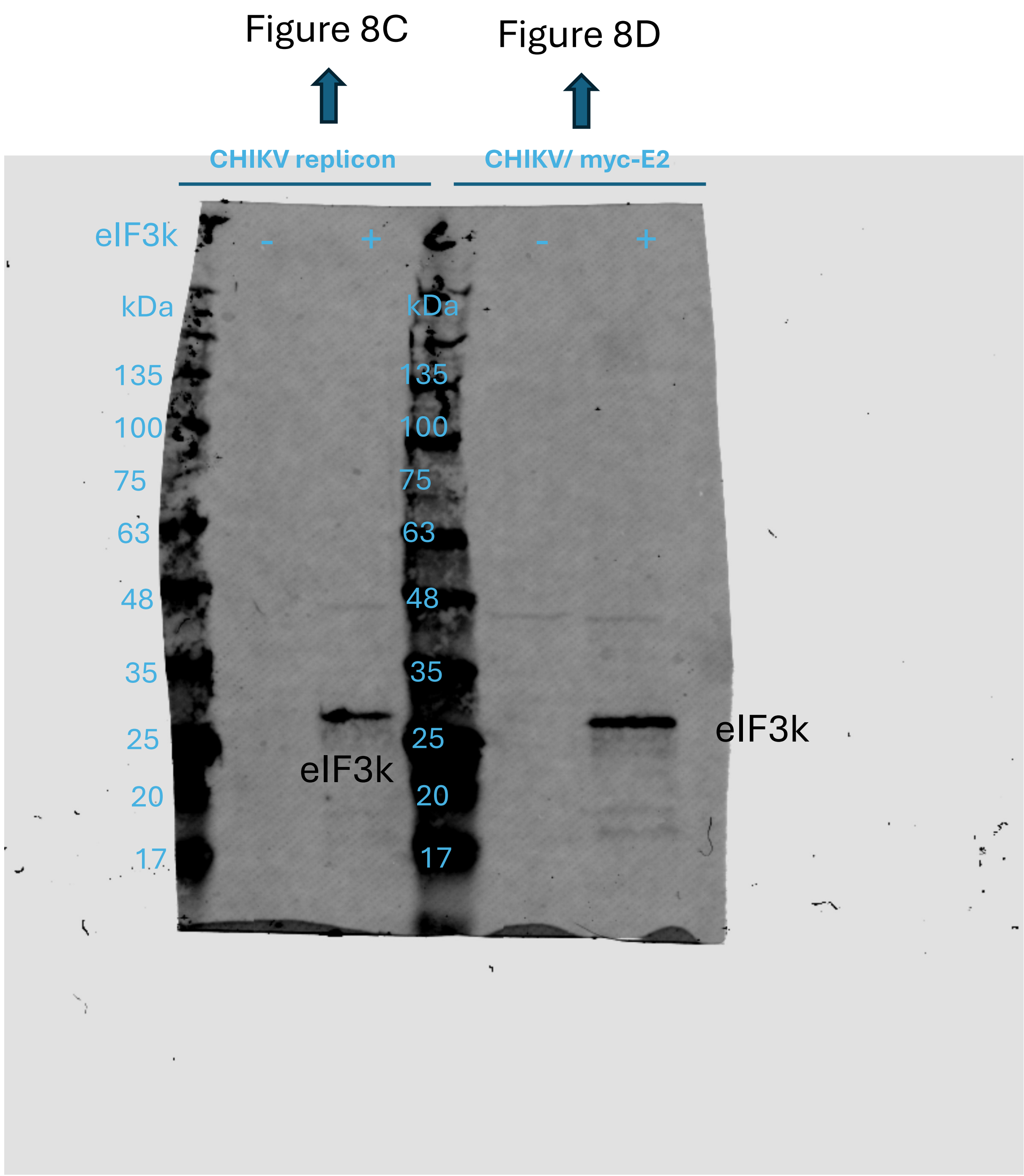
Figure 8C
Figure 8D
CHIKV replicon
CHIKV/ myc-E2
eIF3k
- + - +
kDa
kDa
135
100
75
63
48
35
25
20
17
135
100
75
63
48
35
25
20
17
eIF3k
eIF3k

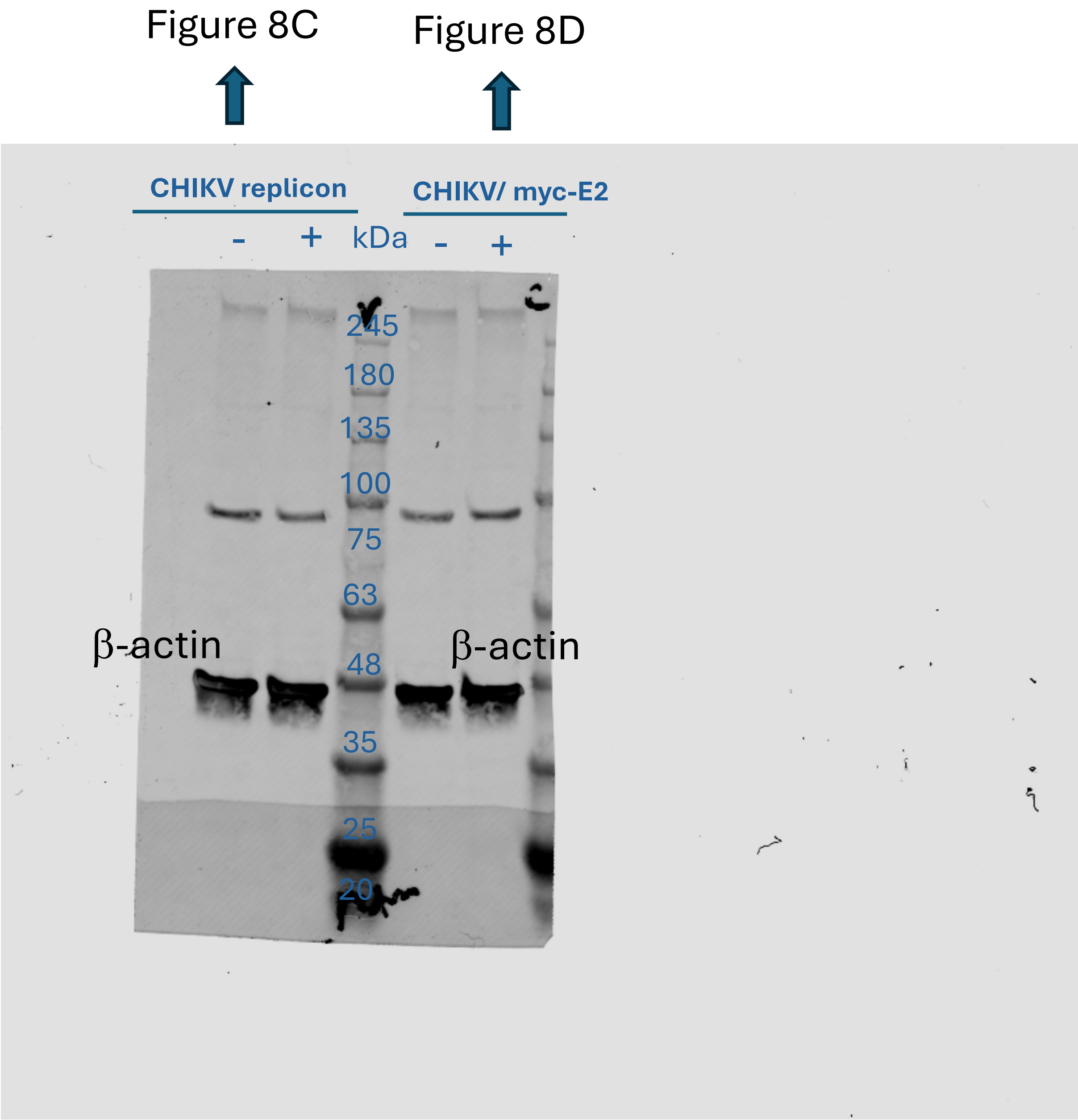
Figure 8C
Figure 8D
CHIKV replicon
CHIKV/ myc-E2
- + kDa - +
245
180
135
100
75
63
48
35
25
20
β-actin
β-actin
