# Supplementary material for: Interaction of chikungunya virus glycoproteins with macrophage factors controls virion production
Source: EMBO J. 2024 Sep 11;43(20):4625–55. doi: 10.1038/s44318-024-00193-3 (PMC11480453; doi:10.1038/s44318-024-00193-3)
Supplement: Supplementary file 11 — Source data Fig. 8 [file 44318_2024_193_MOESM11_ESM.zip › Figure 8/8C-8D/8C-8D images.pptx]

## Slide 1
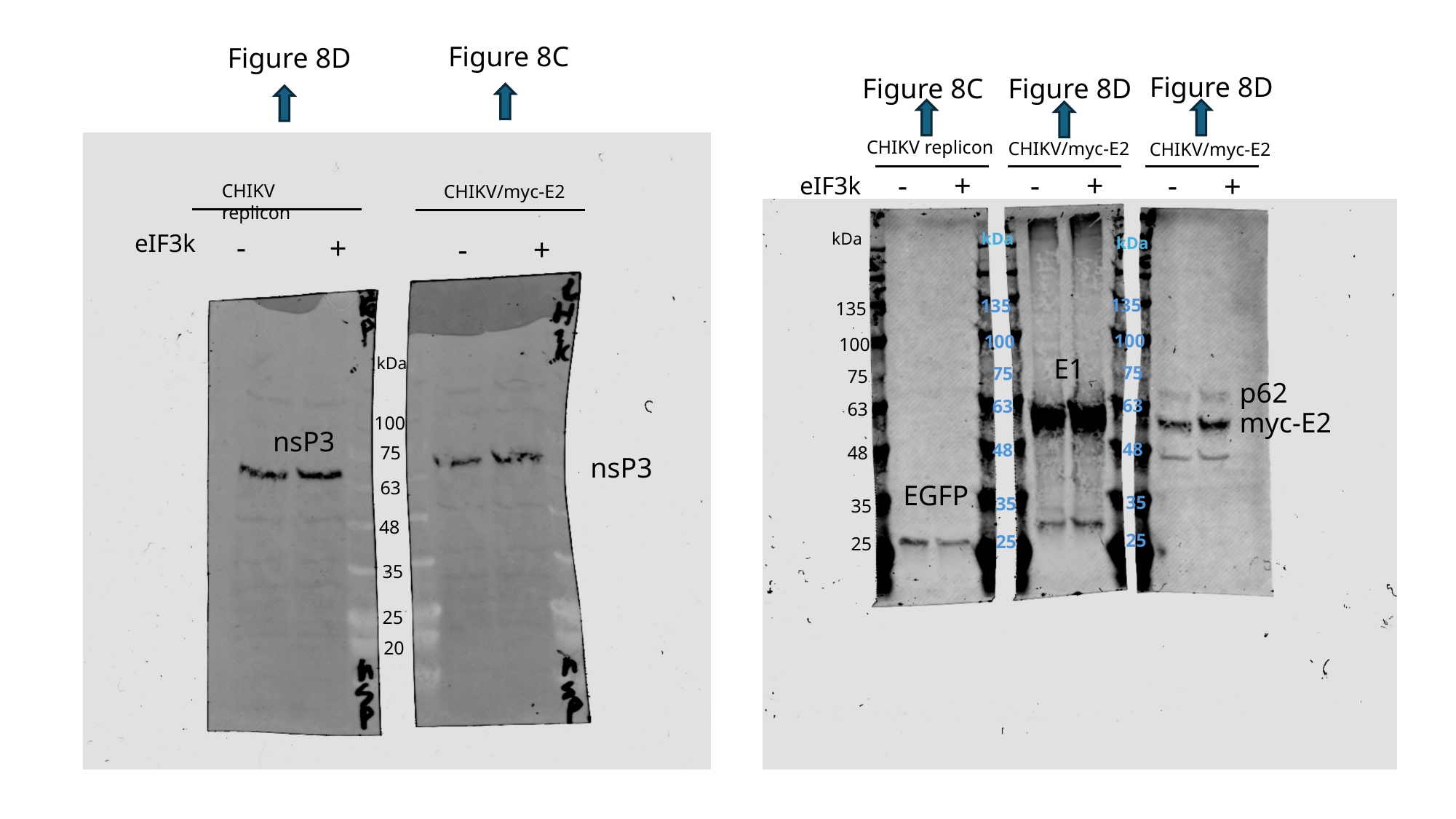

Figure 8C
Figure 8D
Figure 8D
Figure 8D
Figure 8C
CHIKV replicon
CHIKV/myc-E2
CHIKV/myc-E2
-
-
+
+
-
+
eIF3k
CHIKV replicon
CHIKV/myc-E2
eIF3k
kDa
kDa
-
+
-
+
kDa
135
135
135
100
100
100
E1
kDa
75
75
75
p62
63
63
63
myc-E2
100
nsP3
48
48
48
75
nsP3
63
EGFP
35
35
35
48
25
25
25
35
25
20

## Slide 2
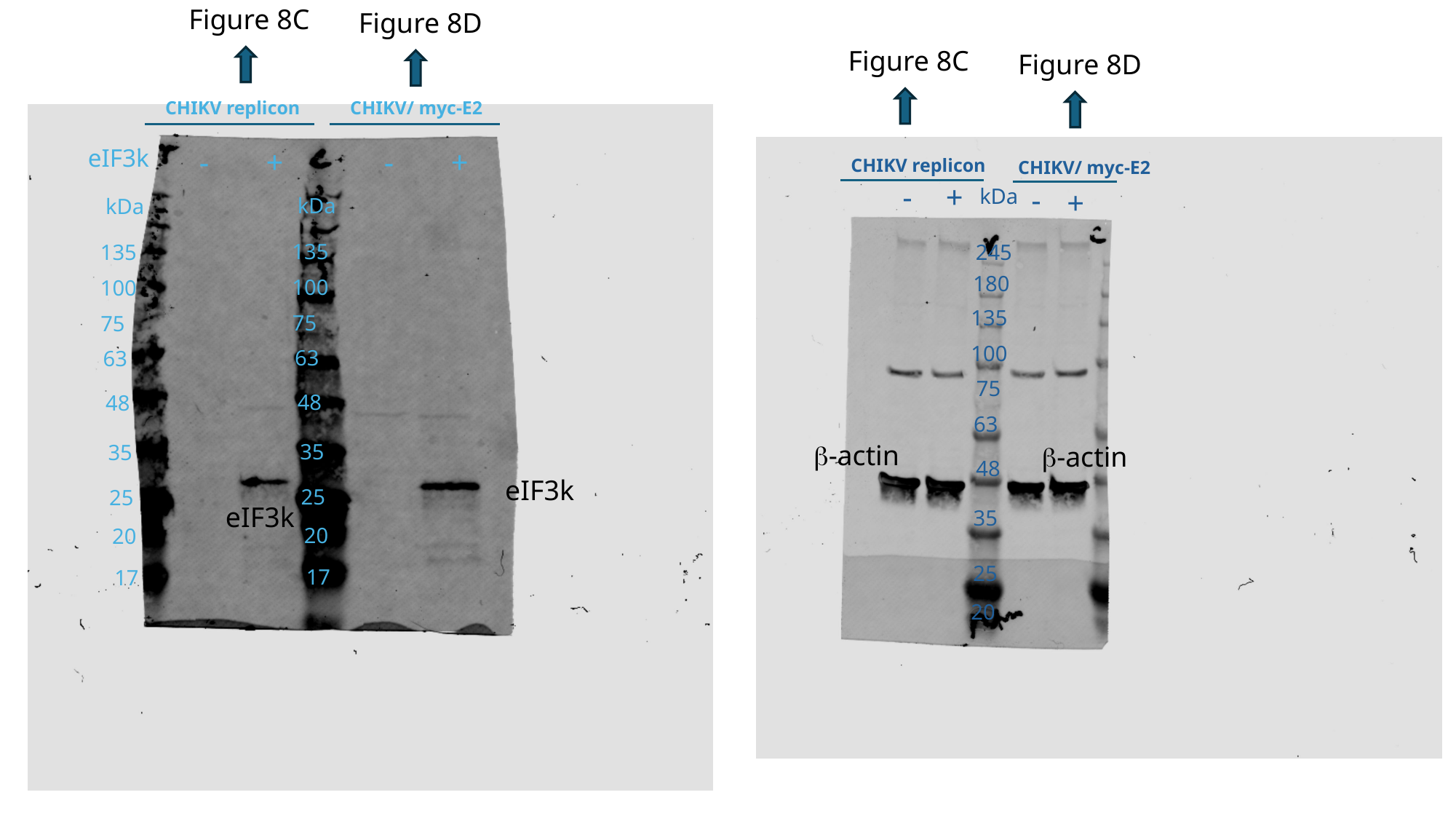

Figure 8C
Figure 8D
Figure 8C
Figure 8D
CHIKV replicon
CHIKV/ myc-E2
eIF3k
-
-
+
+
CHIKV replicon
CHIKV/ myc-E2
-
+
-
kDa
+
kDa
kDa
135
135
245
180
100
100
135
75
75
100
63
63
75
48
48
63
35
b-actin
b-actin
35
48
eIF3k
25
25
eIF3k
35
20
20
25
17
17
20
