# Supplementary material for: Interaction of chikungunya virus glycoproteins with macrophage factors controls virion production
Source: EMBO J. 2024 Sep 11;43(20):4625–55. doi: 10.1038/s44318-024-00193-3 (PMC11480453; doi:10.1038/s44318-024-00193-3)
Supplement: Supplementary file 11 — Source data Fig. 8 [file 44318_2024_193_MOESM11_ESM.zip › Figure 8/8G/8G WB images.pptx]

## Slide 1
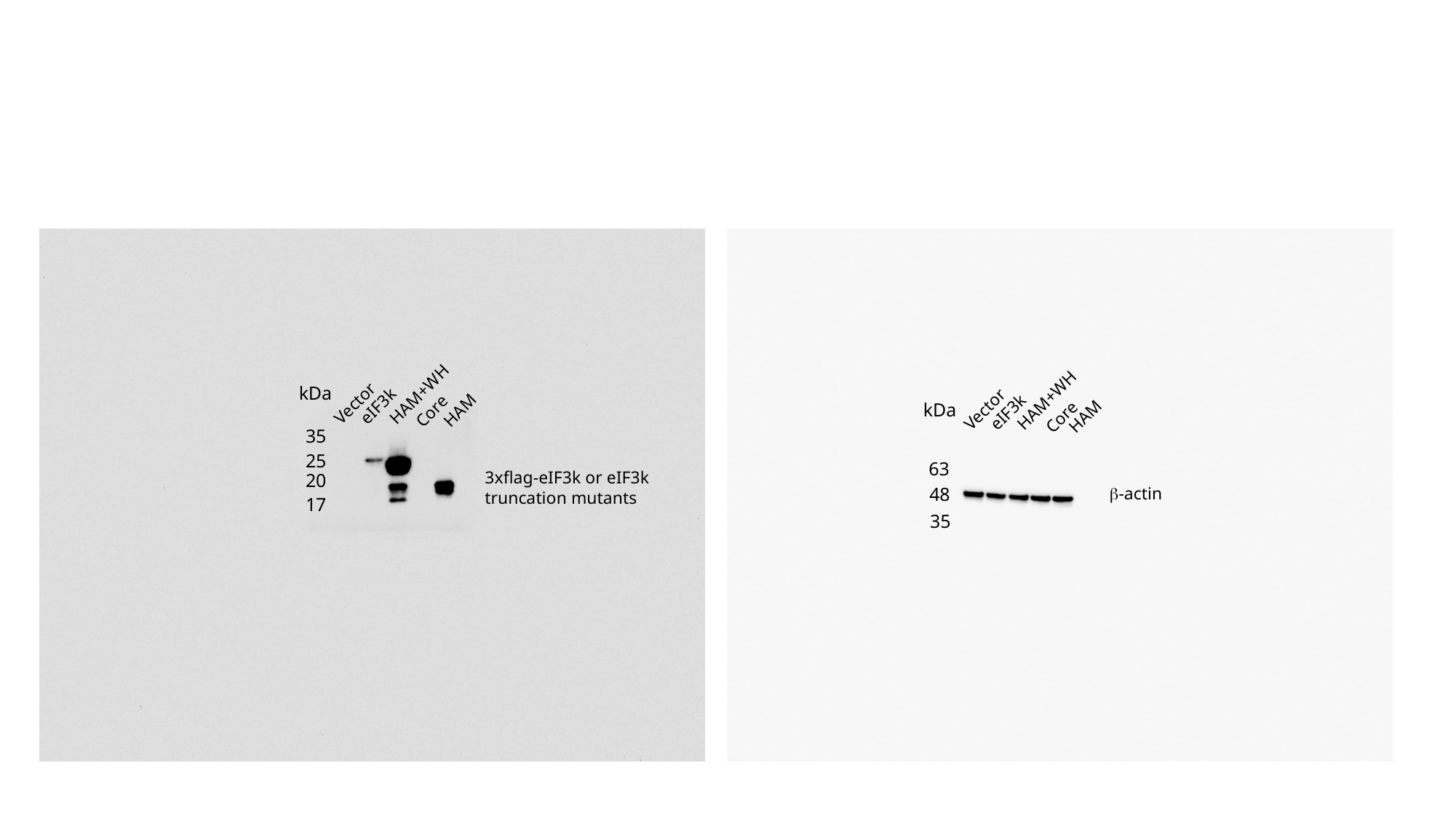

HAM+WH
HAM
Core
HAM+WH
kDa
HAM
Core
eIF3k
Vector
kDa
eIF3k
Vector
35
25
63
3xflag-eIF3k or eIF3k truncation mutants
20
48
b-actin
17
35
