# Supplementary material for: Interaction of chikungunya virus glycoproteins with macrophage factors controls virion production
Source: EMBO J. 2024 Sep 11;43(20):4625–55. doi: 10.1038/s44318-024-00193-3 (PMC11480453; doi:10.1038/s44318-024-00193-3)
Supplement: Supplementary file 12 — Expanded View Figures [file 44318_2024_193_MOESM12_ESM.pdf]

Expanded View Figures

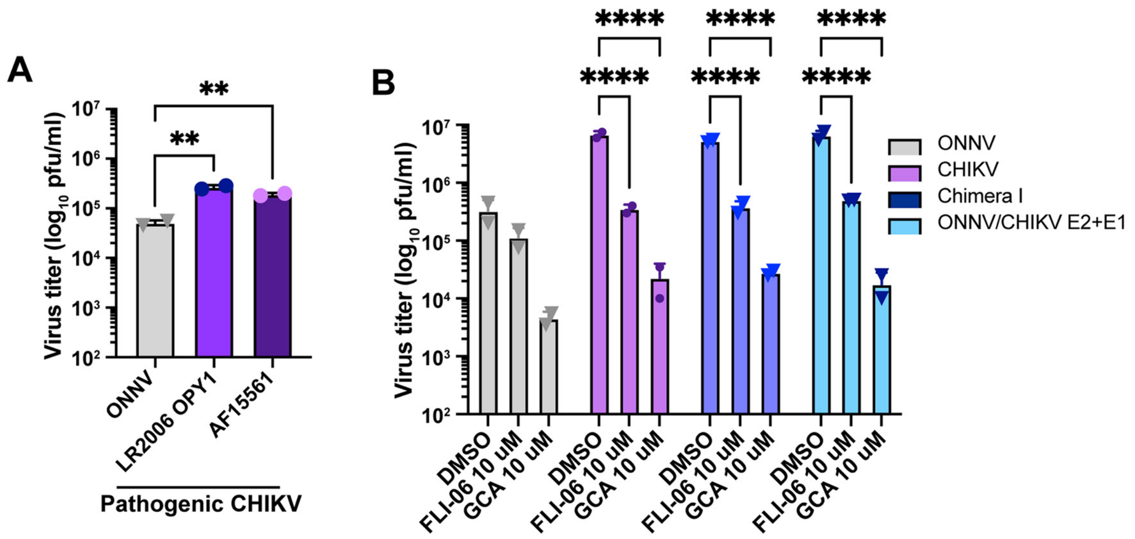

Figure EV1. The advantage of virus production in macrophages is also recapitulated by pathogenic CHIKV and depends more on the host secretory pathway.

(**A**) THP-1-derived macrophages were infected with ONNV SG650, CHIKV La Réunion strain (LR2006 OPY1), and CHIKV Asian strain (AF15561) at MOI 5. Titration of supernatant infectious particles was performed at 24 h.p.i by plaque assay on BHK-21 cells. The incubation period for plaque assay takes 28 h. Data were representative of three independent experiments. Mean values of biological duplicates were plotted with SD. Asterisks indicate statistically significant differences as compared to ONNV (One-way ANOVA and Dunnett's multiple comparisons test: ONNV vs LR2006 OPY1 $**p = 0.0024$; ONNV vs AF15561 $**p = 0.0082$). (**B**) The influence of secretary pathway inhibition on the infections of ONNV, CHIKV, Chimera I, and ONNV/CHIKV E2 + E1. The THP-1-derived macrophages were pretreated with 10 μM FLI-06 or GCA for 30 min prior to 1-h inoculation with ONNV, CHIKV, Chimera I, or ONNV/ CHIKV E2 + E1. The cells were then cultured with the inhibitors at the same concentration (10 μM) for 24 h. The virus titers from supernatants were analyzed by plaque assay as previously described. Data were representative of two independent experiments. Mean values of biological duplicates were plotted with SD. Asterisks indicate statistically significant differences as compared to ONNV (one-way ANOVA and Dunnett's multiple comparisons test: DMSO vs FLI-06/GCA with the infection of CHIKV, Chimera I, or ONNV/CHIKV E2 + E1 $****p < 0.0001$).

 © The Author(s)

A

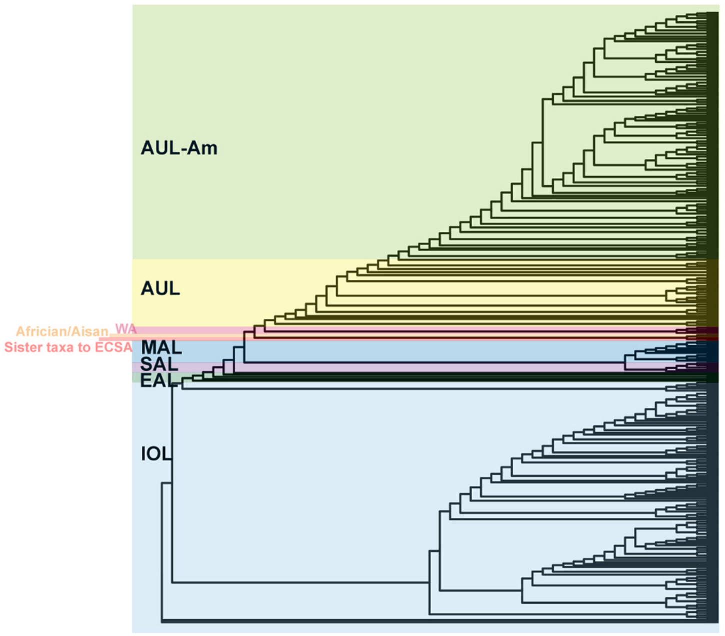

B

AA location (structural polyprotein)	FEL (p<0.05)	MEME (p<0.05)	CHIKV 181/clone 25	ONNV SG650
147		C-147	Ala	Ala
156		C-156	Arg	Arg
460		E2-135	Val	Leu
489	E2-164	E2-164	Ala	Thr
538		E2-213	Thr	Thr
546		E2-221	Lys	Lys
571		E2-246	Ala	Ser
795	6K-47	6K-47	Thr	Thr
847		E1-38	Leu	Leu
954	E1-145	E1-145	Ser	Ser
1020	E1-211	E1-211	Glu	Lye
1029		E1-220	Val	Ile
1163		E1-354	Ile	Ile
1175		E1-366	Arg	Lys

C

	No. of sequences at E2-135	No. of sequences at E1-220
Valine (V)	394	392
Alanine (A)	1	3
Methionine (M)	1	0
Glutamic acid (E)	0	1
Glycine (G)	1	0
Isoleucine (I)	0	1

D

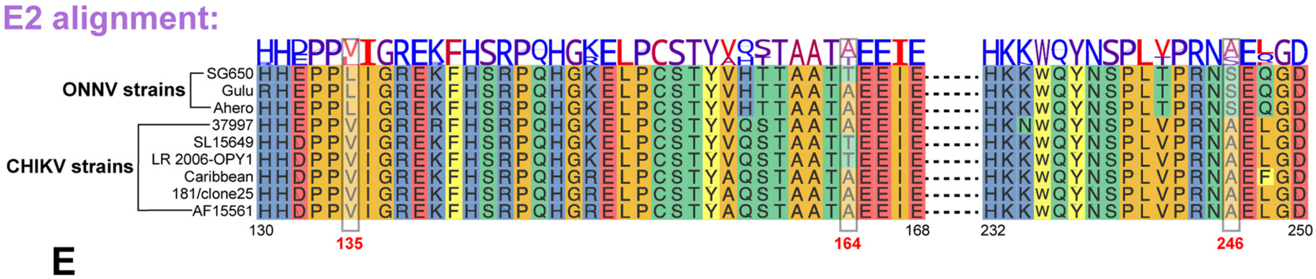

E

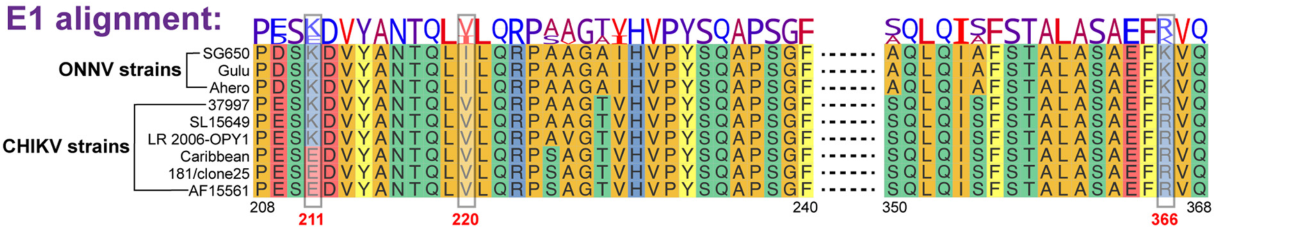

Figure EV2. Evolutionary selection analysis on CHIKV structural proteins.

(**A**) Phylogenetic tree constructed by IQ-tree (Minh et al, 2020) using an alignment of the CHIKV structural polyprotein. The tree was visualized by ggtree (Yu et al, 2017). Tree branches were colored according to the latest CHIKV lineage classification (de Bernardi Schneider et al, 2019) used in CHIKVnext v3 (nextstrain.org/groups/ViennaRNA/CHIKVnext/v3.0). AUL-Am Asian urban + American lineage, AUL Asian urban lineage, EAL Eastern African lineage, IOL Indian Ocean lineage, MAL Middle African lineage, SAL South American lineage, WA Western African lineage. (**B**) Comparison of CHIKV positively selected sites with homologous sites in ONNV. MEME and FEL were used to analyze the positively selected sites in CHIKV structural proteins and generate *P* values. The *P* values are corrected with Benjamini–Hochberg. The positively selected CHIKV amino acids that are different from the homologous residues in ONNV were colored in red and highlighted in gray. (**C**) The heterogeneity of residues at E2-135 and E1-220 in 397 CHIKV patient isolates from NCBI Virus database. (**D**) The E2 alignment of different ONNV and CHIKV strains to compare the amino acid residues at E2-135, E2-164, and E2-246. CHIKV 37997 belongs to the West African lineage. CHIKV LR2006 OPY1 and CHIKV SL15649 belong to the East/Central/South African (ECSA) lineage. CHIKV Caribbean and CHIKV AF15561 belong to the Asian lineage. CHIKV AF15561 is the parental strain of CHIKV vaccine strain 181/clone 25. The alignment is visualized through ggmsa (Zhou et al, 2022). (**E**) The E1 alignment of different ONNV and CHIKV strains to compare the amino acid residues at E1-211, E1-220, and E1-366.

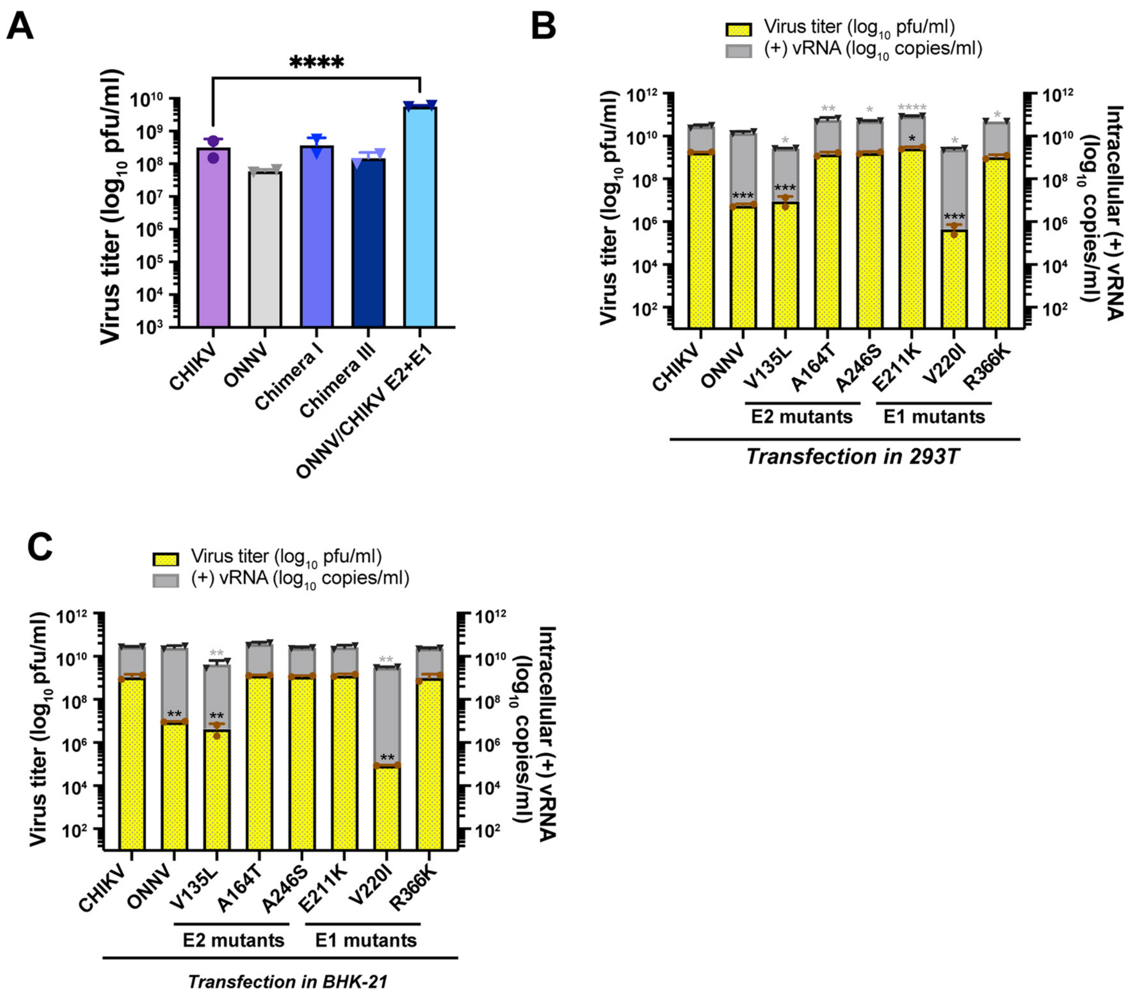

Figure EV3. The superior virus production conferred by CHIKV structural proteins is macrophage-specific.

(**A**) CHIKV, ONNV, Chimera I, Chimera III, and ONNV/CHIKV E2 + E1 infection in 293T cells. Virion production in the supernatant of infected 293T cells was titrated through plaque assay on BHK-21 cells as previously described. Mean values of biological duplicates were plotted with SD. Data were representative of two independent experiments. Asterisks indicate statistically significant differences as compared to CHIKV (one-way ANOVA and Dunnett's multiple comparisons test: CHIKV vs ONNV/CHIKV E2 + E1 ****$p < 0.0001$). (**B**, **C**) Infection of 293T (**B**) and BHK-21 (**C**) cells with CHIKV vaccine strain 181/clone 25 positive selection site mutants. Viral replication and production of positive selection site mutants (E2-V135L, E2-A164T, E2-A246S, E1-E211K, E1-V220I, and E1-R366K) were determined by levels of intracellular (+) vRNAs and secreted infectious particles as previously described. For EV3B, data were representative of two independent experiments. The plaque assay results were plotted from biological duplicates with the mean values. Error bars represent SD (one-way ANOVA and Dunnett's multiple comparisons test: viral titer of CHIKV vs ONNV ***$p = 0.0004$; viral titer of CHIKV vs E2-V135L ***$p = 0.0004$; viral titer of CHIKV vs E1-E211K *$p = 0.017$; viral titer of CHIKV vs E1-V220I ***$p = 0.0004$). The qPCR results were plotted from biological duplicates with the mean values. Error bars represent SD (one-way ANOVA and Brown-Forsythe test: viral copies of CHIKV vs E2-V135L *$p = 0.0116$; viral copies of CHIKV vs E2-A164T **$p = 0.0036$; viral copies of CHIKV vs E2-A246S *$p = 0.0156$; viral copies of CHIKV vs E1-E211K ****$p < 0.0001$; viral copies of CHIKV vs E1-V220I *$p = 0.011$; viral copies of CHIKV vs E1-R366K *$p = 0.0274$). For EV3C, data were representative of two independent experiments. The plaque assay results were plotted from biological duplicates with the mean values. Error bars represent SD (one-way ANOVA and Dunnett's multiple comparisons test: viral titer of CHIKV vs ONNV **$p = 0.006$; viral titer of CHIKV vs E2-V135L **$p = 0.0058$; viral titer of CHIKV vs E1-V220I **$p = 0.0057$). The qPCR results were plotted from biological duplicates with the mean values. Error bars represent SD (one-way ANOVA and Brown-Forsythe test: viral copies of CHIKV vs E2-V135L **$p = 0.0027$; viral copies of CHIKV vs E1-V220I **$p = 0.0019$).

 © The Author(s)

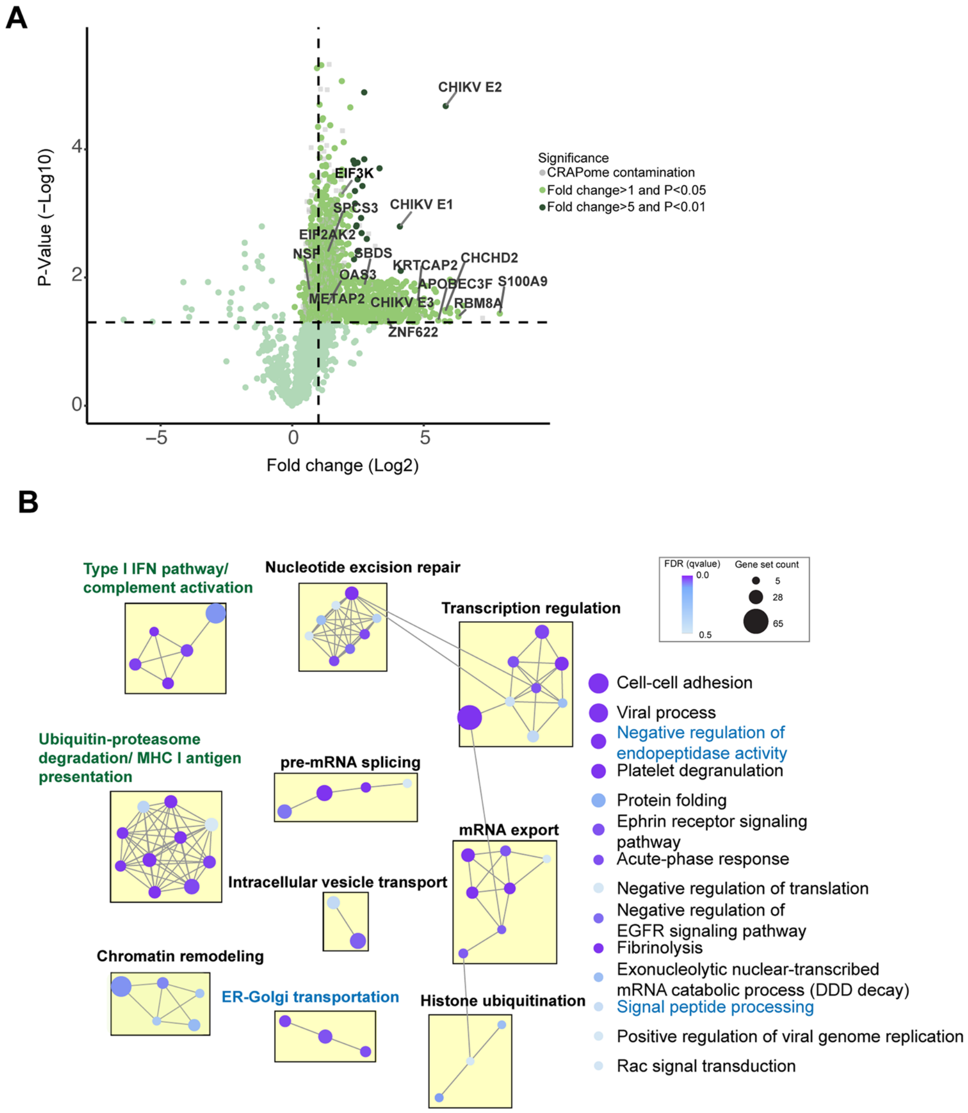

Figure EV4. The macrophage host factors identified by AP-MS and representative biological processes of significantly enriched host factors.

(**A**) Volcano plot depicting cellular interactors of CHIKV glycoproteins identified by mass spectrometry. A moderated *t*-test from R package ArtMS3 was used to generate the *P* values which were adjusted with Benjamini–Hochberg for the multiple hypothesis correction. The volcano plot is scattered by $-\log_{10} P$ value (y-axis) and $\log_2$ expression fold change (FC) of proteins co-immunoprecipitated from CHIKV/myc-E2 infected cells with respect to the proteins from CHIKV WT infected cells (x-axis). The dashed cut-offs of the adjusted *P* value and expression fold change are 0.05 ($-\log_{10} P$ value = 1.30103) and 2 ($\log_2$FC = 1), respectively. CHIKV glycoproteins (E3, E2, E1) and host factors for further investigation in Fig. 6A are annotated here. (**B**) Enrichment map that summarizes over-represented biological processes of identified host factors in groups. The enriched proteins identified by mass spectrometry were clustered by biological processes and organized into a network with edges connecting overlapping gene sets to reveal functional modules.

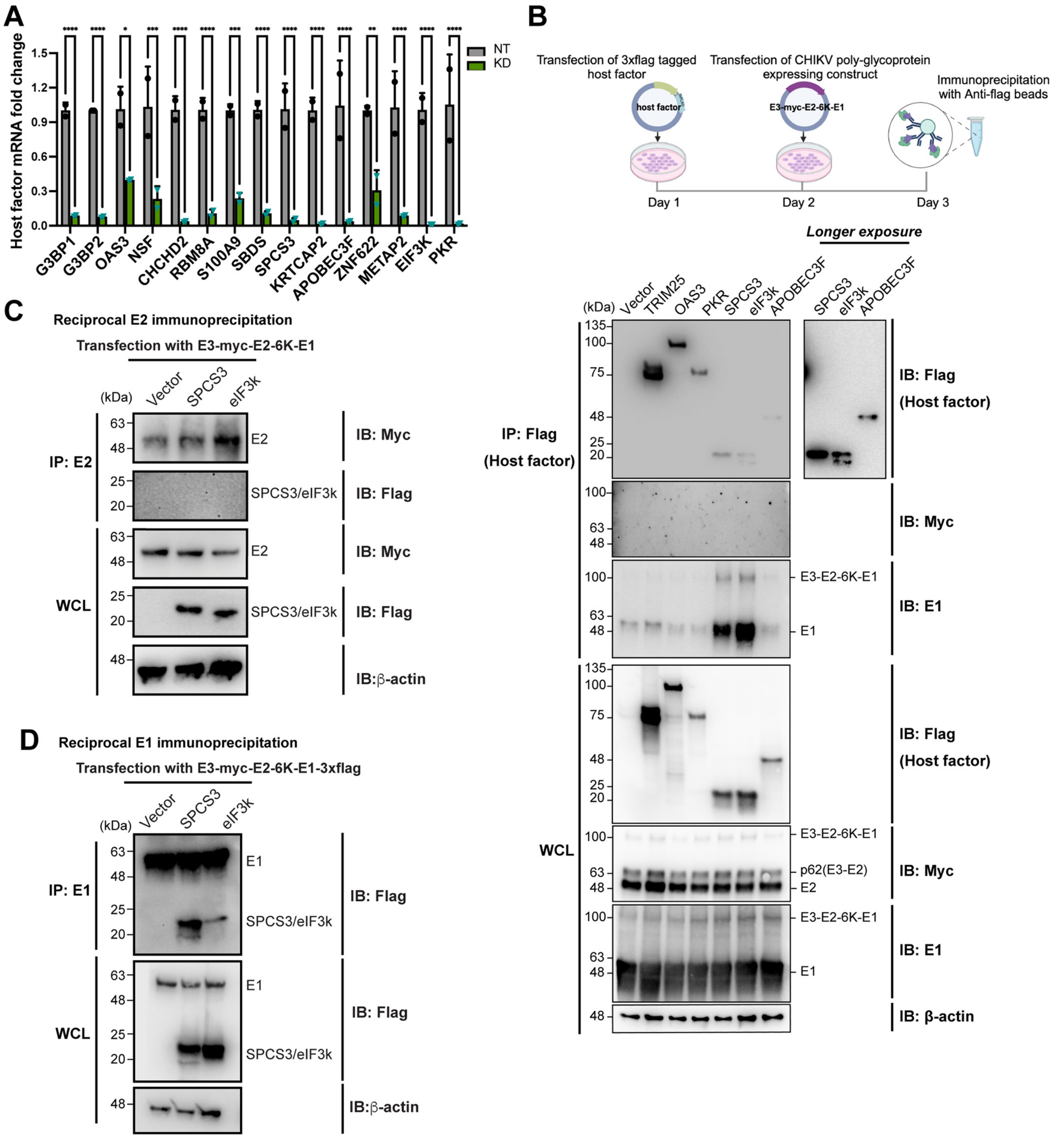
A
Host factor mRNA fold change
NT
KD
G3BP1
G3BP2
OAS3
NSF
CHCHD2
RBM8A
S100A9
SBDS
SPCS3
KRTCAP2
APOBEC3F
ZNF622
METAP2
EIF3K
PKR
B
Transfection of 3xflag tagged host factor
Transfection of CHIKV poly-glycoprotein expressing construct
Immunoprecipitation with Anti-flag beads
host factor
E3-myc-E2-6K-E1
Day 1
Day 2
Day 3
Longer exposure
(kDa)
Vector
TRIM25
OAS3
PKR
SPCS3
eIF3k
APOBEC3F
IP: Flag
(Host factor)
IB: Flag
(Host factor)
IB: Myc
E3-E2-6K-E1
E1
IB: E1
WCL
p62(E3-E2)
E2
IB: β-actin
C
Reciprocal E2 immunoprecipitation
Transfection with E3-myc-E2-6K-E1
IP: E2
SPCS3/eIF3k
IB: Myc
IB: Flag
IB:β-actin
D
Reciprocal E1 immunoprecipitation
Transfection with E3-myc-E2-6K-E1-3xflag
IP: E1

 © The Author(s)

Figure EV5. New antiviral host factors, SPCS3 and eIF3k, specifically interact with CHIKV E1.

(**A**) The macrophages were transfected with 25 nM nontargeting siRNAs (NT) or pooled siRNAs targeting host factors (G3BP1, G3BP2, OAS3, NSF, CHCHD2, RBM8A, S100A9, SBDS, SPCS3, KRTCAP2, APOBEC3F, ZNF622, METAP2, EIF3K, and PKR). mRNAs of cells treated with siRNAs were extracted 48 h post transfection for RT-qPCR to evaluate the host factor knockdown efficiencies. Data were representative of two independent experiments. The mean values of biological duplicates were plotted with SD (two-way ANOVA and Šidák's multiple comparisons test: si-NT vs si-OAS3 *$p = 0.0118$; si-NT vs si-ZNF622 **$p = 0.0026$; si-NT vs si-NSF ***$p = 0.0006$; si-NT vs si-S100A9 ***$p = 0.0009$; si-NT vs si-G3BP1/G3BP2/CHCHD2/RBM8A/SBDS/SPCS3/KRTCAP2/APOBEC3F/METAP2/EIF3K/PKR ****$p < 0.0001$). (**B**) 293T cells were transfected with plasmids expressing 3xflag-tagged host factors (TRIM25, OAS3, SPCS3, APOBEC3F, eIF3k, and PKR) or empty vector control for 24 h, and later transfected with plasmid expressing CHIKV glycoproteins (E3-myc-E2-6K-E1). The cells were lysed and immunoprecipitated by anti-flag agarose beads. Immunoblot was probed to check for E2/E1 binding to these host factors. TRIM25-3xflag was transfected into 293T cells for immunoprecipitation control. Data were representative of three independent experiments. (**C**) 293T cells were transfected with plasmids expressing 3xflag-tagged host factors (SPCS3, eIF3k) or empty vector control for 24 h, followed by transfection with the plasmid expressing CHIKV E3-myc-E2-6K-E1. The cells were lysed for immunoprecipitation with Dynabeads Protein G conjugated with E2 antibody (CHK-48) (Fox et al, 2015). Immunoblot was probed for host factor (SPCS3, eIF3k) binding to E2. Data were representative of two independent experiments. (**D**) 293T cells were transfected with plasmids expressing 3xflag-tagged host factors (SPCS3, eIF3k) or empty vector control for 24 h, followed by transfection with the plasmid expressing CHIKV E3-myc-E2-6K-E1-3xflag. The cells were lysed for immunoprecipitation with Dynabeads Protein G conjugated with E1 antibody. Immunoblot was probed for host factor (SPCS3, eIF3k) binding to E1. Data were representative of two independent experiments.

© The Author(s)
